# Integrative analysis of m6A-SNPs and single-cell RNA sequencing reveals key drivers of endocrine combined with CDK4/6 inhibitor therapy resistance in ER+ breast cancer

**DOI:** 10.3389/fphar.2025.1590363

**Published:** 2025-04-15

**Authors:** Ruijie Ming, Han Zhang, Huan Wu, Fangbiao Zhan, Xiaoping Huang, Huawen Liu

**Affiliations:** ^1^ Department of Oncology, Chongqing University Three Gorges Hospital, Chongqing, China; ^2^ School of Medicine, Chongqing University, Chongqing, China; ^3^ Department of Orthopedics, Chongqing University Three Gorges Hospital, Chongqing, China

**Keywords:** m6A methylation, single nucleotide polymorphism, CDK4/6 inhibitor, ER+ breast cancer, therapy resistance

## Abstract

**Background:**

Endocrine therapy combined with CDK4/6 inhibitors remains a standard treatment for ER+ breast cancer, yet resistance is a prevalent challenge. This study explores the role of N6-methyladenosine (m6A) modifications, influenced by m6A-SNPs, in shaping therapy resistance, utilizing single-cell RNA sequencing to delineate the underlying molecular mechanisms.

**Methods:**

We integrated genome-wide association study data with single-cell transcriptomic profiles from ER+ breast cancer patients, focusing on differences between resistant and sensitive responses to CDK4/6 inhibitors. m6A-SNPs were identified and analyzed for their impact on gene expression and interactions with RNA-binding proteins, with a particular focus on their roles within key cellular pathways.

**Results:**

The study identified crucial m6A-SNPs associated with therapy resistance. Notably, changes in the expression of FILIP1L and TOM1L1, related to these SNPs, were mapped using pseudotime trajectory analysis, which traced the evolution from sensitive to resistant cellular states. FILIP1L and TOM1L1 exhibited dynamic expression changes along the trajectory, correlating with significant shifts in cell fate decisions. These findings underscore their potential roles as mediators in the development of resistance, particularly through their involvement in the PI3K-Akt and Wnt signaling pathways, critical in cancer progression and drug resistance.

**Conclusion:**

Our findings emphasize the importance of m6A-SNPs in influencing resistance to therapy in ER+ breast cancer. The dynamic regulation of FILIP1L and TOM1L1 along the developmental trajectory of tumor cells from sensitivity to resistance provides insights into the molecular complexity of therapy resistance. These results pave the way for developing targeted therapies that modify m6A-driven pathways, offering new strategies to counteract resistance and improve patient outcomes.

## 1 Introduction

Breast cancer is now the most commonly diagnosed malignancy and remains a significant cause of cancer-related mortality worldwide among women (Siegel et al., 2024). The subtype characterized by the overexpression of estrogen receptors (ER+ breast cancer) accounts for approximately 70% of all breast cancer cases and has seen significant treatment advancements due to the introduction of combined therapy of endocrine and CDK4/6 inhibitor (Blakely et al., 2023). These treatments primarily target estrogen signaling and other critical cellular pathways, markedly improving clinical outcomes (Glaviano et al., 2024; Lloyd et al., 2024). However, despite initial therapeutic success, resistance to endocrine therapy is common, particularly in metastatic breast cancer, where most patients eventually develop resistance, leading to recurrence and poor prognosis (Hanker et al., 2020; Glaviano et al., 2024; Zheng et al., 2024).

The challenge of CDK4/6 inhibitor resistance necessitates a deeper understanding of the underlying molecular mechanisms driving this phenomenon. Recently, the post-transcriptional RNA modifications, specifically N6-methyladenosine (m6A) methylation, known to regulate mRNA stability, translation, and splicing, has emerged as influential in cancer progression and treatment responses (Deng et al., 2023; Wen et al., 2023). Specifically, single nucleotide polymorphisms (SNPs) that affect m6A modifications (m6A-SNPs) have recently emerged as important factors in multiple diseases including cancer (Lv et al., 2022; Wu et al., 2022). Nevertheless, the involvement of m6A modifications in resistance to CDK4/6 inhibitors in ER+ breast cancer remains unclear (Wang D. et al., 2023; Zhuang et al., 2023).

Moreover, the advent of single-cell RNA sequencing (scRNA-seq) technology has enabled unprecedented insights into the cellular heterogeneity of tumors, allowing the identification of distinct subpopulations of tumor cells that may drive resistance to therapy (Zhang et al., 2021). Integrating genomic data with single-cell transcriptomic profiles holds great promise for uncovering the molecular basis of resistance and identifying potential therapeutic targets.

This study aims to elucidate the role of m6A-SNPs in endocrine therapy resistance in ER+ breast cancer by employing a two-pronged approach. First, we identified breast cancer-associated m6A-SNPs by integrating GWAS summary data with m6A modification profiles from the RMVar database, focusing on SNPs with significant regulatory potential. Second, we utilized scRNA-seq to analyze the heterogeneity of tumor cells, distinguishing between resistant and sensitive subpopulations based on their response to CDK4/6 inhibitor therapy. By combining these genomic and transcriptomic analyses, we aimed to uncover key molecular pathways and gene regulatory networks driving resistance, providing insights into potential therapeutic targets for overcoming resistance in breast cancer treatment.

## 2 Materials and methods

### 2.1 Data sources

In this study, we employed several publicly available databases to gather genomic and transcriptomic data for analysis. ER+ Breast cancer genome-wide association study (GWAS) summary statistics were obtained from the IEU database (ieu-a-1127, https://gwas.mrcieu.ac.uk), which includes 69,501 breast cancer cases and 105,974 controls of Europeans (Michailidou et al., 2017). The RMVar database was used to identify m6A-SNPs, which provides comprehensive information on RNA modifications and associated genetic variants (https://www.rmvar.renlab.org) (Luo et al., 2021). Additionally, we retrieved single-cell RNA sequencing (scRNA-seq) data from the Gene Expression Omnibus (GEO) database (https://www.ncbi.nlm.nih.gov/geo) under accession number GSE158724, which includes data from 41 ER+ breast cancer patients treated with a combination of endocrine therapy and CDK4/6 inhibitor (Griffiths et al., 2021).

Bulk transcriptomic data of ER+ breast cancer were obtained from the TCGA breast cancer (TCGA-BRCA) cohort through the R package TCGAbiolinks (Colaprico et al., 2016). Expression levels were normalized using log2(FPKM +1) transformation. To ensure consistency with the single-cell dataset, we included only ER+ breast cancer patients with complete overall survival (OS) information and corresponding normal control samples. As the dataset was derived from processed expression matrices, no raw sequencing read filtering was required.

### 2.2 Identification of m6A-SNPs

As previously mentioned in earlier studies, we identified m6A-SNPs associated with ER+ breast cancer by examining the intersection of SNPs with p < 5e-8 between ER+ breast cancer GWAS datasets and m6A-SNPs listed in the RMVar database (Xuan et al., 2021). We further filtered for m6A-SNPs that demonstrated expression quantitative trait loci (eQTL) signals using the HaploReg database (http://compbio.mit.edu/HaploReg), which provides information on the regulatory potential of genetic variants (Ward and Kellis, 2012; Ward and Kellis, 2016). HaploReg database utilizes linkage disequilibrium (LD) information from the 1000 Genomes Project to enable SNP linkage analyses.

### 2.3 Interaction between m6A-SNPs and RNA-Binding proteins

The UCSC Genome Browser (GRCh37/hg19; https://genome.ucsc.edu/) hosts an extensive compendium of annotations and components for vertebrate and model organism genomes (Lee et al., 2020; Nassar et al., 2023). To analyze the potential functional impacts of m6A-SNPs, we submitted those m6A-SNPs that exhibited eQTL signals to the UCSC Genome Browser. We used this platform to systematically predict and visualize the genomic regions where modifications due to m6A-SNPs are likely to occur, and to infer the consequential changes in RNA-protein interactions.

### 2.4 Processing and pretreatment of single cell transcriptome data

#### 2.4.1 Selection of single-cell samples

The GSE158724 dataset contained single-cell transcriptomic profiles from 41 estrogen receptor-positive (ER+) breast cancer patients treated with neoadjuvant endocrine therapy (letrozole) and/or CDK4/6 inhibitor (ribociclib), totaling 95 samples. For this analysis, 12 samples with available gene expression data were selected corresponding to the intermittent high-dose combination therapy group (letrozole + ribociclib), comprising six responders (R) and six non-responders (NR) based on their clinical outcomes.

#### 2.4.2 Single-cell RNA-seq quality control and normalization

Cause this dataset only provided the transcriptomic profiles of tumor cells, we focused solely on tumor cells, which were preprocessed as follows. According to the dataset description, all cells underwent a rigorous preprocessing pipeline that classified them as tumor cells, so no further cell- or gene-level filtering was conducted in this study. The raw scRNA-seq data were normalized using the NormalizeData function in the Seurat R package, which scales the gene expression counts by total expression per cell, followed by log transformation (Gribov et al., 2010). To account for variability in gene expression, the FindVariableFeatures function was employed to identify the top 3,000 highly variable genes across all cells using the “vst” method. These genes are critical for downstream analysis as they capture the biological variation in the dataset. Data scaling was performed with the ScaleData function.

Principal component analysis (PCA) was performed using the RunPCA function in Seurat, with the top 30 principal components (PCs) chosen based on the ElbowPlot visualization. To address potential batch effects and harmonize the data across samples, the Harmony algorithm was applied (Korsunsky et al., 2019). Harmony adjusts for batch-to-batch variability by aligning gene expression profiles across different patients, ensuring that biological signals are not confounded by technical artifacts. Following batch correction, a shared nearest neighbor (SNN) graph was constructed using the FindNeighbors function with the top 30 PCs. Cells were clustered into distinct subgroups using the FindClusters function, with a resolution parameter of 0.05 to define six distinct clusters. The clusters were visualized using uniform manifold approximation and projection (UMAP) plots, generated with the RunUMAP function (with n. neighbors = 30 and dims = 1:30), which provided a low-dimensional representation of the cellular landscape and allowed for clear distinction between the cell populations.

### 2.5 Differential expression and metabolic pathway analysis

To investigate gene expression differences between resistant (Tumor_Res) and sensitive (Tumor_Sen) tumor cells, we performed differential expression analysis using the limma package. Genes with an absolute log2 fold change greater than 0.8 and an adjusted p-value less than 0.05 were considered differentially expressed. Gene Ontology (GO) and Kyoto Encyclopedia of Genes and Genomes (KEGG) pathway enrichment analyses were conducted using the clusterProfiler R package to identify pathways enriched among differentially expressed genes (Kanehisa and Goto, 2000; Kanehisa et al., 2017; Wu et al., 2021). Metabolic pathway analysis was performed using the GSVA R package (Hänzelmann et al., 2013), and metabolic flux analysis was conducted using single-cell flux estimation analysis (scFEA), a tool designed to infer the metabolic activity of cells based on single-cell transcriptomic data (Alghamdi et al., 2021).

### 2.6 Identification and analysis of drug-resistant subpopulations

Tumor cells were classified into drug-resistant and sensitive subpopulations based on the ratio of NR (Non-responder) and R (Responder) cell sources. The R package oncoPredict was used to calculate the IC50 value of ribociclib drugs for each tumor cell and analyze the difference between the IC50 values of drugs in the drug-resistant vs non-resistant subpopulations (Maeser et al., 2021). Then, we divided each cluster into the R or NR group according to whether it contained a significantly higher proportion of R or NR cells (Ren et al., 2025).

Based on the TCGA-BRCA cohort, the score of resistant subpopulations was calculated based on GSVA method, and the samples were grouped according to the score to show the prognostic Kaplan-Meier survival curve (OS). GSVA quantified each tumor cell subpopulation to calculate the score of KEGG metabolic pathway, and the drug-resistant subpopulation vs non-resistant subpopulation was analyzed comparatively, to identify the significant metabolic pathway.

Metabolite abundance was calculated for each tumor cell subpopulation using scFEA to identify the enrichment of metabolic pathway products in each subpopulation. Spearman was used to calculate the correlation between the relevant metabolite levels and the IC50 of ribociclib.

### 2.7 Pseudotime trajectory and cell fate analysis

We employed pseudotime trajectory analysis using the monocle R package to explore the developmental trajectory of tumor cells from a sensitive to a resistant state (Trapnell et al., 2014). Cells were ordered along the pseudotime trajectory, and key transition points were identified. To further investigate the molecular mechanisms driving this transition, we performed branch expression analysis modeling (BEAM) to identify genes with branch-specific expression patterns. These genes were then subjected to GO and KEGG pathway enrichment analyses to identify the biological processes associated with the transition to resistance.

### 2.8 Protein-protein interaction (PPI) network analysis

To explore the interactions between differentially expressed genes and m6A-SNP-associated genes, we constructed a protein-protein interaction (PPI) network using the STRING database (Szklarczyk et al., 2023). Cytoscape software was used to visualize the network and identify hub genes that may play key roles in the development of endocrine therapy resistance (Doncheva et al., 2019).

### 2.9 Statistical analysis

All statistical analyses were conducted using R software (v4.1.2). Differential expressions were determined using moderated t-statistics, and significance was defined as p < 0.05. Adjustments for multiple testing were made using the Benjamini–Hochberg procedure to control the false discovery rate (FDR).

## 3 Results

### 3.1 Identification of m6A-SNPs associated with breast cancer

**FIGURE 1:**
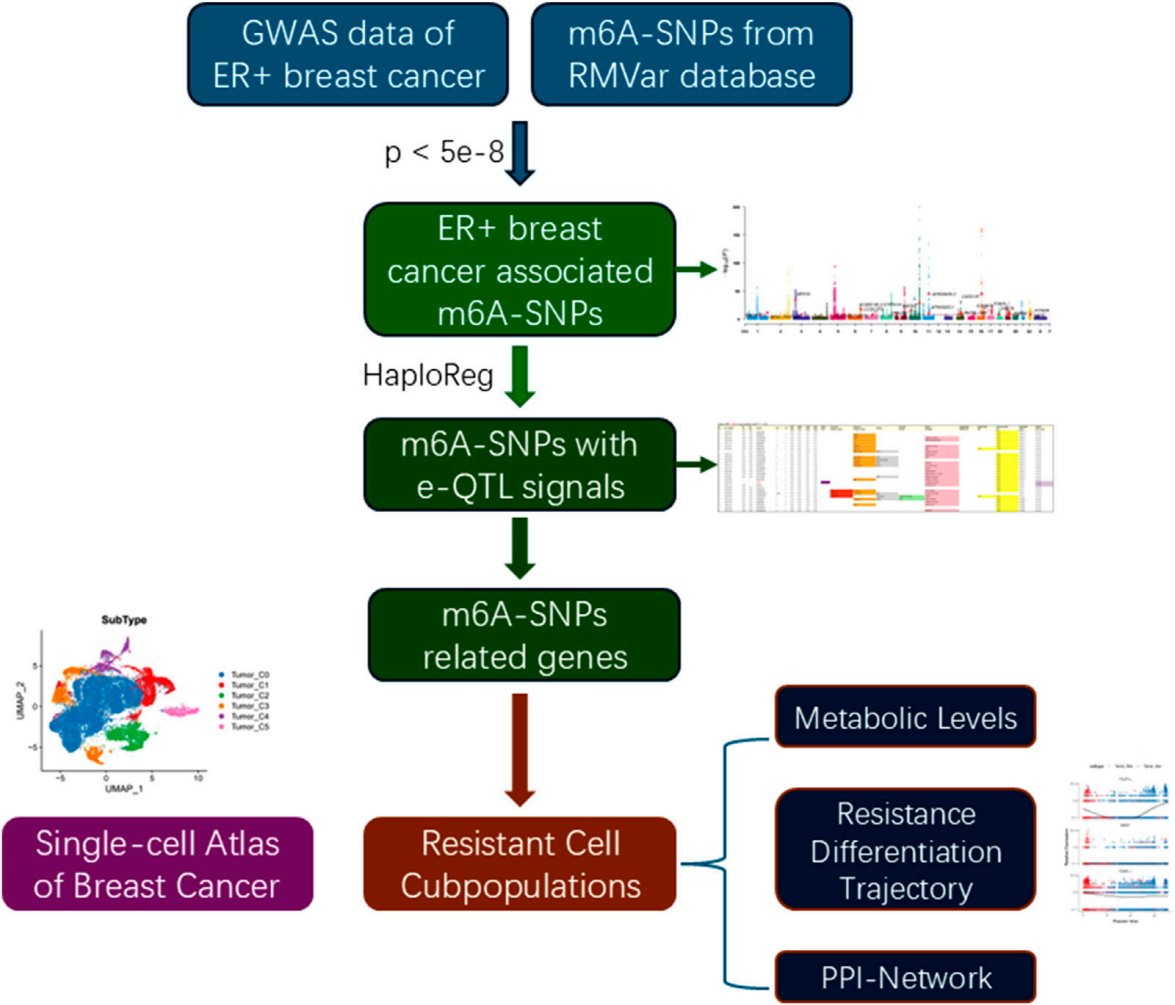
Flowchart of the study.

**FIGURE 2:**
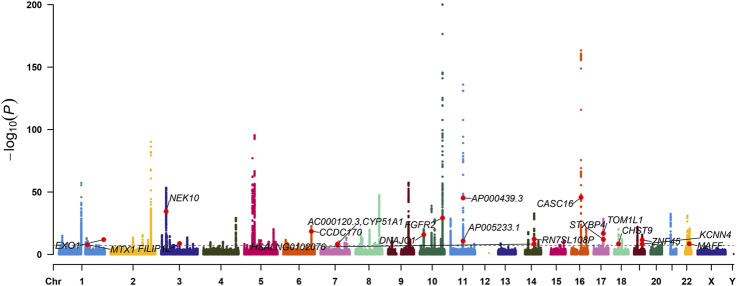
Manhattan plot of ER+ breast cancer-associated m6A-SNPs.

The flowchart was shown in Figure 1. To identify m6A-SNPs associated with breast cancer, we performed an intersection of SNPs from the GWAS and RMVar databases and screened SNPs that met the threshold p < 5e-8. Based on the 10,680,257 SNPs in the ER+ breast cancer GWAS data and 133977 m6A-SNPs in RMVar databases, we identified 24 m6A-SNPs associated with ER+ breast cancer (Figure 2; Supplementary Table S1).

Among the 24 identified m6A-SNPs, 13 exhibited eQTL signals, corresponding to 10 distinct genes. These SNPs include rs4829 in TOM1L1, rs244298 and rs2541243 in STXBP4, rs388685 in ZNF45, rs1017968 in FILIP1L, rs1802212 and rs2267372 and rs9610915 in MAFF, rs2974935 in MTX1, rs2981428 in FGFR2, rs3104793 in CASC16, rs4973758 in NEK10, and rs6465348 in CYP51A1 (Supplementary Figure S1). These findings were summarized in Supplementary Table S2, which detailed the specific eQTL associations and their potential implications in gene regulation by m6A modifications.

### 3.2 Functional enrichment analysis

**FIGURE 3:**
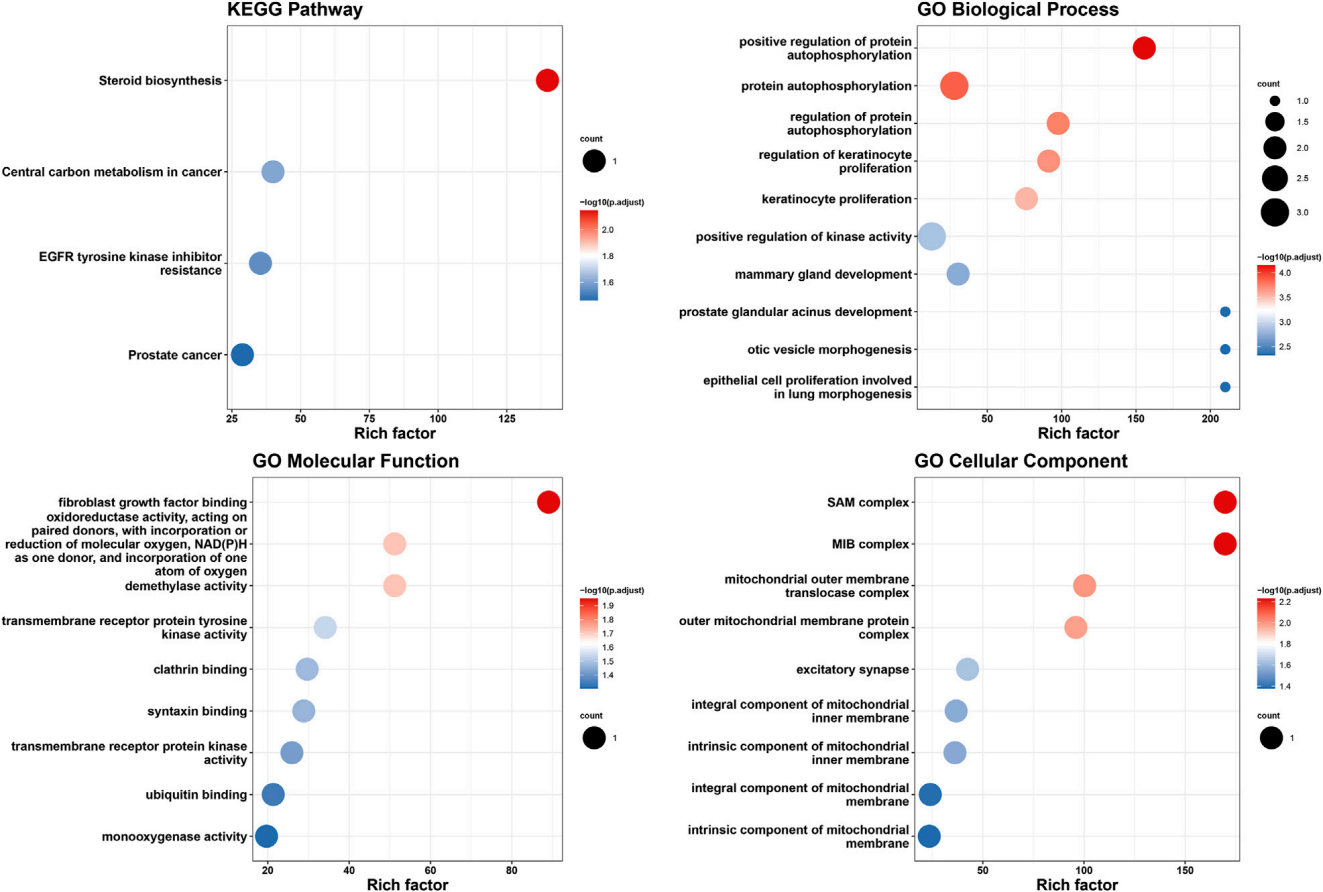
Functional enrichment analysis of genes related to m6A-SNPs associated with ER+ breast cancer.

GO and KEGG pathway enrichment analyses were conducted on the 10 genes associated with the identified m6A-SNPs. The findings indicated significant enrichment in biological functions such as steroid biosynthesis and positive regulation of protein autophosphorylation (Figure 3). These results suggested that m6A-SNPs might modulate biological functions by regulating the expression levels of corresponding genes, particularly impacting pathways like steroid biosynthesis.

### 3.3 Differential expression analysis and prediction of m6A modification sites

**FIGURE 4:**
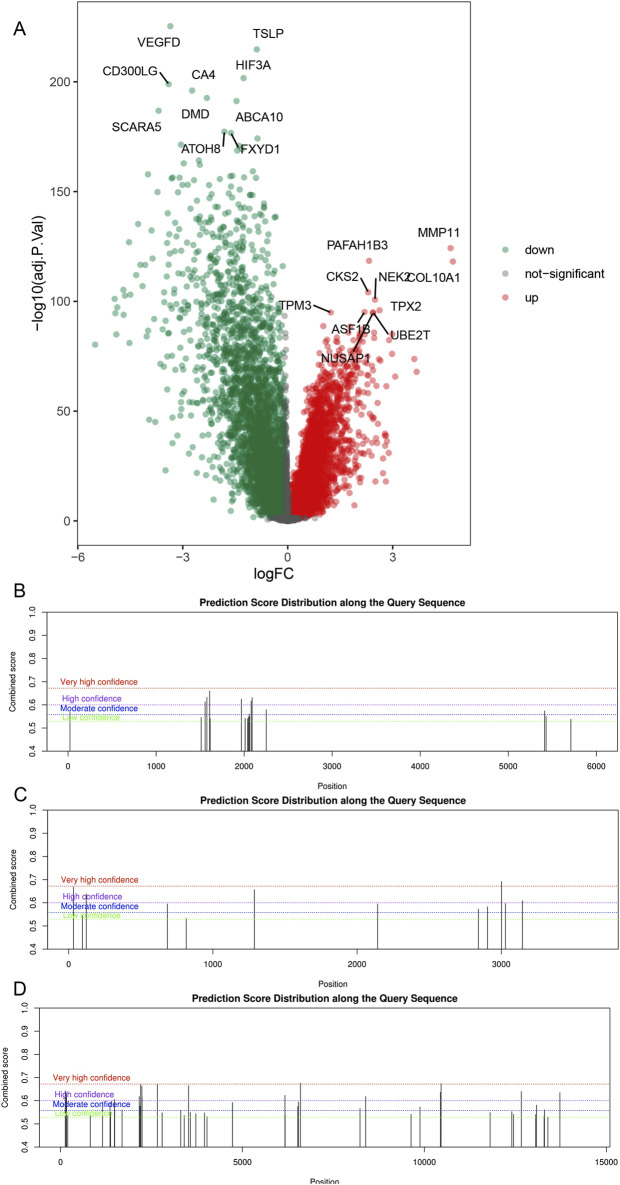
Differential expression and m6A modification sites of m6A-SNPs related genes. Differentially expressed genes between ER+ breast cancer and normal tissues in TCGA-BRCA cohort **(A)**. M6A modifications near the rs1017968 (FILIP1L, **(B)**, rs1802212 and rs4829 (TOM1L1, **(C)**, and rs2267372 and rs9610915 (MAFF, **(D)**. In B-D, the x-axis represents the nucleotide sequence containing the target SNP position, while the y-axis indicates the score assigned by SRAMP for the presence of an m6A peak. In the plot, taller black vertical lines denote a higher predicted probability of m6A modification at that specific sequence position.

In the TCGA-BRCA bulk RNA-seq dataset, we identified differentially expressed genes between ER+ breast cancer samples and normal control samples. Using a threshold of |log2FC| > 0.8 and adjusted p < 0.05, a total of 2,526 differentially expressed genes were identified. Notably, this set includes three genes associated with m6A-SNPs: FILIP1L, TOM1L1, and MAFF (Figure 4A).

Based on the genomic FASTA sequences of the above three differentially expressed m6A-SNPs-related genes, we examined the m6A peak positions in the SRAMP database and compared the m6A peaks to determine whether there was a “Moderate confidence” or “High confidence” m6A peak in the vicinity of the SNPs. If there is, it can be assumed that there is a m6A modification with medium or high confidence near the SNP site. It was found that rs1017968 (FILIP1L), rs1802212 and rs4829 (TOM1L1), and rs2267372 and rs9610915 (MAFF) SNPs did have m6A modifications near the SNP loci (Figures 4B–D).

### 3.4 m6A-SNPs and interaction with RNA-Binding proteins

**FIGURE 5:**
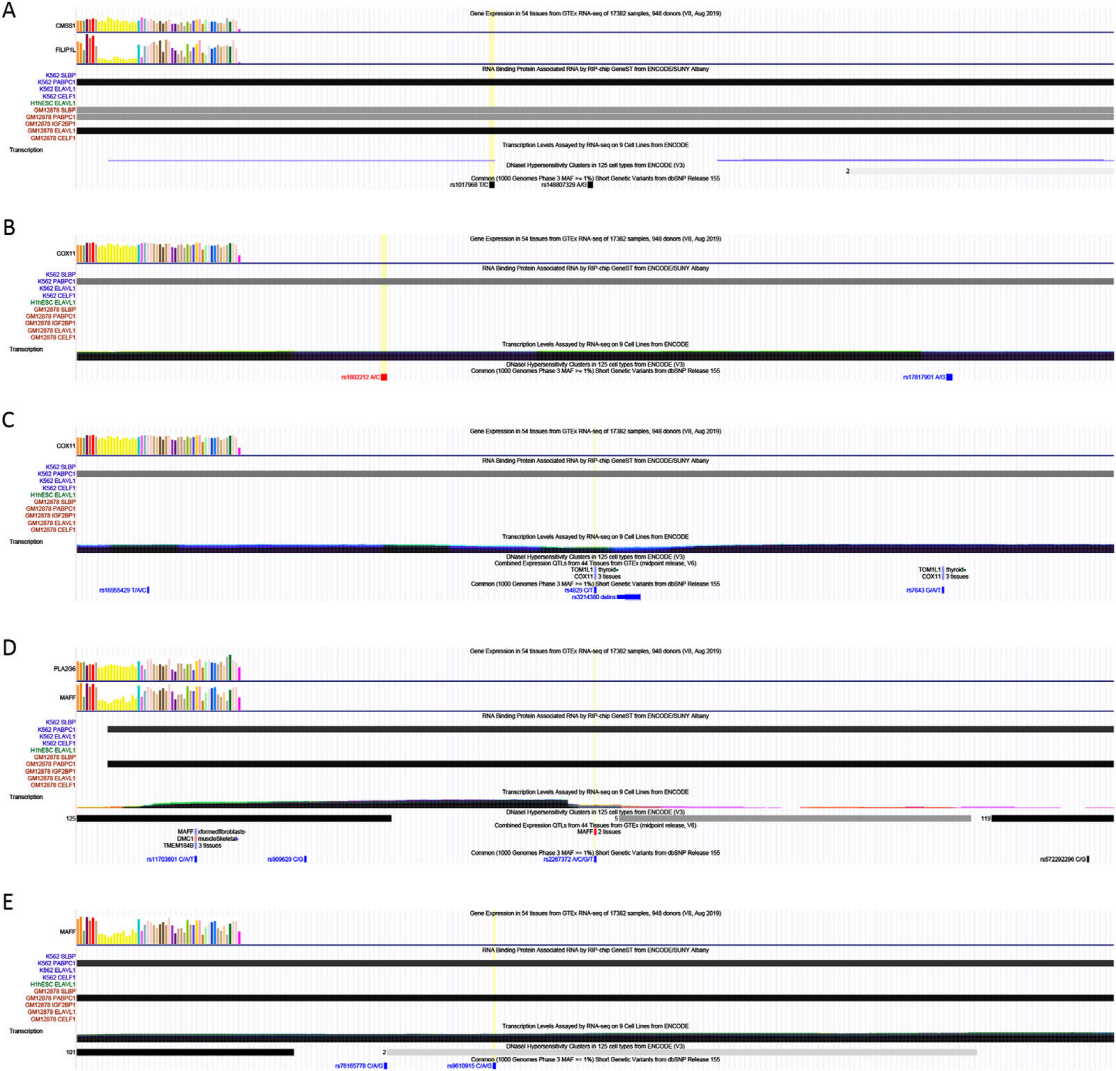
Interaction of m6A-SNPs, rs1017968 **(A)**, rs1802212 **(B)**, rs4829 **(C)**, rs2267372 **(D)** and rs9610915 **(E)**, and RNA-binding proteins. The x-axis represents the genomic coordinates. The y-axis, from top to bottom, displays gene expression profiles across 54 tissues from the GTEx database, the distribution of RNA-binding proteins, transcription levels in various cell lines, DNase I hypersensitive sites, and SNPs located within the genomic region. The yellow vertical line indicates the genomic position of the target SNP.

We utilized the UCSC Genome Browser to analyze the potential roles of m6A-SNPs. As illustrated in Figure, rs1017968 is located in the intronic region of the FILIP1L gene on chromosome three and exhibits potential interactions with RNA-binding proteins such as ELAVL1 and PABPC1 (Figure 5A). Similarly, rs1802212 and rs4829 are located in the 3′UTR of the TOM1L1 gene on chromosome 17 and show potential interactions with PABPC1 (Figures 5B,C). Additionally, rs2267372 and rs9610915, situated in the exon and 3′UTR regions of the MAFF gene on chromosome 22, also potentially interact with RNA-binding proteins including PABPC1 (Figures 5D,E).

### 3.5 Identification of resistant cell subpopulations

**FIGURE 6:**
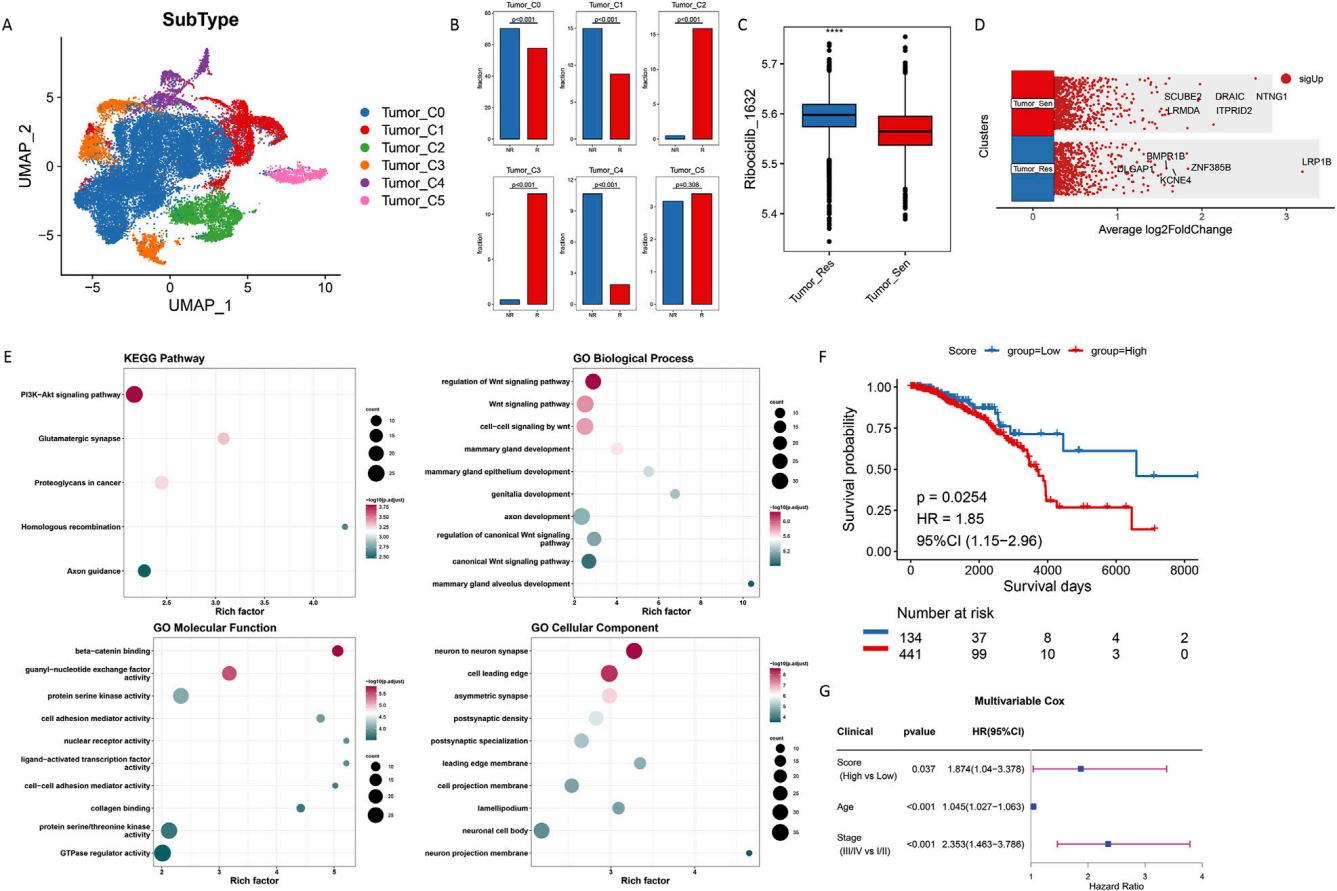
Identification and characterization of resistant subpopulations in tumor cells. **(A)** UMAP Clustering of Tumor Cell Subpopulations. **(B)** Distribution of Resistant and Non-resistant Cells in Clusters. **(C)** IC50 Values for Ribociclib across Tumor Subpopulations. **(D)** Differential Gene Expression in Tumor_Resistant Cells. **(E)** Pathway Analysis of Upregulated Genes. **(F)** Kaplan-Meier Survival Curves for Resistant and Sensitive Subgroups. **(G)** Multivariable Cox regression analysis of overall survival was performed based on three factors: the score (High vs Low), age, and clinical stage (III/IV vs I/II).

In this study, we analyzed 28,495 tumor cells, including 18,948 cells in the Responders (R) group and 9,547 cells in the non-responders (NR) group. A notable batch effect was identified within these 12 samples and subsequently removed through the harmony package before re-clustering, as illustrated by the comparison in Supplementary Figure S2. We clustered these tumor cells and stratified them into subpopulations at a resolution of 0.05, resulting in six distinct cell clusters (Figure 6A). Based on the proportion of NR cells within each cluster, clusters 0, 1, and 4 exhibited a significantly higher proportion of NR cells, while clusters 2 and 3 had a significantly higher proportion of R cells (Figure 6B). Consequently, we defined clusters 0, 1, and 4 as Tumor_Resistant (Tumor_Res), clusters 2 and 3 as Tumor_Sensitive (Tumor_Sen), and cluster 5, which showed no significant differences between NR and R proportions, as Mixed and excluded it from further analysis.

After calculating the IC50 of ribociclib in tumor cells, we found that the IC50 values were significantly higher in the Tumor_Res cells, indicating a lower sensitivity to ribociclib (Figure 6C). Differential analysis revealed significant upregulation of genes such as LRP1B and ZNF385B in the Tumor_Res group (Figure 6D). Functional analysis showed that these genes play crucial roles in regulatory pathways such as the PI3K-Akt signaling pathway and the Wnt signaling pathway (Figure 6E).

Gene Set Variation Analysis (GSVA) was employed to assess the differential expression scores of genes associated with the resistant subgroups across individual samples in the TCGA-BRCA dataset. Based on median values, samples were stratified into groups to evaluate the prognostic impact using Kaplan-Meier survival curves. These analyses indicate that the resistant subgroups are associated with poor patient outcomes (Figure 6F). In the multivariate Cox regression analysis incorporating score, clinical stage, and age, a high score was also significantly associated with poor prognosis in patients with ER+ breast cancer (Figure 6G). These Results highlighted the potential prognostic significance of these gene expression patterns in ER+ breast cancer.

### 3.6 Metabolic profiling of cell subpopulations

**FIGURE 7:**
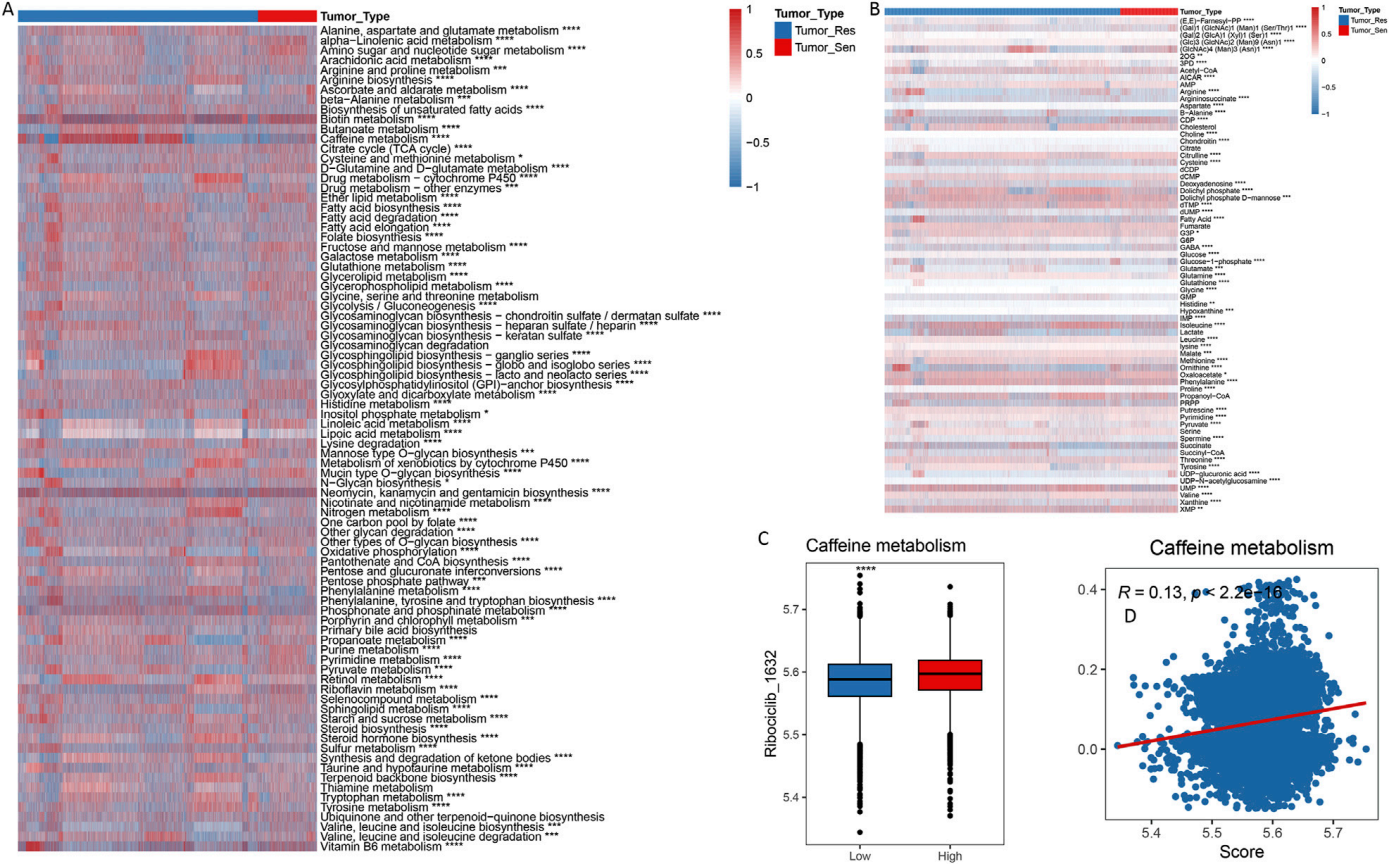
Metabolic features of cell subpopulations. Metabolic profile differences between Tumor_Resistant and Tumor_Sensitive groups through GSVA **(A)** and scFEA **(B)**. **(C,D)** Correlation Between Ribociclib IC50 and Caffeine Metabolism.

We utilized Gene Set Variation Analysis (GSVA) to evaluate the metabolic characteristics of tumor cells. As depicted in Figure 7A, there were significant differences in metabolic features between Tumor_Resistant (Tumor_Res) and Tumor_Sensitive (Tumor_Sen) groups. Notably, caffeine metabolism activity was significantly enhanced in the Tumor_Res group. Additionally, scFEA (single-cell Flux Estimation Algorithm) was used to calculate the abundance of metabolites in the tumor cell subpopulations, revealing a significant increase in UMP and phenylalanine levels in the Tumor_Res group (Figure 7B). The results showed that metabolic pathways such as folate biosynthesis, caffeine metabolism, and ether lipid metabolism were upregulated in Tumor_Res group, suggesting that these elevated pathways may contribute to CDK4/6 inhibitor resistance. In addition, increased levels of metabolites—including lysine, acetyl-CoA, and glutamine—were detected in Tumor_Res group, indicating that their enrichment may further promote resistance development (Figures 7A,B). Notably, methionine, known as m6A-related metabolite, was significantly downregulated in Tumor_Res group, suggesting that aberrant m6A modifications might play a critical role in the development of resistance to CDK4/6 inhibitor (Figure 7B). Furthermore, there was a significant positive correlation between the IC50 of the drug ribociclib and caffeine metabolism (Figures 7C,D), suggesting that altered metabolic pathways may influence drug sensitivity in these cells.

### 3.7 Pseudo-temporal analysis of the Tumor_Res cell cluster

**FIGURE 8:**
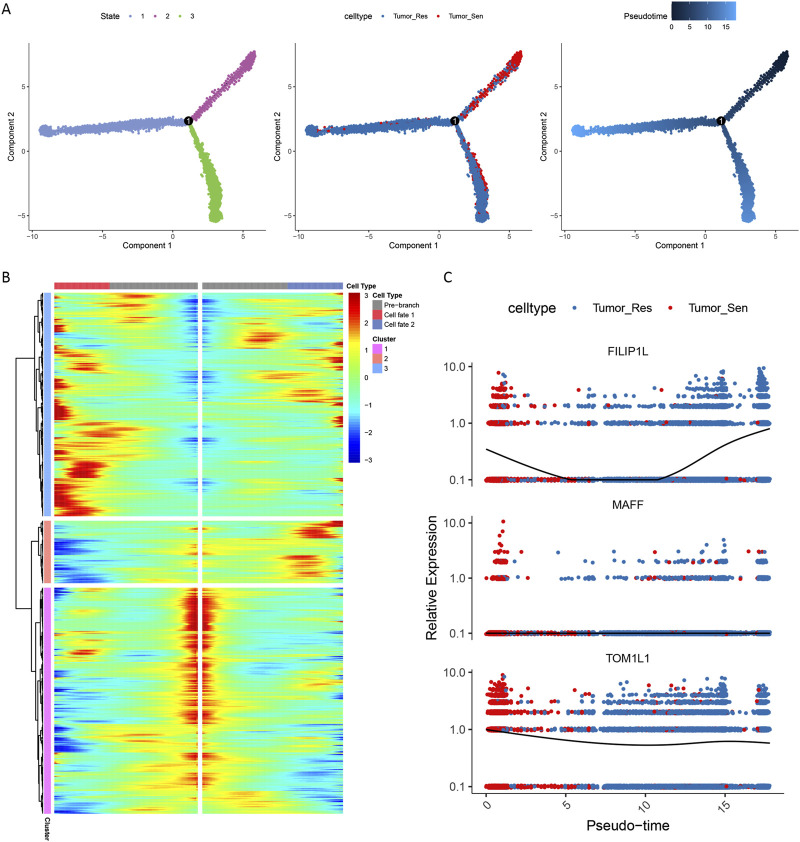
Pseudotime trajectory analysis of tumor cell differentiation. **(A)** Developmental trajectory of tumor cells. **(B)** BEAM analysis at node 1. **(C)** Expression patterns of FILIP1L, MAFF and TOM1L1.

To explore the transformation process from sensitive to resistant tumor cells, we constructed a pseudo-temporal developmental trajectory of tumor cells using single-cell data. The tumor cells were divided into three developmental states, with resistant tumor cells occupying the trajectory’s endpoint, exhibiting a highly differentiated state (Figure 8A). Further analysis indicated that tumor cells underwent a round of cell fate selection, with a subset ultimately differentiating into resistant cells (Figure 8A).

Node 1 represents a critical point in the differentiation trajectory where tumor cells transition from sensitive to resistant, prompting us to perform BEAM analysis at this node to explore genes exhibiting branch-dependent expression. During the differentiation process, genes in cluster 3 were highly expressed in cell fate1, while genes in cluster 2 were highly expressed in cell fate2 (Figure 8B). During the differentiation from sensitive to resistant cells, the expression levels of the m6A-SNP-related gene FILIP1L initially decreased and then increased, whereas another m6A-SNP-related gene, TOM1L1, showed a decrease in expression as cells became resistant. This suggests that FILIP1L and TOM1L1 may be part of the mechanism of driving resistance (Figure 8C).

**FIGURE 9:**
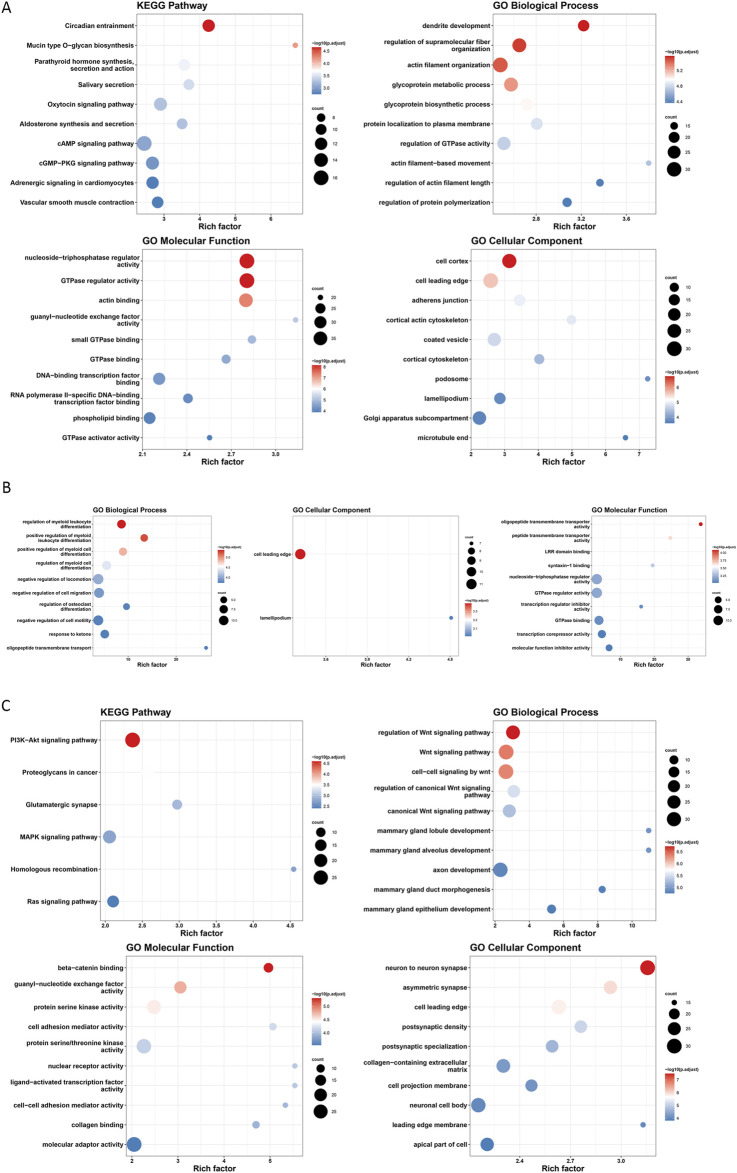
Gene enrichment analysis in cell fate clusters. Functional enrichment in cluster 1 **(A)**, cluster 2 **(B)** and cluster 3 **(C)**.

An enrichment analysis of the genes involved in each cluster revealed that genes in cluster1 were associated with functions like circadian entrainment and regulation of GTPase activity (Figure 9A). Genes in cluster2 played roles in functions such as oligopeptide transmembrane transporter activity (Figure 9B). Meanwhile, genes in cluster3 were crucial in regulating the PI3K-Akt and Wnt signaling pathways (Figure 9C).

### 3.8 Analysis of resistant subgroup characteristic genes and m6A-SNPs related genes

**FIGURE 10:**
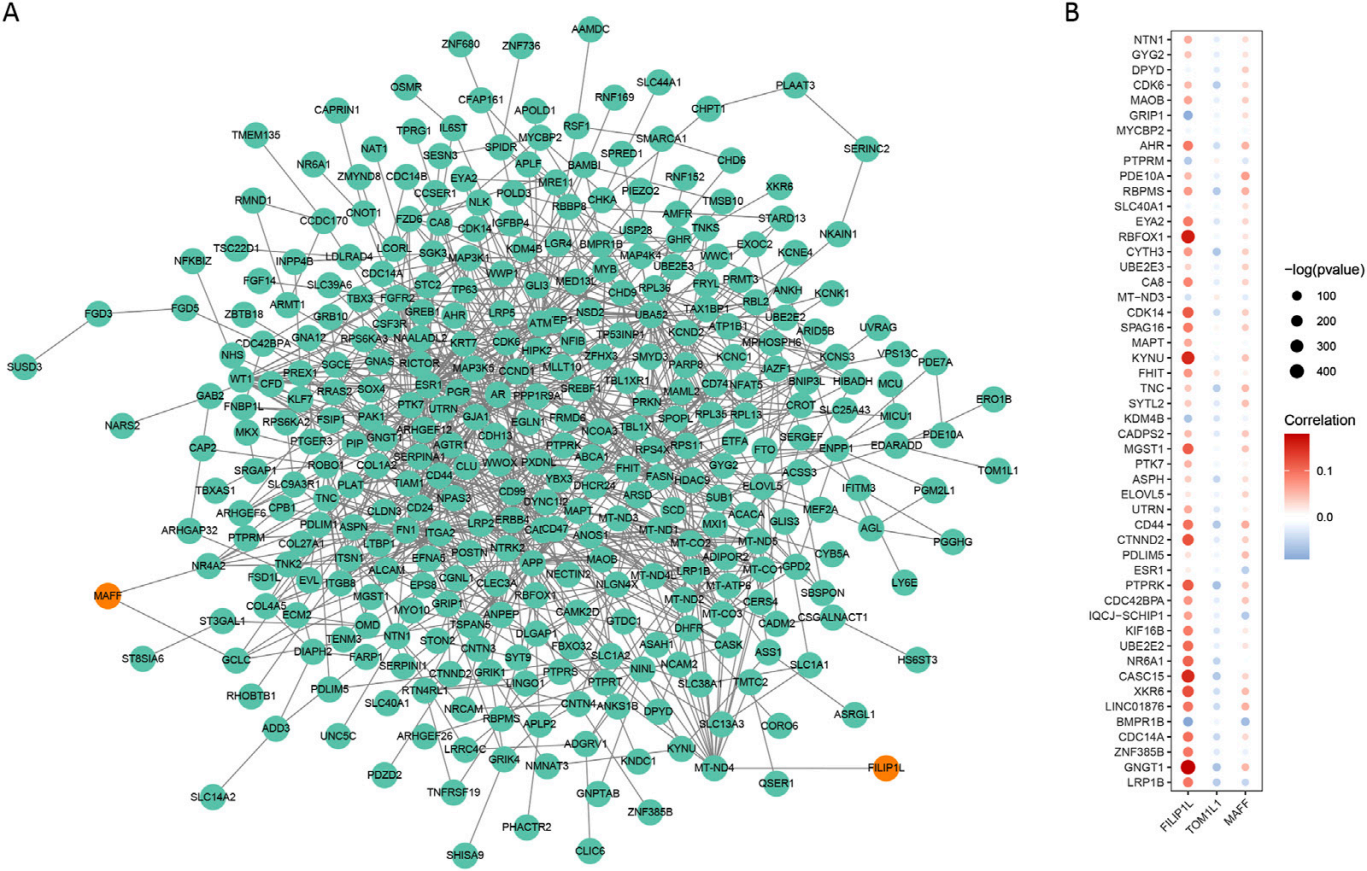
Analysis of Protein-Protein Interactions and Correlations Between Resistant Subgroup Characteristic Genes and m6A-SNPs Related Genes. **(A)** Protein-Protein Interaction Network. **(B)** Correlation Analysis of m6A-SNPs Related and Resistant Subgroup Characteristic Genes.

To further investigate the association between resistant subgroup characteristic genes and m6A-SNPs related genes, we conducted protein-protein interaction (PPI) and correlation analyses. Utilizing the String database, we explored the interaction network between characteristic genes of the resistant subgroup and m6A-SNPs related genes. Notably, the m6A-SNP related gene FILIP1L interacts with the resistant subgroup characteristic gene MT-ND4, and MAFF interacts with NR4A2 and GCLC within the resistant subgroup (Figure 10A).

Correlation analysis between m6A-SNPs related genes and the characteristic genes of the resistant subgroup revealed that FILIP1L shows a significant positive correlation with GNGT1 and RBFOX1, and a significant negative correlation with BMPR1B (Figure 10B). These findings suggested that FILIP1L and TOM1L1 might serve as potential biomarkers for resistance.

## 4 Discussion

Resistance to endocrine therapy in patients with ER+ breast cancer represents a significant clinical challenge, often leading to treatment failure and poor prognostic outcomes (Portman et al., 2019; Ferro et al., 2024). The role of m6A modification has been increasingly recognized in ER+ breast cancer, influencing RNA stability and gene expression that are pivotal in cancer progression and response to therapy (Yang et al., 2024).

Our study approached this problem by integrating GWAS data with scRNA-seq analyses to identify m6A-SNPs that potentially contribute to endocrine therapy resistance. We pinpointed significant SNPs and their association with gene expression changes in tumor cells, focusing on their variability between sensitive and resistant cell populations. Our findings highlight the roles of specific m6A-SNPs related genes, particularly FILIP1L and TOM1L1, which were found involved in key pathways influencing therapy resistance. Additionally, our study elucidated the diverse expression patterns within tumor cell subpopulations, offering insights into the heterogeneity of response to CDK4/6 inhibitors.

Although there still no studies revealed the roles of FILIP1L and TOM1L1 in m6A modification, their functions in cancer have been preliminarily described. FILIP1L is known for its proven ability to inhibit the biological functions of a wide range of tumor cells and has the potential to be a therapeutic target for cancer (Kwon and Libutti, 2014). It can inhibit the formation of chemoresistance in tumor cells by suppressing the Wnt signaling pathway (Kwon et al., 2016). Similarly, upregulated FILIP1L inhibited metastasis of triple-negative breast cancer cells (Jiang et al., 2024). Moreover, the upregulated FILIP1L was also able to increase the sensitivity of breast cancer cells to Topoisomerase II (TOP2) targeting drugs (Lu and Hallstrom, 2012). Previous studies have identified TOM1L1 as a gene associated with m6A-SNPs in breast cancer (Xuan et al., 2021). And TOM1L1 was able to promote ERBB2-induced breast cancer cell invasion by driving membrane delivery of membrane-type 1 matrix metalloprotease (MT1-MMP) (Chevalier et al., 2016a; Chevalier et al., 2016b). The mechanisms by which these two genes regulate m6A modifications that affect drug resistance in breast cancer have not yet been revealed, and more in-depth studies are urgently needed to disclosure.

In ER+ breast cancer, the modulation of the PI3K-Akt and Wnt signaling pathways by m6A-SNPs related genes like FILIP1L and TOM1L1 is particularly compelling, given the established role of these pathways in promoting estrogen receptor signaling and cellular proliferation (Alves and Ditzel, 2023). Our observations are consistent with literature that connects dysregulated m6A landscapes with altered signaling pathways, which may enhance tumor aggressiveness and resistance to endocrine therapy (Tabnak et al., 2023). Thus, targeting specific m6A modifications offers a promising strategy to modulate these critical pathways and improve therapeutic outcomes. Recent findings have highlighted the role of N6-methyladenosine (m6A) modification in ER+ breast cancer, particularly its capacity to regulate mRNA dynamics, such as the expression of CDK6, a critical mediator in cell cycle progression and a known target of CDK4/6 inhibitors (Xia et al., 2024).

Recent evidence has further illuminated the interplay between m6A regulatory enzymes and critical drivers of therapy response in ERα-positive breast cancer. For example, METTL3-mediated m6A methylation has been shown to stabilize ESR1 transcripts, thereby reinforcing ESR1 activity as a key transcription factor (Zhou et al., 2025). Such a METTL3–ESR1 loop can potentially sustain estrogen-driven signaling under therapeutic pressure. Additionally, inhibition of METTL14 has been reported to overcome CDK4/6 inhibitor resistance by disrupting the METTL14–m6A–E2F1 axis in ERα-positive cells (Liu et al., 2025). These findings imply that m6A modifications may act in parallel with, or even converge upon, established drivers of endocrine therapy resistance, including ESR1 mutations and dysregulated cell-cycle regulators (e.g., CDK4/6). Future studies integrating high-throughput m6A mapping, gene mutation profiling, and functional assays will be critical for clarifying whether targeting FILIP1L or TOM1L1 could synergistically restore CDK4/6 inhibitor sensitivity. Elucidating these overlapping mechanisms stands to refine combination regimens against therapy-resistant ER+ breast cancer.

Recent studies suggest that other RNA modifications, such as 5-methylcytosine (m5C) and 1-methyladenosine (m1A), may likewise influence progression and therapy resistance in various malignancies (Wang et al., 2021; Chen et al., 2024). Aberrant m5C modifications have been reported to impair RNA stability and alter gene expression, leading to metabolic reprogramming and ferroptosis, ultimately facilitating tumor resistance to therapy (Hou et al., 2025; Shi et al., 2025). Meanwhile, aberrant m1A modifications can alter mRNA structure or translation efficiency, potentially driving tumor cells to evade therapeutic pressure (Li et al., 2024; Modi et al., 2024). Incorporating these emerging insights into the epitranscriptomic landscape of ER+ breast cancer will be critical for pinpointing novel targets and devising more effective, combination-based treatments aimed at overcoming resistance.

Emerging evidence highlights the critical role of STAT3 signaling in mediating CDK4/6 inhibitors resistance in several cancers including breast cancer. Persistent or dysregulated STAT3 activation has been shown to drive tumor cell proliferation, apoptosis evasion, and immune modulation, thereby promoting resistance to CDK4/6 inhibitors (Dong et al., 2024; Wu et al., 2024). Notably, STAT3 synergizes with PI3K-Akt and Wnt pathways—central to our findings—to bypass therapy. For example, STAT3 elevates cyclin D1 expression, circumventing CDK4/6 inhibition, and stabilizes MCL1 to suppress apoptosis (Osaki et al., 2022; Wang X. et al., 2023; Yin et al., 2024). Furthermore, STAT3 modulates RNA metabolism, including m6A dynamics, by regulating RNA-binding proteins like METTL3, which may influence mRNA stability of resistance genes (Liu et al., 2023). These insights align with our observed pathway dysregulation and suggest combinatorial targeting of STAT3 and m6A modifiers as a resistance-countering strategy.

While our findings were promising, they were not without limitations. The reliance on bioinformatic predictions and single-cell RNA-seq data may introduce biases and limit the physiological interpretation of m6A-SNPs' roles without direct experimental validation. Furthermore, the complexity of m6A regulatory mechanisms and their context-dependent effects demand more comprehensive *in vivo* studies to fully understand their impact on breast cancer pathology and treatment outcomes. Additionally, our analysis was based on GWAS data derived solely from individuals of European ancestry, reflecting the current lack of large-scale datasets from other populations, such as Asian cohorts. Given that allele frequencies and regulatory effects of m6A-SNPs may vary across ethnic backgrounds, the generalizability of our findings may be limited. Future studies incorporating ethnically diverse GWAS and epitranscriptomic data will be essential to validate these associations and support broader clinical translation. Furthermore, integrating longitudinal clinical data and functional assays to validate the influence of identified m6A-SNPs on therapy resistance and patient outcomes.

## 5 Conclusion

This study confirms the role of m6A-SNPs in influencing endocrine therapy resistance in ER+ breast cancer, highlighting how modifications mediated by specific SNPs, particularly in FILIP1L and TOM1L1, impact key regulatory pathways and cellular metabolism. Our findings emphasize the potential of targeting m6A-related mechanisms to improve therapeutic strategies and overcome resistance, suggesting a direction for future research to enhance the precision of breast cancer treatment.

## Data Availability

Publicly available datasets were analyzed in this study. This data can be found here: The GWAS summary statistics for ER+ breast cancer were sourced from the IEU database (https://gwas.mrcieu.ac.uk/datasets/ieu-a-1127/). M6A-SNPs were from RMVar database (https://www.rmvar.renlab.org/). Single-cell data for ER+ breast cancer treated with CDK4/6 inhibitors combined with endocrine therapy were sourced from GSE158724 (https://www.ncbi.nlm.nih.gov/geo/query/acc.cgi?acc=GSE158724).

## Supplementary material

SUPPLEMENTARY FIGURE S1 Among the identified m6A-SNPs, 13 exhibited eQTL signals, collectively corresponding to 10 distinct genes.

SUPPLEMENTARY FIGURE S2 Comparison of tumor cell clustering before and after batch effect removal. **(A)** UMAP embedding without batch correction. **(B)** UMAP embedding following the Harmony algorithm.
