# Supplementary figures and images for: Integrative analysis of m6A-SNPs and single-cell RNA sequencing reveals key drivers of endocrine combined with CDK4/6 inhibitor therapy resistance in ER+ breast cancer
Source: Front Pharmacol. 2025 Apr 15;16:1590363. doi: 10.3389/fphar.2025.1590363 (PMC12038269; doi:10.3389/fphar.2025.1590363)

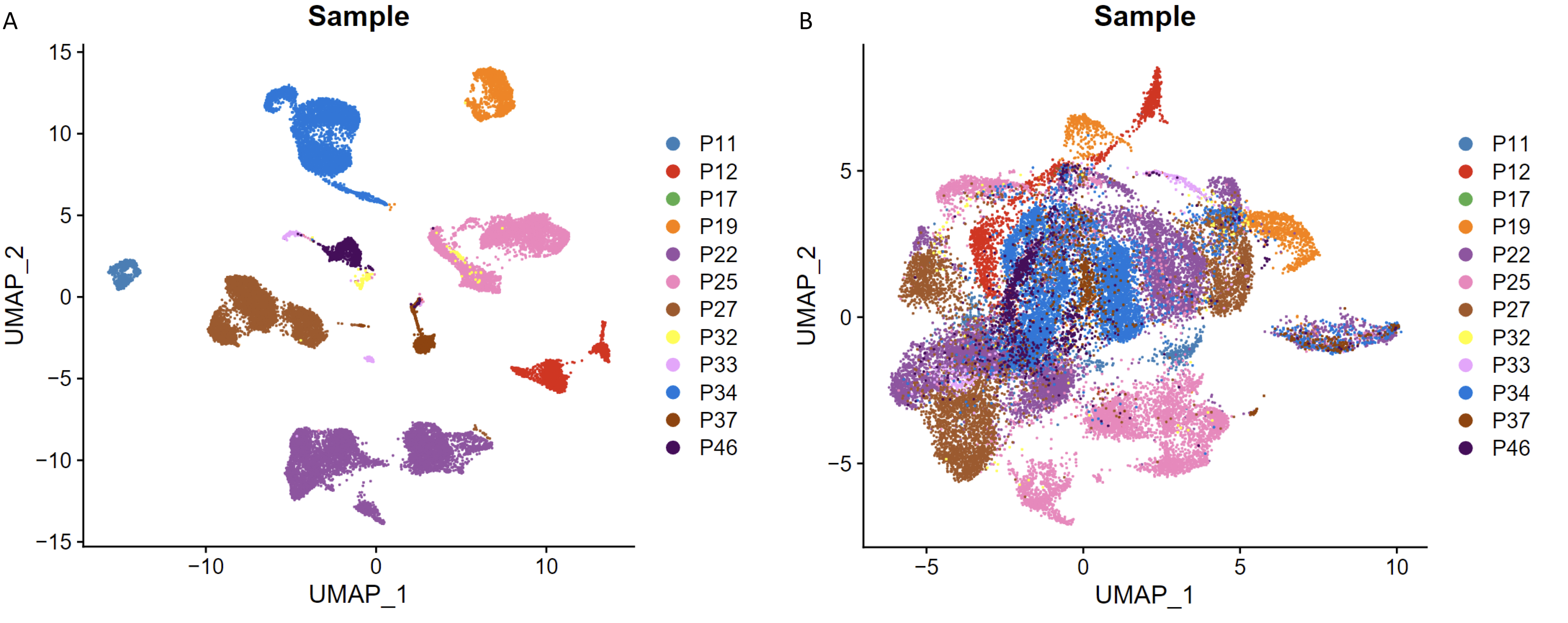

Supplement: Supplementary file 1 [file Image2.tif]
