# Supplementary material for: Integrative analysis of m6A-SNPs and single-cell RNA sequencing reveals key drivers of endocrine combined with CDK4/6 inhibitor therapy resistance in ER+ breast cancer
Source: Front Pharmacol. 2025 Apr 15;16:1590363. doi: 10.3389/fphar.2025.1590363 (PMC12038269; doi:10.3389/fphar.2025.1590363)
Supplement: Supplementary file 3 [file Image1.pdf]

Query SNP: rs4829 and variants with $r^2 >= 0.8$

chr	pos (hg38)	LD (r^2)	LD (D')	variant	Ref	Alt	AFR freq	AMR freq	ASN freq	EUR freq	SiPhy cons	Promoter histone marks	Enhancer histone marks	DNAse	Proteins bound	Motifs changed	NHGRI/EBI GWAS hits	GRASP QTL hits	Selected eQTL hits	GENCODE genes	dbSNP func annot
17	54924095	0.81	0.96	rs9303360	T	C	0.62	0.47	0.32	0.58									6 hits	TOM1L1	intronic
17	54926783	0.81	0.94	rs6504947	T	A	0.79	0.48	0.37	0.56			ESDR						4 hits	TOM1L1	intronic
17	54928225	0.88	0.99	rs12952166	C	T	0.79	0.47	0.37	0.57			ESDR			4 altered motifs			4 hits	TOM1L1	intronic
17	54930294	0.89	0.99	rs12939352	T	G	0.54	0.45	0.32	0.57			GI			HNF4,RXRA,ZBTB7A			3 hits	TOM1L1	intronic
17	54931512	0.89	0.99	rs8069447	T	G	0.54	0.45	0.32	0.57			GI						3 hits	TOM1L1	intronic
17	54933811	0.89	0.99	rs4794547	A	G	0.54	0.45	0.31	0.57			ESDR, GI			6 altered motifs			3 hits	TOM1L1	intronic
17	54935018	0.89	0.99	rs9901526	G	T	0.62	0.46	0.32	0.57			ESC, GI			HNF1		1 hit	7 hits	TOM1L1	intronic
		0.8	0.91	rs79594112	C	T	0.75	0.44	0.36	0.53			BRN, MUS	MUS		4 altered motifs				TOM1L1	intronic
17	54942351	0.97	0.99	rs7503269	T	C	0.62	0.44	0.21	0.54						LXR,Myc,Sp4			6 hits	TOM1L1	intronic
17	54944594	0.99	0.99	rs11079142	A	G	0.42	0.42	0.21	0.54			BLD, MUS	BLD		Tgif1			5 hits	TOM1L1	intronic
17	54944688	0.99	0.99	rs12165058	T	C	0.44	0.42	0.21	0.54			4 tissues	MUS,MUS,BLD					6 hits	TOM1L1	intronic
17	54945353	0.99	0.99	rs12945579	A	T	0.44	0.42	0.21	0.54			5 tissues	BLD		ZBTB7A			6 hits	TOM1L1	intronic
17	54948119	0.99	0.99	rs4793783	T	C	0.44	0.42	0.21	0.54			11 tissues	MUS		Pax-4,Prrx2,Sox			6 hits	TOM1L1	intronic
17	54952253	0.99	0.99	rs12944690	A	G	0.42	0.42	0.21	0.54						Pou5f1			5 hits	TOM1L1	intronic
17	54955646	0.98	0.99	rs2332314	T	G	0.44	0.42	0.21	0.54						Arid5b,BRCA1			6 hits	TOM1L1	intronic
17	54958241	0.99	0.99	rs12951898	C	T	0.42	0.42	0.21	0.54						INSM1,MZF1::1-4,TAL1			5 hits	TOM1L1	intronic
17	54958667	0.99	0.99	rs2111096	C	T	0.44	0.42	0.21	0.54				IPSC		ERalpha-a,Zbtb3			6 hits	TOM1L1	intronic
17	54961069	1	1	rs4793784	A	G	0.42	0.42	0.21	0.54			BLD			6 altered motifs			5 hits	TOM1L1	intronic
17	54961954	1	1	rs4829	C	T	0.42	0.42	0.21	0.54						11 altered motifs			5 hits	COX11	3'-UTR
17	54962122	0.99	1	rs7643	G	A	0.41	0.42	0.21	0.54						Ets,SIX5,TCF4			5 hits	COX11	3'-UTR
17	54964496	0.99	0.99	rs3213757	A	G	0.41	0.42	0.21	0.54									5 hits	COX11	intronic
17	54965689	0.98	0.99	rs34096591	T	C	0.41	0.43	0.21	0.54		BLD	4 tissues			CACD,SREBP			5 hits	COX11	intronic
17	54967022	0.98	0.99	rs140861493	AAAT	A	0.40	0.42	0.19	0.54		15 tissues	15 tissues	VAS		25 altered motifs				COX11	intronic
17	54967882	0.95	0.99	rs12945393	G	A	0.41	0.43	0.28	0.55		23 tissues		39 tissues	FOXA1,POL2,TBP	CEBPB		1 hit	6 hits	COX11	intronic
17	54972664	0.81	0.97	rs1990673	C	T	0.79	0.48	0.44	0.58			4 tissues	PANC	PU1	AIRE,GR,KAP1			4 hits	STXBP4	intronic
17	54980354	0.83	0.99	rs9898020	G	A	0.79	0.48	0.44	0.58						7 altered motifs			4 hits	STXBP4	intronic
17	54985192	0.82	0.98	rs9303362	G	A	0.80	0.48	0.44	0.58			MUS			Hdx,Pou5f1			4 hits	STXBP4	intronic
17	54989248	0.91	0.99	rs11869372	T	C	0.66	0.44	0.27	0.56									8 hits	STXBP4	intronic
17	54991569	0.88	0.96	rs12940239	A	G	0.66	0.44	0.28	0.55						Hmbox1			7 hits	STXBP4	intronic

Query SNP: rs244298 and variants with r^2 >= 0.8

chr	pos (hg38)	LD (r^2)	LD (D')	variant	Ref	Alt	AFR freq	AMR freq	ASN freq	EUR freq	SiPhy cons	Promoter histone marks	Enhancer histone marks	DNAse	Proteins bound	Motifs changed	NHGRI/EBI GWAS hits	GRASP QTL hits	Selected eQTL hits	GENCODE genes	dbSNP func annot
17	55092522	0.84	-0.95	rs11655949	A	G	0.24	0.52	0.56	0.43			BLD			Cphx			1 hit	STXBP4	intronic
17	55094485	0.84	-0.95	rs12453699	C	A	0.21	0.52	0.57	0.43			ESDR, BLD			PLZF			2 hits	STXBP4	intronic
17	55094748	0.84	-0.95	rs12453779	G	A	0.24	0.52	0.57	0.43			ESDR, BLD			Bcl6b,Homez,Maf			1 hit	STXBP4	intronic
17	55095607	0.84	-0.95	rs7207057	G	C	0.24	0.52	0.57	0.43			ESDR			Arid5a			1 hit	STXBP4	intronic
17	55095685	0.84	-0.95	rs7209029	A	G	0.24	0.52	0.57	0.43			ESDR, BLD			GR			1 hit	STXBP4	intronic
17	55097073	0.84	-0.95	rs8072090	A	T	0.28	0.53	0.56	0.43						NR4A,VDR			1 hit	STXBP4	intronic
17	55097906	0.86	-0.97	rs8080876	T	C	0.29	0.52	0.56	0.43						SIX5		1 hit	2 hits	STXBP4	intronic
17	55098662	0.91	-0.97	rs11079153	C	G	0.28	0.52	0.56	0.42						AFP1,Ets			1 hit	STXBP4	intronic
17	55099178	0.92	-0.97	rs7224560	G	C	0.28	0.52	0.56	0.42						Foxf1,Foxj1,Foxo			1 hit	STXBP4	intronic
17	55100148	0.91	-0.97	rs12453615	C	T	0.24	0.52	0.56	0.42						DMRT1				STXBP4	intronic
17	55100538	0.92	-0.97	rs8068337	T	C	0.21	0.52	0.56	0.42										STXBP4	intronic
17	55100922	0.92	-0.97	rs2628326	T	C	0.24	0.52	0.56	0.42				IPSC		Irf,NRSF				STXBP4	intronic
17	55100980	0.92	-0.97	rs2787504	A	G	0.24	0.52	0.56	0.42						Evi-1,Osf2				STXBP4	intronic
17	55103911	0.92	-0.97	rs2529507	C	T	0.28	0.52	0.56	0.42									1 hit	STXBP4	intronic
17	55104185	0.92	-0.97	rs2787470	G	A	0.28	0.52	0.56	0.42						KAP1,Smad			1 hit	STXBP4	intronic
17	55104605	0.89	-0.96	rs2529509	T	C	0.28	0.51	0.55	0.42						AP-1			1 hit	STXBP4	intronic
17	55105065	0.9	-0.96	rs189676	A	G	0.22	0.51	0.56	0.42						BCL,Obox3,STAT				STXBP4	intronic
17	55105085	0.88	-0.95	rs189675	G	A	0.24	0.51	0.55	0.42						4 altered motifs				STXBP4	intronic
17	55105471	0.89	-0.95	rs201079550	T	TTC	0.21	0.51	0.56	0.41						7 altered motifs			1 hit	STXBP4	intronic
17	55106393	0.92	-0.97	rs244374	A	G	0.28	0.52	0.56	0.42				BLD		4 altered motifs			1 hit	STXBP4	intronic
17	55107851	0.92	-0.97	rs244372	G	C	0.29	0.52	0.56	0.42			IPSC			6 altered motifs			1 hit	STXBP4	intronic
17	55107864	0.92	-0.97	rs244371	A	G	0.28	0.52	0.56	0.42			IPSC			8 altered motifs			1 hit	STXBP4	intronic
17	55107906	0.92	-0.97	rs244370	G	A	0.28	0.52	0.56	0.42			IPSC			Nkx2,Nr2f2,Pax-5			1 hit	STXBP4	intronic
17	55108442	0.92	-0.97	rs181728	T	C	0.28	0.52	0.56	0.42						6 altered motifs			1 hit	STXBP4	intronic
17	55108473	0.9	-0.97	rs171514	A	G	0.28	0.52	0.56	0.42						AP-4			1 hit	STXBP4	intronic
17	55108700	0.92	-0.97	rs244369	C	G	0.28	0.52	0.56	0.42						4 altered motifs			1 hit	STXBP4	intronic
17	55109176	0.92	0.97	rs244368	G	A	0.72	0.48	0.44	0.58						6 altered motifs			1 hit	STXBP4	intronic
17	55109755	0.89	0.97	rs244367	G	T	0.70	0.48	0.44	0.57						GR,Spz1			1 hit	STXBP4	intronic
17	55109939	0.91	0.97	rs244366	G	C	0.71	0.48	0.44	0.58						5 altered motifs			1 hit	STXBP4	intronic
17	55110040	0.92	0.97	rs244365	G	A	0.72	0.48	0.44	0.58						SIX5			1 hit	STXBP4	intronic
17	55110919	0.92	0.97	rs244364	C	T	0.76	0.48	0.44	0.58			SKIN, GI							STXBP4	intronic
17	55111532	0.92	0.97	rs244363	G	A	0.72	0.48	0.44	0.58								2 hits	1 hit	STXBP4	intronic
17	55111556	0.92	0.97	rs244362	T	C	0.78	0.48	0.44	0.58						BRCA1,CHD2,GR				STXBP4	intronic
17	55113065	0.89	0.96	rs171513	A	G	0.79	0.48	0.44	0.58						6 altered motifs				STXBP4	intronic
17	55113995	0.92	0.97	rs244361	A	G	0.79	0.48	0.44	0.58			BLD, SKIN							STXBP4	intronic
17	55114342	0.92	0.97	rs244360	T	C	0.76	0.48	0.44	0.58			BLD, SKIN	SKIN		RORalpha1				STXBP4	intronic
17	55115820	0.92	0.97	rs244357	A	G	0.79	0.48	0.44	0.58			BLD, SKIN			NRSF,Nrf-2			1 hit	STXBP4	intronic
17	55116448	0.92	0.97	rs244356	G	T	0.72	0.48	0.44	0.58			8 tissues	4 tissues		CEBPB,ELF1			1 hit	STXBP4	intronic
17	55116658	0.89	0.97	rs142715162	G	GA	0.76	0.48	0.44	0.58			9 tissues	LNG,LIV,VAS	GATA2,FOXA1,GATA3	9 altered motifs				STXBP4	intronic
17	55116724	0.92	0.97	rs244355	A	T	0.72	0.48	0.44	0.58			9 tissues	7 tissues	4 bound proteins	9 altered motifs			1 hit	STXBP4	intronic
17	55116781	0.92	0.97	rs244354	C	T	0.79	0.49	0.44	0.58			9 tissues	9 tissues	4 bound proteins	Sox				STXBP4	intronic
17	55118117	0.91	0.97	rs244352	T	C	0.79	0.48	0.44	0.58			6 tissues							STXBP4	intronic
17	55118205	0.91	0.97	rs244351	G	A	0.72	0.48	0.44	0.58			6 tissues	BRN		4 altered motifs			1 hit	STXBP4	intronic
17	55118572	0.9	0.97	rs57523515	GAAAT	G	0.71	0.48	0.44	0.58			BLD			5 altered motifs				STXBP4	intronic
17	55118771	0.91	0.97	rs244350	C	G	0.71	0.48	0.44	0.58						SIX5,Zfp187			1 hit	STXBP4	intronic
17	55118808	0.9	0.97	rs244349	G	A	0.70	0.48	0.44	0.58						Mef2,Myc			1 hit	STXBP4	intronic
17	55118989	0.91	0.97	rs244347	T	G	0.67	0.48	0.44	0.58						Gm397,Zbtb12			1 hit	STXBP4	intronic
17	55119022	0.92	0.97	rs244346	C	T	0.71	0.48	0.44	0.58						8 altered motifs			1 hit	STXBP4	intronic
17	55119616	0.9	0.97	rs244345	A	T	0.76	0.48	0.44	0.58						5 altered motifs				STXBP4	intronic
17	55119637	0.92	0.97	rs244344	A	G	0.72	0.48	0.44	0.58						11 altered motifs			1 hit	STXBP4	intronic
17	55120251	0.92	0.97	rs244343	A	G	0.72	0.48	0.44	0.58						4 altered motifs			1 hit	STXBP4	intronic
17	55121561	0.92	0.97	rs244341	G	A	0.72	0.48	0.44	0.58			FAT, PANC		EBF1	GATA,RFX5,VDR			1 hit	STXBP4	intronic
17	55121984	0.92	0.97	rs244340	G	A	0.72	0.48	0.44	0.58						Foxl1,Smad		2 hits	1 hit	STXBP4	intronic
17	55122753	0.92	0.97	rs244339	C	T	0.72	0.48	0.44	0.58									1 hit	STXBP4	intronic
17	55124569	0.9	0.96	rs244335	C	G	0.76	0.49	0.44	0.58			PANC, GI			Foxo				STXBP4	intronic
17	55125031	0.92	0.97	rs173408	G	A	0.76	0.49	0.44	0.58			4 tissues			GATA,Rad21				STXBP4	intronic
17	55125616	0.92	0.97	rs2628324	T	A	0.79	0.49	0.44	0.58			FAT, PANC			4 altered motifs				STXBP4	intronic
17	55125736	0.92	0.97	rs2628323	T	A	0.76	0.49	0.44	0.58			ESDR, FAT, PANC			NF-kappaB,Pax-4				STXBP4	intronic
17	55126694	0.92	0.97	rs244334	A	G	0.76	0.49	0.44	0.58						6 altered motifs				STXBP4	intronic
17	55126817	0.92	0.97	rs244333	C	T	0.78	0.49	0.44	0.58						HDAC2,p300				STXBP4	intronic
17	55127398	0.92	0.97	rs2058026	T	A	0.76	0.49	0.44	0.58						Foxp3,Pou1f1				STXBP4	intronic
17	55127498	0.91	0.97	rs2058027	T	A	0.76	0.49	0.44	0.58						Ncx,Pou2f2				STXBP4	intronic
17	55129103	0.9	0.97	rs2787492	C	T	0.78	0.49	0.43	0.58						5 altered motifs			1 hit	STXBP4	intronic
17	55129447	0.91	0.97	rs2529505	G	A	0.76	0.49	0.44	0.58						Brachyury,Mef2,Pax-4				STXBP4	intronic
17	55129700	0.92	0.97	rs2214980	A	G	0.79	0.49	0.44	0.58						4 altered motifs				STXBP4	intronic
17	55130632	0.92	0.97	rs2628320	A	G	0.76	0.49	0.44	0.58						HNF1,Irf,Obox3				STXBP4	intronic
17	55130690	0.92	0.97	rs2787490	C	T	0.76	0.49	0.44	0.58						8 altered motifs				STXBP4	intronic
17	55130775	0.92	0.97	rs2787489	T	A	0.76	0.49	0.44	0.58										STXBP4	intronic
17	55130790	0.92	0.97	rs2787488	T	C	0.75	0.49	0.44	0.58						GR				STXBP4	intronic
17	55132021	0.92	0.97	rs2787487	G	C	0.76	0.48	0.41	0.58						6 altered motifs				STXBP4	intronic
17	55132144	0.92	0.97	rs2170943	G	T	0.76	0.49	0.44	0.58						15 altered motifs				STXBP4	intronic
17	55132154	0.91	0.97	rs2170942	A	C	0.79	0.49	0.44	0.58						11 altered motifs				STXBP4	intronic
17	55132427	0.87	0.96	rs2787485	G	A	0.74	0.48	0.44	0.58						4 altered motifs			1 hit	STXBP4	intronic
17	55132759	0.92	0.97	rs2787484	C	T	0.78	0.49	0.44	0.58				ESDR						STXBP4	intronic
17	55132951	0.91	0.97	rs2628318	A	G	0.76	0.49	0.44	0.58						GR				STXBP4	intronic
17	55133413	0.92	0.97	rs2787482	G	A	0.76	0.49	0.44	0.58						6 altered motifs				STXBP4	intronic
17	55133816	0.92	0.97	rs2529506	C	T	0.76	0.49	0.44	0.58			FAT	GI,LIV		TLX1::NFIC				STXBP4	intronic
17	55134166	0.92	0.97	rs171510	G	A	0.76	0.49	0.44	0.58			FAT, MUS, SKIN	IPSC,SKIN		Pax-4,TATA				STXBP4	intronic
17	55134352	0.92	0.97	rs244307	A	G	0.76	0.49	0.44	0.58			FAT, MUS, SKIN	BLD		5 altered motifs				STXBP4	intronic
17	55134692	0.92	0.97	rs244308	A	G	0.76	0.49	0.44	0.58		FAT	4 tissues	11 tissues		4 altered motifs				STXBP4	intronic
17	55134732	0.92	0.97	rs244309	C	G	0.76	0.49	0.44	0.58		FAT	4 tissues	9 tissues		GR,T3R				STXBP4	intronic
17	55135211	0.87	0.97	rs2529508	A	T	0.76	0.49	0.44	0.57		FAT	4 tissues			5 altered motifs				STXBP4	intronic
17	55135294	0.92	0.97	rs244310	C	T	0.76	0.49	0.44	0.58			FAT			16 altered motifs				STXBP4	intronic
17	55135442	0.92	0.97	rs244311	G	A	0.76	0.49	0.44	0.58			FAT			14 altered motifs				STXBP4	intronic
17	55135836	0.92	0.97	rs244312	A	G	0.76	0.49	0.44	0.58			FAT			4 altered motifs				STXBP4	intronic
17	55136971	0.92	0.97	rs244313	C	T	0.76	0.49	0.44	0.58						Foxj1,Sox				STXBP4	intronic
17	55137156	0.92	0.97	rs244314	G	A	0.75	0.49	0.44	0.58						5 altered motifs			1 hit	STXBP4	intronic
17	55137271	0.89	0.96	rs2787473	T	C	0.76	0.49	0.44	0.58						7 altered motifs				STXBP4	intronic
17	55137273	0.92	0.97	rs2787474	T	C	0.76	0.49	0.44	0.58						8 altered motifs				STXBP4	intronic
17	55137862	0.93	0.97	rs244316	G	A	0.79	0.49	0.44	0.58										STXBP4	intronic
17	55151984	0.95	0.98	rs1994234	A	G	0.54	0.46	0.44	0.59						Foxp1,RREB-1			3 hits	STXBP4	intronic
17	55153361	0.99	0.99	rs244293	A	G	0.66	0.47	0.40	0.59						4 altered motifs	1 hit	1 hit	1 hit	STXBP4	intronic
17	55153373	0.98	0.99	rs244292	A	G	0.82	0.49	0.43	0.59						5 altered motifs		2 hits	2 hits	STXBP4	intronic
17	55153776	0.99	0.99	rs244291	G	A	0.66	0.47	0.40	0.59						Foxa,Tgif1			1 hit	STXBP4	intronic
17	55153870	0.98	0.99	rs244290	A	G	0.78	0.49	0.43	0.59						5 altered motifs			2 hits	STXBP4	intronic
17	55154427	0.98	0.99	rs244289	A	G	0.83	0.48	0.43	0.59			LIV		SIX5	6 altered motifs			2 hits	STXBP4	intronic
17	55154480	0.98	0.99	rs244288	G	A	0.78	0.49	0.43	0.59			LIV			GATA,HNF1,Nrf-2			1 hit	STXBP4	intronic
17	55154675	0.98	0.99	rs244287	T	C	0.82	0.49	0.43	0.59			LIV			Mef2			2 hits	STXBP4	intronic
17	55155233	0.99	1	rs244286	A	G	0.78	0.49	0.43	0.59			LIV			9 altered motifs			1 hit	STXBP4	intronic
17	55157821	1	1	rs2628314	T	C	0.65	0.47	0.40	0.59			7 tissues			Arid5b,p300			1 hit	STXBP4	intronic
17	55157991	0.99	1	rs2628313	T	C	0.83	0.49	0.43	0.59			6 tissues						2 hits	STXBP4	intronic
17	55158613	0.99	1	rs183618	A	G	0.78	0.49	0.43	0.59			7 tissues	12 tissues	CFOS,STAT3,CTCF	Hdx			1 hit	STXBP4	intronic
17	55160322	0.99	1	rs244306	T	C	0.78	0.49	0.43	0.59			ESDR, MUS, BRN	OVRY		Pou2f2,Sox			1 hit	STXBP4	3'-UTR
17	55160482	0.99	1	rs244305	A	G	0.78	0.49	0.43	0.59			ESDR, MUS, BRN			THAP1			1 hit	STXBP4	3'-UTR
17	55161367	1	1	rs244303	G	A	0.65	0.47	0.40	0.59			MUS, SKIN, BRN			Hsf,Ik-2		1 hit	1 hit	STXBP4	3'-UTR
17	55161779	0.99	1	rs244301	C	T	0.77	0.49	0.43	0.59			5 tissues			4 altered motifs			1 hit	STXBP4	3'-UTR
17	55161879	1	1	rs244300	T	A	0.65	0.47	0.40	0.59			8 tissues			Nkx2,Nkx3,Pax-5		1 hit	1 hit	STXBP4	3'-UTR
17	55162905	0.99	1	rs244299	C	A	0.65	0.47	0.40	0.59			6 tissues	ESDR		HMG-IY,Hmx			1 hit	STXBP4	3'-UTR
17	55164026	1	1	rs244298	C	T	0.65	0.47	0.40	0.59			MUS, BLD, SKIN	SKIN,LNG				1 hit	1 hit	STXBP4	3'-UTR
17	55164339	0.99	0.99	rs244297	A	G	0.78	0.48	0.43	0.59			MUS			4 altered motifs				53bp 3' of STXBP4	
17	55164544	0.91	0.99	rs173407	G	C	0.81	0.48	0.43	0.57						BDP1,ELF1,Ets			1 hit	258bp 3' of STXBP4	
17	55165517	0.99	1	rs244296	A	G	0.83	0.49	0.43	0.59				SKIN		6 altered motifs			2 hits	1.2kb 3' of STXBP4	
17	55165538	0.99	1	rs244295	T	A	0.77	0.49	0.43	0.59						PU.1			1 hit	1.3kb 3' of STXBP4	

Query SNP: rs388685 and variants with r^2 >= 0.8

chr	pos (hg38)	LD (r^2)	LD (D')	variant	Ref	Alt	AFR freq	AMR freq	ASN freq	EUR freq	SiPhy cons	Promoter histone marks	Enhancer histone marks	DNAse	Proteins bound	Motifs changed	NHGRI/EBI GWAS hits	GRASP QTL hits	Selected eQTL hits	GENCODE genes	dbSNP func annot
19	43853890	0.81	0.94	rs4802201	C	T	0.23	0.51	0.57	0.49						5 altered motifs			42 hits	4.7kb 3' of ZNF283	
19	43855412	0.93	0.97	rs4803662	C	G	0.39	0.58	0.83	0.51						5 altered motifs		4 hits	52 hits	6.3kb 3' of ZNF283	
19	43863186	0.94	0.97	rs8104290	A	G	0.62	0.62	0.83	0.51								7 hits	50 hits	9.2kb 3' of ZNF404	
19	43863459	0.94	0.97	rs2191564	T	C	0.62	0.62	0.83	0.51				LNG		18 altered motifs			50 hits	8.9kb 3' of ZNF404	
19	43863691	0.95	0.97	rs10416434	G	A	0.31	0.57	0.83	0.51						LUN-1,SP1			51 hits	8.7kb 3' of ZNF404	
19	43863759	0.91	0.98	rs10417741	T	C	0.25	0.51	0.57	0.49						5 altered motifs			55 hits	8.6kb 3' of ZNF404	
19	43867058	0.9	0.97	rs8106090	A	G	0.60	0.62	0.83	0.50						ZEB1			51 hits	5.3kb 3' of ZNF404	
19	43867241	0.87	0.98	rs12985878	G	A	0.25	0.49	0.55	0.48				IPSC		Gfi1,Hdx			48 hits	5.1kb 3' of ZNF404	
19	43870479	0.94	0.97	rs11673020	A	G	0.62	0.62	0.83	0.51		HRT				Cdx,Cdx2,Irf		4 hits	51 hits	1.9kb 3' of ZNF404	
19	43870564	0.81	0.97	rs239943	A	T	0.83	0.69	0.83	0.55		HRT				BCL,Hoxa13		5 hits	42 hits	1.8kb 3' of ZNF404	
19	43871799	0.93	0.97	rs57202192	TAATA	T	0.38	0.58	0.83	0.51		HRT				13 altered motifs			50 hits	567bp 3' of ZNF404	
19	43872529	0.94	0.97	rs1050054	G	T	0.39	0.58	0.83	0.51					CEBPB			4 hits	52 hits	ZNF404	3'-UTR
19	43873517	0.95	0.98	rs12977303	G	A	0.11	0.50	0.57	0.51								4 hits	48 hits	ZNF404	missense
19	43874725	0.95	0.97	rs10406290	G	T	0.31	0.57	0.83	0.51						Foxp1,Pou2f2		4 hits	52 hits	ZNF404	intronic
19	43875402	0.81	0.97	rs171238	A	C	0.83	0.69	0.83	0.55								6 hits	43 hits	ZNF404	intronic
19	43876384	0.94	0.97	rs10414702	A	G	0.62	0.62	0.83	0.51						9 altered motifs			49 hits	ZNF404	intronic
19	43877134	0.94	0.97	rs10421518	A	G	0.62	0.62	0.83	0.51						7 altered motifs			49 hits	ZNF404	intronic
19	43878931	0.94	0.97	rs7258418	G	C	0.62	0.62	0.83	0.51						Myc			49 hits	ZNF404	intronic
19	43879648	0.94	0.97	rs1978723	T	C	0.62	0.62	0.83	0.51						11 altered motifs		4 hits	50 hits	ZNF404	intronic
19	43883815	0.81	0.97	rs239939	A	T	0.83	0.69	0.83	0.55								2 hits	42 hits	3.7kb 5' of ZNF404	intronic
19	43886153	0.82	0.98	rs239938	T	C	0.83	0.69	0.83	0.55						p53		4 hits	42 hits	6kb 5' of ZNF404	
19	43889105	0.96	0.98	rs371875	A	G	0.26	0.57	0.83	0.51						ZBTB33			46 hits	9kb 5' of ZNF404	
19	43889204	0.95	0.98	rs368079	C	T	0.34	0.58	0.83	0.51						Brachyury,Irf,TCF4			50 hits	9.1kb 5' of ZNF404	
19	43889823	0.97	0.99	rs425217	C	T	0.21	0.51	0.57	0.51						5 altered motifs			45 hits	9.7kb 5' of ZNF404	
19	43890573	0.95	0.98	rs71169290	9-mer	A	0.26	0.50	0.57	0.51						10 altered motifs			47 hits	10kb 5' of ZNF404	
19	43891337	0.97	0.99	rs429027	G	A	0.25	0.51	0.58	0.51						Foxp1		4 hits	48 hits	11kb 5' of ZNF404	
19	43893073	0.95	0.98	rs396874	G	C	0.62	0.62	0.83	0.51						4 altered motifs			49 hits	13kb 5' of ZNF404	
19	43894384	0.94	0.98	rs71169291	CT	C	0.30	0.56	0.82	0.50						8 altered motifs			48 hits	14kb 5' of ZNF404	intronic
19	43894679	0.97	0.99	rs432454	C	G	0.21	0.50	0.57	0.51						Gfi1,NF-AT,PPAR		9 hits	46 hits	15kb 5' of ZNF404	intronic
19	43894788	0.94	0.97	rs397913	A	G	0.39	0.57	0.83	0.51						Mrg		8 hits	52 hits	15kb 5' of ZNF404	intronic
19	43894932	0.95	0.98	rs368089	T	C	0.39	0.57	0.83	0.51						ERalpha-a,Hoxa5		8 hits	52 hits	15kb 5' of ZNF404	intronic
19	43894963	0.95	0.98	rs397346	A	G	0.62	0.62	0.83	0.51								4 hits	50 hits	15kb 5' of ZNF404	intronic
19	43895154	0.95	0.98	rs396973	A	G	0.62	0.62	0.83	0.51						4 altered motifs		2 hits	49 hits	15kb 5' of ZNF404	intronic
19	43895413	0.95	0.98	rs436249	C	T	0.39	0.57	0.83	0.51						Elf5		2 hits	51 hits	15kb 5' of ZNF404	intronic
19	43896223	0.95	0.98	rs430308	C	G	0.38	0.57	0.83	0.51			BRST		CEBPB	6 altered motifs		8 hits	52 hits	16kb 5' of ZNF404	intronic
19	43896511	0.82	-0.98	rs12610287	T	C	0.17	0.31	0.17	0.45						Nanog			41 hits	16kb 3' of ZNF45	intronic
19	43897641	0.95	0.98	rs372491	G	A	0.38	0.57	0.83	0.51						LBP-9		4 hits	52 hits	15kb 3' of ZNF45	intronic
19	43898422	0.95	0.98	rs415168	C	G	0.38	0.57	0.83	0.51				PLCNT		6 altered motifs		4 hits	52 hits	14kb 3' of ZNF45	intronic
19	43898903	0.95	0.98	rs453950	G	A	0.38	0.57	0.83	0.51								4 hits	52 hits	14kb 3' of ZNF45	intronic
19	43900190	0.95	0.98	rs440784	T	C	0.38	0.57	0.83	0.51						8 altered motifs			51 hits	12kb 3' of ZNF45	intronic
19	43900246	0.95	0.98	rs374307	G	T	0.38	0.57	0.83	0.51						GR			51 hits	12kb 3' of ZNF45	intronic
19	43900530	0.95	0.98	rs441344	T	C	0.38	0.57	0.83	0.51		ESDR, BLD, BRN	BLD, STRM, BRN			DMRT2,DMRT3			51 hits	12kb 3' of ZNF45	intronic
19	43900892	0.95	0.98	rs384522	A	T	0.39	0.57	0.83	0.51		8 tissues	10 tissues			CEBPA,p300			51 hits	12kb 3' of ZNF45	
19	43901129	0.95	0.98	rs398099	G	C	0.38	0.57	0.83	0.51		16 tissues	14 tissues			NR4A			51 hits	11kb 3' of ZNF45	
19	43901135	0.95	0.98	rs385321	C	T	0.38	0.57	0.83	0.51		16 tissues	14 tissues						51 hits	11kb 3' of ZNF45	
19	43901770	0.95	0.98	rs108775	G	C	0.62	0.61	0.83	0.51				51 tissues	9 bound proteins	Mxi1			49 hits	11kb 3' of ZNF45	
19	43901975	0.96	0.98	rs367283	A	G	0.41	0.58	0.83	0.51		23 tissues		44 tissues	7 bound proteins	COMP1,Mef2		4 hits	51 hits	11kb 3' of ZNF45	
19	43902139	0.96	0.98	rs448823	T	C	0.41	0.58	0.83	0.51		22 tissues		18 tissues	SETDB1				50 hits	10kb 3' of ZNF45	
19	43902169	0.96	0.98	rs448829	T	C	0.41	0.58	0.83	0.51		22 tissues		11 tissues	SETDB1	5 altered motifs		4 hits	51 hits	10kb 3' of ZNF45	
19	43903335	0.97	0.99	rs376328	A	G	0.38	0.57	0.83	0.51						Gfi1,HMG-IY,Myc		4 hits	52 hits	9.3kb 3' of ZNF45	
19	43903412	0.98	0.99	rs376032	A	G	0.11	0.49	0.57	0.51				HRT		SP1		4 hits	48 hits	9.2kb 3' of ZNF45	
19	43904047	0.97	0.99	rs365556	A	G	0.38	0.57	0.83	0.51		GI				DMRT5		4 hits	52 hits	8.6kb 3' of ZNF45	
19	43904601	0.97	0.99	rs451945	T	C	0.38	0.57	0.83	0.51						9 altered motifs			51 hits	8kb 3' of ZNF45	
19	43904821	0.97	0.99	rs454813	A	C	0.38	0.57	0.83	0.51				LNG		DMRT7		4 hits	52 hits	7.8kb 3' of ZNF45	
19	43905259	0.98	0.99	rs422457	A	T	0.21	0.50	0.57	0.51						E2A,Pitx2			45 hits	7.4kb 3' of ZNF45	
19	43906694	0.97	0.99	rs417400	A	G	0.38	0.57	0.83	0.51						5 altered motifs		8 hits	52 hits	5.9kb 3' of ZNF45	
19	43907268	0.84	-0.99	rs1073654	T	C	0.17	0.31	0.17	0.45						GR		2 hits	42 hits	5.4kb 3' of ZNF45	
19	43907391	0.84	-0.99	rs1073653	G	A	0.17	0.31	0.17	0.45								4 hits	42 hits	5.2kb 3' of ZNF45	
19	43908431	0.97	0.99	rs426534	T	G	0.38	0.57	0.83	0.51						4 altered motifs			51 hits	4.2kb 3' of ZNF45	
19	43908605	0.97	0.99	rs376457	C	G	0.38	0.57	0.83	0.51						NF-kappaB		4 hits	54 hits	4kb 3' of ZNF45	
19	43909101	0.97	0.99	rs430667	T	C	0.38	0.57	0.83	0.51			HRT			HNF1,Maf,Tgif1			51 hits	3.5kb 3' of ZNF45	
19	43909732	0.97	0.99	rs398964	G	A	0.38	0.57	0.83	0.51						CEBPB,SRF,Zfp187			51 hits	2.9kb 3' of ZNF45	
19	43910315	0.97	0.99	rs439665	G	C	0.38	0.57	0.83	0.51						HP1-site-factor,Pax-6			51 hits	2.3kb 3' of ZNF45	
19	43910664	0.84	-0.99	rs62114648	T	C	0.17	0.31	0.17	0.45						GR,NR4A			41 hits	2kb 3' of ZNF45	
19	43911428	0.98	0.99	rs421512	A	T	0.25	0.51	0.57	0.51						8 altered motifs			46 hits	1.2kb 3' of ZNF45	
19	43912047	0.97	0.99	rs423765	C	T	0.38	0.57	0.83	0.51						BAF155			48 hits	576bp 3' of ZNF45	
19	43912415	0.84	-0.99	rs62114649	T	C	0.17	0.31	0.17	0.45						Pou2f2			40 hits	208bp 3' of ZNF45	
19	43913143	0.97	0.99	rs376069	T	C	0.38	0.57	0.83	0.51				IPSC		5 altered motifs		4 hits	53 hits	ZNF45	3'-UTR
19	43913423	0.97	0.99	rs417699	A	G	0.44	0.59	0.83	0.51				ESDR		STAT		8 hits	51 hits	ZNF45	synonymous
19	43913925	0.98	1	rs407731	C	T	0.38	0.57	0.82	0.51				SKIN		5 altered motifs		8 hits	52 hits	ZNF45	missense
19	43914191	0.98	1	rs406968	C	T	0.38	0.58	0.82	0.51				LNG,SKIN	SETDB1	TAL1			49 hits	ZNF45	synonymous
19	43914392	0.98	1	rs425221	A	G	0.38	0.58	0.82	0.51				5 tissues	SETDB1	7 altered motifs		8 hits	52 hits	ZNF45	synonymous
19	43914528	1	1	rs388685	G	C	0.25	0.51	0.57	0.51				ESDR,GI,LNG	SETDB1			7 hits	48 hits	ZNF45	missense
19	43914541	0.98	1	rs388706	T	C	0.38	0.58	0.82	0.51				ESDR	SETDB1	Pax-8,RAR		8 hits	52 hits	ZNF45	missense
19	43914672	1	1	rs399098	C	T	0.25	0.51	0.57	0.51					SETDB1	RBP-Jkappa,TEF-1		7 hits	48 hits	ZNF45	missense
19	43915506	1	1	rs438517	G	A	0.25	0.51	0.57	0.51						4 altered motifs			45 hits	ZNF45	intronic
19	43915959	0.98	1	rs387689	C	T	0.38	0.58	0.82	0.51						5 altered motifs			49 hits	ZNF45	intronic
19	43916268	0.98	1	rs398193	T	C	0.38	0.58	0.82	0.51						Brachyury,RREB-1,TBX5			48 hits	ZNF45	intronic
19	43916855	1	1	rs446674	G	A	0.25	0.51	0.57	0.51						CDP,Pou1f1			45 hits	ZNF45	intronic
19	43917073	0.85	-1	rs17713001	C	T	0.18	0.31	0.18	0.45						4 altered motifs			41 hits	ZNF45	intronic
19	43917364	0.85	-1	rs11881193	G	T	0.18	0.31	0.18	0.45						4 altered motifs			41 hits	ZNF45	intronic
19	43917777	0.98	0.99	rs378112	T	C	0.38	0.57	0.82	0.51									48 hits	ZNF45	intronic
19	43917785	0.97	0.99	rs378109	T	C	0.38	0.57	0.82	0.51						Irf		4 hits	51 hits	ZNF45	intronic
19	43918206	0.98	1	rs450308	C	A	0.38	0.58	0.82	0.51						Myc,SREBP			48 hits	ZNF45	intronic
19	43918437	0.85	-1	rs2284245	A	T	0.18	0.31	0.18	0.45						Pax-4			41 hits	ZNF45	intronic
19	43918650	0.98	1	rs424729	G	C	0.38	0.58	0.82	0.51								4 hits	51 hits	ZNF45	intronic
19	43918830	0.98	1	rs423320	G	A	0.38	0.58	0.82	0.51						LRH1		4 hits	51 hits	ZNF45	intronic
19	43919043	0.98	1	rs423752	G	A	0.38	0.58	0.82	0.51						5 altered motifs		4 hits	51 hits	ZNF45	intronic
19	43919418	0.98	1	rs375066	T	C	0.38	0.58	0.82	0.51								4 hits	51 hits	ZNF45	intronic
19	43919829	0.98	1	rs384329	T	C	0.38	0.58	0.82	0.51						5 altered motifs		7 hits	51 hits	ZNF45	intronic
19	43920102	0.97	0.99	rs370190	A	T	0.38	0.58	0.82	0.51						4 altered motifs			41 hits	ZNF45	intronic
19	43920642	0.97	0.99	rs424410	C	G	0.38	0.56	0.82	0.51						AP-2,EBF,Irf			45 hits	ZNF45	intronic
19	43920925	0.85	-1	rs17656688	T	C	0.18	0.31	0.18	0.45						Irf,Pou2f2		2 hits	44 hits	ZNF45	intronic
19	43921467	0.97	0.99	rs408549	T	A	0.38	0.58	0.82	0.51						Bcl6b,CEBPB,Pou5f1			48 hits	ZNF45	intronic
19	43921530	0.97	0.99	rs398388	C	T	0.38	0.58	0.82	0.51						BCL,E2F,HNF1			48 hits	ZNF45	intronic
19	43922113	0.97	0.99	rs413093	A	G	0.38	0.58	0.82	0.51				ESDR		TBX5		4 hits	51 hits	ZNF45	intronic
19	43922311	0.92	0.96	rs201533198	G	GT	0.39	0.58	0.82	0.51						5 altered motifs			40 hits	ZNF45	intronic
19	43922315	0.97	0.99	rs57712519	T	TG	0.33	0.58	0.82	0.51						14 altered motifs			45 hits	ZNF45	intronic
19	43923474	0.97	0.99	rs403137	A	G	0.38	0.58	0.82	0.51						Cdc5,Nkx3		4 hits	51 hits	ZNF45	intronic
19	43923949	0.97	0.99	rs379785	T	C	0.37	0.57	0.82	0.51						Mef2		4 hits	49 hits	ZNF45	intronic
19	43924759	0.97	0.99	rs428505	G	C	0.38	0.58	0.82	0.51						p53			48 hits	ZNF45	intronic
19	43924891	0.97	0.99	rs142243559	TAAAC	T	0.38	0.58	0.82	0.51						11 altered motifs			6 hits	ZNF45	intronic
19	43924968	0.99	0.99	rs454559	C	T	0.21	0.50	0.57	0.51						NERF1a,Nanog		4 hits	50 hits	ZNF45	intronic
19	43925028	0.97	0.99	rs367741	T	C	0.38	0.58	0.82	0.51				BLD		RREB-1,SP1,UF1H3BETA		4 hits	51 hits	ZNF45	intronic
19	43926717	0.97	0.99	rs373168	C	T	0.38	0.58	0.82	0.51						TEF-1		4 hits	49 hits	ZNF45	intronic
19	43927742	0.98	0.99	rs368316	C	A	0.30	0.57	0.82	0.51						GR			46 hits	ZNF45	intronic
19	43928688	0.97	0.99	rs411803	T	C	0.40	0.58	0.82	0.51								2 hits	45 hits	ZNF45	intronic

Query SNP: rs1017968 and variants with r^2 >= 0.8

chr	pos (hg38)	LD (r^2)	LD (D')	variant	Ref	Alt	AFR freq	AMR freq	ASN freq	EUR freq	SiPhy cons	Promoter histone marks	Enhancer histone marks	DNAse	Proteins bound	Motifs changed	NHGRI/EBI GWAS hits	GRASP QTL hits	Selected eQTL hits	GENCODE genes	dbSNP func annot
3	99961242	0.8	-0.9	rs4075038	A	G	0.91	0.78	0.89	0.79		FAT, GI	11 tissues	4 tissues		5 altered motifs			5 hits	C3orf26	intronic
3	99986017	0.89	-0.95	rs4928234	T	C	0.90	0.78	0.89	0.79						Arnt,E2A,Myc			4 hits	C3orf26	intronic
3	99986267	0.87	-0.95	rs4571265	A	G	0.90	0.78	0.89	0.79						ZBRK1,Zfp410			7 hits	C3orf26	intronic
3	99988402	0.84	-0.95	rs9834484	A	G	0.87	0.78	0.89	0.78			ESC, IPSC			8 altered motifs			2 hits	C3orf26	intronic
3	99992843	0.87	0.96	rs9873709	G	A	0.15	0.23	0.11	0.22						CEBPB,Foxj1,HNF1			2 hits	C3orf26	intronic
3	99992972	0.91	0.96	rs9873878	G	T	0.10	0.22	0.11	0.21									5 hits	C3orf26	intronic
3	99993790	0.91	0.96	rs9878931	G	A	0.10	0.22	0.11	0.21						4 altered motifs			5 hits	C3orf26	intronic
3	99994533	0.91	0.96	rs10212525	T	C	0.10	0.22	0.11	0.21						Gfi1,Pax-4			4 hits	C3orf26	intronic
3	99996011	0.91	0.96	rs9852435	T	C	0.09	0.22	0.11	0.21						5 altered motifs			4 hits	C3orf26	intronic
3	99997846	0.91	0.96	rs35521705	C	T	0.10	0.21	0.11	0.21			FAT, VAS, BRN			Bach1,Brachyury			4 hits	C3orf26	intronic
3	100004736	0.91	0.96	rs9833888	G	T	0.09	0.21	0.11	0.21			9 tissues						5 hits	C3orf26	intronic
3	100005948	0.87	0.96	rs17379739	G	A	0.13	0.22	0.11	0.22			18 tissues	4 tissues		Pax-5			2 hits	C3orf26	intronic
3	100006029	0.87	0.96	rs10511177	G	A	0.13	0.22	0.11	0.22						7 altered motifs			3 hits	C3orf26	intronic
3	100009659	0.91	0.96	rs9832186	G	C	0.09	0.21	0.11	0.21			8 tissues	4 tissues		6 altered motifs			4 hits	C3orf26	intronic
3	100010247	0.87	0.96	rs9875640	C	T	0.10	0.22	0.11	0.22			6 tissues	MUS,VAS		5 altered motifs			3 hits	C3orf26	intronic
3	100010286	0.87	0.96	rs9875664	C	T	0.13	0.22	0.11	0.22			6 tissues			GR,HDAC2			2 hits	C3orf26	intronic
3	100013720	0.91	0.96	rs7610486	A	G	0.07	0.21	0.11	0.21						Foxa,PLZF,p300			4 hits	C3orf26	intronic
3	100014252	0.91	0.96	rs4279135	C	T	0.08	0.21	0.11	0.21									6 hits	C3orf26	intronic
3	100017694	0.91	0.96	rs9810235	C	T	0.08	0.21	0.11	0.21						7 altered motifs			4 hits	C3orf26	intronic
3	100021016	0.87	0.96	rs9853823	T	C	0.13	0.22	0.11	0.22			8 tissues			PLAG1,YY1,Zfx			2 hits	C3orf26	intronic
3	100029423	0.91	0.96	rs34230430	CTAAA	C	0.08	0.21	0.11	0.21			GI, MUS, BLD			5 altered motifs			2 hits	C3orf26	intronic
3	100029704	0.91	0.96	rs10049383	T	C	0.07	0.21	0.11	0.21						4 altered motifs			7 hits	C3orf26	intronic
3	100033224	0.91	0.96	rs28714363	G	T	0.08	0.21	0.11	0.21		SKIN	16 tissues	10 tissues	STAT3,P300	8 altered motifs			6 hits	C3orf26	intronic
3	100037470	0.91	0.96	rs35701020	A	G	0.07	0.21	0.10	0.21						GATA,Sox			6 hits	C3orf26	intronic
3	100038948	0.94	0.98	rs923470	G	A	0.08	0.21	0.11	0.21			ESDR, GI, PANC			6 altered motifs			6 hits	C3orf26	intronic
3	100039009	0.94	0.98	rs923471	G	A	0.08	0.21	0.11	0.21			GI, PANC			BRCA1			5 hits	C3orf26	intronic
3	100042020	0.89	0.98	rs6807176	A	G	0.14	0.23	0.11	0.22		4 tissues	15 tissues	5 tissues		Irf			3 hits	C3orf26	intronic
3	100043064	0.93	0.99	rs17393059	A	G	0.13	0.22	0.11	0.22		5 tissues	18 tissues	12 tissues		4 altered motifs			2 hits	C3orf26	intronic
3	100043996	0.93	0.99	rs6799379	C	T	0.13	0.22	0.11	0.22			7 tissues	BLD		LBP-9,YY1			2 hits	C3orf26	intronic
3	100045910	0.97	0.99	rs9864437	G	A	0.08	0.21	0.11	0.21			19 tissues	35 tissues	P300	6 altered motifs			5 hits	C3orf26	intronic
3	100047219	0.97	0.99	rs35669453	G	A	0.08	0.21	0.11	0.21		STRM, BLD, GI	17 tissues	4 tissues	PU1	Pax-4			4 hits	C3orf26	intronic
3	100048252	0.97	0.99	rs9833980	A	G	0.08	0.21	0.11	0.21		BLD, GI, MUS	19 tissues	SKIN,MUS					6 hits	C3orf26	intronic
3	100048565	0.98	0.99	rs9851645	G	C	0.07	0.21	0.11	0.21		BLD	11 tissues	4 tissues		Pax-2			5 hits	C3orf26	intronic
3	100048697	0.97	0.99	rs13064704	G	A	0.08	0.21	0.11	0.21		BLD	11 tissues			7 altered motifs			5 hits	C3orf26	intronic
3	100048798	0.96	0.98	rs62285461	C	T	0.08	0.21	0.11	0.21			5 tissues			GR,HDAC2			4 hits	C3orf26	intronic
3	100050244	0.97	0.99	rs1489816	C	G	0.08	0.21	0.10	0.21						TCF11::MafG			5 hits	C3orf26	intronic
3	100050990	0.97	0.99	rs62285462	T	A	0.08	0.21	0.11	0.21									4 hits	C3orf26	intronic
3	100051852	0.94	0.97	rs71313585	A	G	0.09	0.20	0.11	0.21				OVRY		4 altered motifs			3 hits	C3orf26	intronic
3	100059811	0.97	0.99	rs6802422	T	C	0.08	0.21	0.11	0.21			5 tissues			AP-1,Foxp1			6 hits	C3orf26	intronic
3	100060814	0.97	0.99	rs6790877	C	T	0.08	0.21	0.11	0.21			6 tissues	OVRY,BLD	EGR1,GATA2,TAL1	NR4A,Pax-5			5 hits	C3orf26	intronic
3	100061549	0.93	0.99	rs6806178	A	C	0.13	0.22	0.11	0.22			BLD, GI, LIV			BATF,Irf,PLZF			2 hits	C3orf26	intronic
3	100062961	0.97	0.99	rs2291494	G	A	0.08	0.21	0.11	0.21			5 tissues	OVRY					4 hits	C3orf26	intronic
3	100063383	0.97	0.99	rs2291495	T	G	0.08	0.21	0.11	0.21			LIV, LNG			4 altered motifs			4 hits	C3orf26	intronic
3	100063731	0.97	0.99	rs11317963	GA	G	0.10	0.21	0.11	0.21			LIV, LNG			Foxp1,GATA,HDAC2				C3orf26	intronic
3	100066362	0.95	1	rs9815439	G	C	0.14	0.23	0.11	0.22			LIV	ESDR,LNG		LF-A1,Pou2f2,Rad21			3 hits	C3orf26	intronic
3	100067445	0.95	1	rs1021341	A	G	0.13	0.22	0.11	0.22						Hoxa4,Nr2f2			3 hits	C3orf26	intronic
3	100068167	1	1	rs1017968	T	C	0.07	0.21	0.11	0.21						5 altered motifs			4 hits	C3orf26	intronic
3	100071488	1	1	rs7620228	A	C	0.07	0.21	0.11	0.21			14 tissues			TCF4			4 hits	C3orf26	intronic
3	100075060	1	1	rs34355464	TA	T	0.10	0.22	0.11	0.21			7 tissues			15 altered motifs			3 hits	C3orf26	intronic
3	100075886	0.95	1	rs34922392	G	T	0.13	0.22	0.11	0.22			8 tissues	4 tissues		4 altered motifs			2 hits	C3orf26	intronic
3	100075998	0.95	1	rs28503408	G	A	0.13	0.22	0.11	0.22		ESDR	10 tissues	4 tissues	P300,TCF4	Homez,SIX5			2 hits	C3orf26	intronic
3	100076663	1	1	rs9823908	A	G	0.07	0.21	0.11	0.21		ESDR, GI, LIV	17 tissues	5 tissues		HMG-IY,NRSF			4 hits	C3orf26	intronic
3	100076807	1	1	rs9841186	G	C	0.07	0.21	0.11	0.21		4 tissues	18 tissues	4 tissues		CEBPG,HNF1,Ncx			3 hits	C3orf26	intronic
3	100077782	1	1	rs35844878	T	C	0.08	0.21	0.11	0.21			7 tissues			Pax-4,Sin3Ak-20,p53			3 hits	C3orf26	intronic
3	100078169	0.95	1	rs9814359	C	G	0.13	0.22	0.11	0.22			GI, MUS, LIV			HNF4			3 hits	C3orf26	intronic
3	100082784	0.98	1	rs13069311	A	C	0.07	0.21	0.11	0.20		BLD	8 tissues	MUS,BLD		Foxj2,TAL1			4 hits	C3orf26	intronic
3	100087801	0.99	1	rs9828079	A	G	0.07	0.21	0.11	0.21			5 tissues			Pax-2			4 hits	C3orf26	intronic
3	100088105	0.98	0.99	rs62285471	A	G	0.07	0.21	0.11	0.21			7 tissues			Pitx2			3 hits	C3orf26	intronic
3	100088891	0.93	0.98	rs6790535	G	A	0.13	0.22	0.11	0.21		IPSC	10 tissues	ESDR,ESC	4 bound proteins	20 altered motifs			3 hits	C3orf26	intronic
3	100089494	0.98	0.99	rs7627991	A	G	0.08	0.21	0.11	0.21			10 tissues			Pax-4,Pou5f1			4 hits	C3orf26	intronic
3	100091111	0.98	0.99	rs62285473	C	T	0.08	0.21	0.11	0.21			BLD, LNG, MUS			Ets,ZBTB33			3 hits	C3orf26	intronic
3	100092709	0.97	0.98	rs13323143	T	C	0.08	0.21	0.12	0.21			ESDR, MUS			HDAC2			4 hits	C3orf26	intronic
3	100094374	0.98	0.99	rs35885423	C	G	0.07	0.21	0.11	0.21			MUS			YY1			3 hits	C3orf26	intronic
3	100094778	0.81	0.97	rs28870092	G	A	0.07	0.20	0.02	0.18			MUS			SP1			3 hits	C3orf26	intronic
3	100103291	0.98	0.99	rs62285478	C	T	0.08	0.21	0.11	0.21		MUS	14 tissues	ADRL,GI	ERALPHA_A	AP-1,GATA,Smad3			3 hits	C3orf26	intronic
3	100105841	0.98	0.99	rs9837602	G	A	0.08	0.21	0.11	0.21		MUS, BLD	18 tissues	BLD,MUS,BLD	EBF1,CEBPB	Pou2f2,SIX5			5 hits	C3orf26	intronic
3	100123296	0.85	0.94	rs13093776	C	T	0.27	0.22	0.17	0.21			6 tissues	ESDR,IPSC,IPSC		AP-2,BDP1			2 hits	C3orf26	intronic

Query SNP: rs1802212 and variants with r^2 >= 0.8

chr	pos (hg38)	LD (r^2)	LD (D')	variant	Ref	Alt	AFR freq	AMR freq	ASN freq	EUR freq	SiPhy cons	Promoter histone marks	Enhancer histone marks	DNAse	Proteins bound	Motifs changed	NHGRI/EBI GWAS hits	GRASP QTL hits	Selected eQTL hits	GENCODE genes	dbSNP func annot
17	54907990	0.84	0.94	rs9897646	G	C	0.17	0.19	0.12	0.29		GI	7 tissues			PU.1			21 hits	TOM1L1	intronic
17	54909484	0.84	0.94	rs9899545	T	C	0.18	0.19	0.12	0.29			4 tissues			EWSR1-FLI1			23 hits	TOM1L1	intronic
17	54909547	0.84	0.94	rs9899602	T	C	0.18	0.19	0.12	0.29			4 tissues			7 altered motifs		3 hits	23 hits	TOM1L1	intronic
17	54910871	0.84	0.94	rs17745123	G	T	0.16	0.19	0.12	0.29						KAP1,SP1		2 hits	23 hits	TOM1L1	intronic
17	54916595	0.82	0.95	rs66898273	C	T	0.18	0.21	0.12	0.30						Pax-4,TCF4			22 hits	TOM1L1	intronic
17	54916849	0.81	0.94	rs35079296	T	G	0.18	0.21	0.12	0.29					KAP1	Nkx2,Nkx3			22 hits	TOM1L1	intronic
17	54918789	0.82	0.95	rs1962045	C	T	0.18	0.21	0.12	0.30			ESC, IPSC			4 altered motifs			22 hits	TOM1L1	intronic
17	54919190	0.82	0.95	rs9914596	C	T	0.18	0.21	0.12	0.30			ESC, IPSC			4 altered motifs			22 hits	TOM1L1	intronic
17	54920514	0.85	0.96	rs9914836	C	G	0.17	0.19	0.09	0.29			5 tissues	BLD					23 hits	TOM1L1	intronic
17	54920807	0.84	0.96	rs9915913	G	T	0.33	0.22	0.12	0.30			6 tissues	ADRL					21 hits	TOM1L1	intronic
17	54920956	0.82	0.96	rs71361737	G	GAC	0.37	0.22	0.12	0.30			4 tissues			AP-1,Gfi1,YY1			23 hits	TOM1L1	intronic
17	54928355	0.85	0.97	rs12952253	A	G	0.05	0.18	0.23	0.30			ESDR			4 altered motifs			18 hits	TOM1L1	intronic
17	54928963	0.85	0.97	rs7209926	A	G	0.23	0.20	0.23	0.30			STRM			Foxj1,Nkx6-1		1 hit	24 hits	TOM1L1	intronic
17	54928978	0.85	0.97	rs7208123	G	A	0.06	0.18	0.23	0.30			STRM			Pou5f1,YY1			21 hits	TOM1L1	intronic
17	54929293	0.95	0.97	rs7214573	T	C	0.05	0.16	0.12	0.28			STRM, PANC			6 altered motifs			21 hits	TOM1L1	intronic
17	54929529	0.85	0.97	rs9916547	G	A	0.23	0.20	0.23	0.30			STRM, PANC			Ehf,Ets			24 hits	TOM1L1	intronic
17	54930710	0.96	0.98	rs35647022	C	T	0.05	0.16	0.09	0.28						E2F,Myc,Nrf1			21 hits	TOM1L1	intronic
17	54931492	0.86	0.98	rs8065361	A	T	0.23	0.20	0.23	0.30			GI			KAP1			24 hits	TOM1L1	intronic
17	54931919	0.87	0.98	rs72829484	G	T	0.06	0.18	0.23	0.30			GI			4 altered motifs			20 hits	TOM1L1	intronic
17	54932610	0.86	0.98	rs9912589	G	A	0.23	0.20	0.23	0.30			GI			4 altered motifs			23 hits	TOM1L1	intronic
17	54932737	0.86	0.98	rs12603899	T	C	0.23	0.20	0.23	0.30			GI			Nanog,Sin3Ak-20			23 hits	TOM1L1	intronic
17	54933309	0.96	0.98	rs12949538	C	T	0.05	0.16	0.12	0.28			ESDR, STRM, GI	GI		13 altered motifs		2 hits	22 hits	TOM1L1	intronic
17	54933394	0.86	0.98	rs9891704	A	G	0.23	0.20	0.23	0.30			ESDR, STRM, GI	GI,GI		Ets,Irf,TCF12			24 hits	TOM1L1	intronic
17	54934696	0.84	0.98	rs9900816	G	A	0.24	0.20	0.23	0.30			ESC, GI			Mef2		2 hits	24 hits	TOM1L1	intronic
17	54935566	0.87	0.99	rs9907961	C	G	0.24	0.20	0.23	0.30			ESC, GI	ESDR,GI,GI		Evi-1,NF-I,SIX5			24 hits	TOM1L1	intronic
17	54935928	0.86	0.97	rs9909096	G	T	0.24	0.20	0.23	0.30			GI			Irf			23 hits	TOM1L1	intronic
17	54935996	0.88	0.99	rs35465233	G	A	0.05	0.18	0.23	0.30			GI			Pax-4,ZEB1			20 hits	TOM1L1	intronic
17	54936006	0.88	0.99	rs34652394	C	T	0.05	0.18	0.22	0.30			GI			LUN-1,Pitx2			17 hits	TOM1L1	intronic
17	54936234	0.88	0.99	rs17745183	G	T	0.05	0.18	0.23	0.30			GI			TATA			21 hits	TOM1L1	intronic
17	54936619	0.97	0.99	rs17745189	A	G	0.05	0.16	0.12	0.28						Mef2,TATA			21 hits	TOM1L1	intronic
17	54938769	0.98	0.99	rs12937760	G	T	0.05	0.17	0.12	0.28									22 hits	TOM1L1	intronic
17	54938794	0.99	0.99	rs35297544	CTT	C	0.05	0.16	0.12	0.28						10 altered motifs			21 hits	TOM1L1	intronic
17	54940386	0.97	0.99	rs56175787	A	G	0.05	0.16	0.12	0.28						CEBPG,DMRT4			21 hits	TOM1L1	intronic
17	54940920	0.99	0.99	rs17817847	T	A	0.05	0.16	0.12	0.28						AP-3,Cdx			21 hits	TOM1L1	intronic
17	54944562	1	1	rs35097996	T	C	0.05	0.16	0.12	0.28			BLD, MUS			6 altered motifs			21 hits	TOM1L1	intronic
17	54948114	1	1	rs8066833	A	G	0.05	0.16	0.12	0.28			11 tissues	MUS		4 altered motifs			21 hits	TOM1L1	intronic
17	54948328	1	1	rs8066588	C	T	0.05	0.16	0.12	0.28			14 tissues	8 tissues		E2A,Spz1,ZEB1			22 hits	TOM1L1	intronic
17	54949462	0.99	1	rs71159274	TAAAAG	T	0.05	0.16	0.12	0.28			6 tissues			5 altered motifs			21 hits	TOM1L1	intronic
17	54951254	0.99	1	rs8073158	C	A	0.05	0.17	0.12	0.28			ESDR, GI, MUS			SRF			21 hits	TOM1L1	intronic
17	54951967	1	1	rs3087650	G	A	0.05	0.16	0.12	0.28						TEF-1		3 hits	22 hits	TOM1L1	intronic
17	54952865	1	1	rs12936860	G	A	0.05	0.16	0.12	0.28				BLD				6 hits	23 hits	TOM1L1	intronic
17	54953100	1	1	rs12937006	C	T	0.05	0.16	0.12	0.28						9 altered motifs			21 hits	TOM1L1	intronic
17	54957267	1	1	rs17817877	T	A	0.05	0.15	0.09	0.28			ESC, ESDR, IPSC			Ascl2,Rhox11,TAL1			24 hits	TOM1L1	intronic
17	54958067	1	1	rs8076984	T	C	0.05	0.16	0.12	0.28									22 hits	TOM1L1	intronic
17	54958133	0.95	1	rs35726440	GA	G	0.04	0.16	0.11	0.27						4 altered motifs			18 hits	TOM1L1	intronic
17	54959168	1	1	rs17745231	C	G	0.05	0.16	0.12	0.28						DMRT1,DMRT4			21 hits	TOM1L1	intronic
17	54959998	1	1	rs12938118	T	C	0.05	0.16	0.12	0.28						6 altered motifs			21 hits	TOM1L1	intronic
17	54961293	1	1	rs1802212	A	C	0.05	0.16	0.12	0.28			BLD						22 hits	TOM1L1	missense
17	54961384	1	1	rs17817901	A	G	0.05	0.16	0.12	0.28			BLD			Evi-1			21 hits	TOM1L1	3'-UTR
17	54964159	1	1	rs12150038	T	G	0.05	0.16	0.12	0.28						PLZF			21 hits	COX11	intronic
17	54964529	0.95	1	rs201764136	7-mer	T	0.04	0.16	0.12	0.27						5 altered motifs			22 hits	COX11	intronic
17	54964531	0.96	0.99	rs141544971	8-mer	C	0.04	0.16	0.12	0.27						4 altered motifs			2 hits	COX11	intronic
17	54964604	1	1	rs12951542	T	C	0.05	0.16	0.12	0.28						Pou5f1			21 hits	COX11	intronic
17	54966443	0.99	1	rs72831295	T	C	0.05	0.17	0.12	0.28		11 tissues	14 tissues			4 altered motifs			21 hits	COX11	intronic
17	54969776	0.83	0.94	rs9915183	G	A	0.29	0.17	0.09	0.27		20 tissues	10 tissues	7 tissues	GATA2,GATA1	Myb,SIX5			28 hits	STXBP4	intronic
17	54970138	0.83	0.94	rs7222197	G	A	0.29	0.17	0.09	0.27		16 tissues	14 tissues	OVRY,PANC	GATA2,GATA1,GATA3			2 hits	29 hits	STXBP4	intronic
17	54970958	0.84	0.94	rs17745344	A	G	0.03	0.14	0.09	0.26		BLD, LIV	5 tissues			4 altered motifs			20 hits	STXBP4	intronic
17	54971081	0.81	0.92	rs9895808	C	G	0.29	0.17	0.09	0.27		BLD, LIV	6 tissues			5 altered motifs			27 hits	STXBP4	intronic
17	54971108	0.81	0.92	rs9897447	T	C	0.29	0.17	0.09	0.27		BLD, LIV	6 tissues			4 altered motifs			27 hits	STXBP4	intronic
17	54971181	0.81	0.92	rs9896044	C	G	0.28	0.17	0.09	0.27		BLD, LIV	6 tissues	OVRY,BLD		16 altered motifs			27 hits	STXBP4	intronic
17	54971563	0.81	0.92	rs9902687	G	A	0.29	0.17	0.09	0.27			4 tissues	BLD		Isl2,UF1H3BETA			28 hits	STXBP4	intronic
17	54972508	0.81	0.92	rs8080491	T	A	0.30	0.17	0.09	0.27			BLD, LIV		PU1	14 altered motifs			27 hits	STXBP4	intronic
17	54972626	0.81	0.92	rs6504948	T	C	0.29	0.17	0.09	0.27			BLD, LIV	BLD	PU1	4 altered motifs			28 hits	STXBP4	intronic
17	54972772	0.81	0.92	rs6504949	T	G	0.29	0.17	0.09	0.27			4 tissues	BLD	4 bound proteins	Foxj1			28 hits	STXBP4	intronic
17	54975207	0.8	0.92	rs35051208	C	T	0.05	0.15	0.09	0.27						4 altered motifs			20 hits	STXBP4	intronic
17	54976018	0.81	0.92	rs8078550	T	G	0.29	0.17	0.09	0.27			BLD, LIV			14 altered motifs			28 hits	STXBP4	intronic
17	54977006	0.81	0.92	rs9914732	C	G	0.29	0.17	0.09	0.27						BDP1,Mef2			27 hits	STXBP4	intronic
17	54977136	0.81	0.92	rs9916642	T	C	0.29	0.17	0.09	0.27						Nr2f2,TCF12			28 hits	STXBP4	intronic
17	54977336	0.81	0.92	rs9915832	G	A	0.29	0.17	0.09	0.27				LNG		Cart1			28 hits	STXBP4	intronic
17	54977388	0.81	0.92	rs9893306	A	T	0.29	0.17	0.09	0.27				LNG		ERalpha-a,RORalpha1			28 hits	STXBP4	intronic
17	54977885	0.81	0.92	rs9894529	A	T	0.28	0.17	0.09	0.27			BLD		PU1	12 altered motifs			26 hits	STXBP4	intronic
17	54979110	0.81	0.92	rs6504950	G	A	0.30	0.17	0.09	0.27			6 tissues	10 tissues		Bbx,Ets	1 hit		27 hits	STXBP4	intronic
17	54979614	0.81	0.92	rs6504951	A	G	0.29	0.17	0.09	0.27			6 tissues	IPSC,SKIN		Hand1,Rad21,THAP1			27 hits	STXBP4	intronic
17	54980030	0.81	0.92	rs71300611	C	CTA	0.30	0.17	0.09	0.27			STRM			6 altered motifs			27 hits	STXBP4	intronic
17	54980386	0.81	0.92	rs9903146	C	T	0.29	0.17	0.09	0.27						BAF155,SIX5,Znf143			27 hits	STXBP4	intronic
17	54980403	0.81	0.92	rs9902950	A	T	0.28	0.17	0.09	0.27						Pou2f2,Sox			27 hits	STXBP4	intronic
17	54980504	0.81	0.92	rs9903220	A	G	0.29	0.17	0.09	0.27				4 tissues		Egr-1,GR			28 hits	STXBP4	intronic
17	54980532	0.81	0.92	rs9903444	C	T	0.30	0.17	0.09	0.27				ESDR,LNG,BLD					27 hits	STXBP4	intronic
17	54980553	0.81	0.92	rs9903825	G	A	0.29	0.17	0.09	0.27				ESDR,LNG,BLD		Alx4,E2F,PLZF			28 hits	STXBP4	intronic
17	54981315	0.81	0.92	rs28558726	A	G	0.29	0.17	0.09	0.27						Mrg1::Hoxa9			27 hits	STXBP4	intronic
17	54981446	0.81	0.92	rs16955471	G	T	0.29	0.17	0.09	0.27						Foxj1,Mrg1::Hoxa9,p300			27 hits	STXBP4	intronic
17	54981646	0.82	0.93	rs7221223	A	G	0.06	0.15	0.09	0.26			FAT, MUS			4 altered motifs			21 hits	STXBP4	intronic
17	54982672	0.81	0.92	rs9891865	C	T	0.29	0.17	0.09	0.27		MUS	4 tissues	16 tissues	GR	4 altered motifs			27 hits	STXBP4	intronic
17	54982853	0.8	0.92	rs9892173	C	T	0.29	0.18	0.09	0.27			5 tissues	MUS		GATA,Pax-4			27 hits	STXBP4	intronic
17	54982968	0.8	0.92	rs9892976	G	A	0.29	0.17	0.09	0.26			5 tissues	MUS		BDP1,E2F,HNF4			27 hits	STXBP4	intronic
17	54983714	0.81	0.92	rs1990674	G	A	0.29	0.17	0.09	0.27			FAT, STRM, MUS			4 altered motifs			28 hits	STXBP4	intronic
17	54984261	0.81	0.92	rs9902718	T	C	0.29	0.17	0.09	0.27						Crx,Mrg1::Hoxa9,Obox3		1 hit	27 hits	STXBP4	intronic
17	54985394	0.82	0.93	rs17818028	C	G	0.05	0.15	0.09	0.26		ESC	5 tissues					1 hit	20 hits	STXBP4	intronic
17	54985542	0.81	0.92	rs10468513	C	A	0.29	0.17	0.09	0.27						19 altered motifs			27 hits	STXBP4	intronic
17	54987189	0.81	0.92	rs56348638	C	T	0.29	0.17	0.09	0.27				BRST		4 altered motifs			27 hits	STXBP4	intronic
17	54988446	0.81	0.92	rs7219874	C	T	0.29	0.17	0.09	0.27				LNG		10 altered motifs			27 hits	STXBP4	intronic
17	54990322	0.82	0.93	rs12940106	A	G	0.05	0.15	0.09	0.26		GI	ESDR	4 tissues		Maf			19 hits	STXBP4	intronic
17	54990632	0.81	0.92	rs8082471	T	A	0.29	0.17	0.09	0.27						5 altered motifs			28 hits	STXBP4	intronic

Query SNP: rs2267372 and variants with r^2 >= 0.8

chr	pos (hg38)	LD (r^2)	LD (D')	variant	Ref	Alt	AFR freq	AMR freq	ASN freq	EUR freq	SiPhy cons	Promoter histone marks	Enhancer histone marks	DNAse	Proteins bound	Motifs changed	NHGRI/EBI GWAS hits	GRASP QTL hits	Selected eQTL hits	GENCODE genes	dbSNP func annot
22	38176519	0.83	-0.96	rs133015	C	G	0.47	0.51	0.25	0.41		SKIN, MUS, BLD	20 tissues	36 tissues	8 bound proteins	GR,SIX5			11 hits	PLA2G6	intronic
22	38179491	0.83	-0.96	rs133027	CT	C	0.50	0.51	0.25	0.41			17 tissues	LIV		BATF,Sin3Ak-20,Zfx				PLA2G6	intronic
22	38181508	0.84	0.97	rs2277844	G	A	0.45	0.49	0.75	0.59		24 tissues		34 tissues	POL2,ZNF263	RXRA			11 hits	PLA2G6	intronic
22	38184364	0.81	0.96	rs3761444	G	A	0.36	0.50	0.63	0.59			ESC, PLCNT, BLD			9 altered motifs			14 hits	PLA2G6	
22	38184910	0.92	0.97	rs13056506	G	T	0.45	0.52	0.75	0.62			6 tissues	5 tissues		BRCA1,TBX5			6 hits	PLA2G6	
22	38185853	0.89	0.97	rs200173815	T	TTTC	0.44	0.51	0.74	0.61			PLCNT, MUS			10 altered motifs			6 hits	PLA2G6	
22	38185854	0.89	0.97	rs201373285	T	TTC	0.44	0.51	0.74	0.61			PLCNT, MUS			10 altered motifs			6 hits	PLA2G6	
22	38186490	0.81	0.96	rs5750558	G	A	0.36	0.50	0.63	0.59			5 tissues	6 tissues		Mef2			15 hits	PLA2G6	
22	38196960	0.84	0.98	rs2413505	G	A	0.25	0.49	0.63	0.58		SKIN, GI, LIV	10 tissues	15 tissues		AP-4			11 hits	4.9kb 5' of PLA2G6	
22	38197421	0.84	0.99	rs2413507	A	G	0.36	0.49	0.63	0.58						Crx,LUN-1,Pitx2			12 hits	4.5kb 5' of MAFF	
22	38198661	0.95	0.98	rs2899297	G	A	0.35	0.51	0.75	0.61		IPSC	4 tissues	THYM		4 altered motifs		1 hit	4 hits	3.2kb 5' of MAFF	
22	38199233	0.95	0.98	rs5756968	C	T	0.36	0.51	0.75	0.61			BLD, LIV	BLD,BLD		AP-3,Arid5a,Pax-5		1 hit	8 hits	2.6kb 5' of MAFF	
22	38199253	0.93	0.98	rs5750561	A	T	0.35	0.50	0.75	0.61			BLD, LIV	BLD,BLD		HNF1			4 hits	2.6kb 5' of MAFF	
22	38199404	0.95	0.98	rs3761445	G	A	0.36	0.51	0.75	0.61			BLD	12 tissues	7 bound proteins	4 altered motifs		2 hits	8 hits	2.5kb 5' of MAFF	
22	38199532	0.84	0.98	rs3761447	G	A	0.26	0.49	0.63	0.59			BLD		SP1,YY1,POL2	NF-E2			14 hits	2.3kb 5' of MAFF	
22	38199608	0.82	0.96	rs3761449	C	T	0.33	0.48	0.73	0.59			BLD			SZF1-1,Spz1,ZBTB33			4 hits	2.3kb 5' of MAFF	
22	38200093	0.84	0.98	rs9607517	G	A	0.25	0.49	0.63	0.59			BLD	BLD,OVRY,MUS		RFX5			13 hits	1.8kb 5' of MAFF	
22	38202227	1	1	rs2267372	A	G	0.56	0.53	0.74	0.62		24 tissues	BRN	35 tissues	5 bound proteins	6 altered motifs			6 hits	MAFF	5'-UTR
22	38203357	0.9	0.98	rs4821764	G	A	0.57	0.53	0.67	0.60		23 tissues	BRN	46 tissues	4 bound proteins				7 hits	MAFF	intronic
22	38203760	0.9	0.98	rs4820323	C	G	0.58	0.53	0.66	0.60		22 tissues	11 tissues	9 tissues	HNF4A	4 altered motifs			7 hits	MAFF	intronic
22	38203850	0.9	0.98	rs4820324	G	C	0.57	0.53	0.66	0.60		17 tissues	16 tissues	8 tissues	HNF4A	Pax-5,Smad			7 hits	MAFF	intronic
22	38203971	0.9	0.98	rs4820325	G	A	0.57	0.53	0.66	0.60		17 tissues	16 tissues	16 tissues	GATA2	CHD2,Nr2f2			7 hits	MAFF	intronic
22	38204535	0.9	0.98	rs2267373	C	T	0.58	0.53	0.66	0.60		10 tissues	17 tissues	8 tissues		4 altered motifs			11 hits	MAFF	intronic
22	38204588	0.8	0.98	rs2267374	A	T	0.19	0.49	0.52	0.57		10 tissues	17 tissues	7 tissues		9 altered motifs			12 hits	MAFF	intronic
22	38205224	0.9	0.98	rs2267375	G	T	0.58	0.53	0.66	0.60		10 tissues	17 tissues	9 tissues		6 altered motifs			11 hits	MAFF	intronic

Query SNP: rs2541243 and variants with r^2 >= 0.8

chr	pos (hg38)	LD (r^2)	LD (D')	variant	Ref	Alt	AFR freq	AMR freq	ASN freq	EUR freq	SiPhy cons	Promoter histone marks	Enhancer histone marks	DNAse	Proteins bound	Motifs changed	NHGRI/EBI GWAS hits	GRASP QTL hits	Selected eQTL hits	GENCODE genes	dbSNP func annot
17	54998066	0.8	0.91	rs4794551	A	G	0.62	0.80	0.74	0.72						Ik-2,NF-AT			18 hits	STXBP4	intronic
17	54999438	0.84	0.99	rs1156287	G	A	0.95	0.85	0.90	0.74						4 altered motifs		3 hits	18 hits	STXBP4	missense
17	54999625	0.83	0.99	rs11658717	G	A	0.71	0.82	0.90	0.74						Mef2			24 hits	STXBP4	intronic
17	55005761	0.83	0.99	rs11079143	A	G	0.71	0.83	0.90	0.74			ESC	28 tissues	CTCF,RAD21	Dlx3,Foxa,Hmx			24 hits	STXBP4	intronic
17	55011772	0.82	0.97	rs2787476	T	C	0.94	0.84	0.89	0.73						Irf,PRDM1			16 hits	STXBP4	intronic
17	55011778	0.85	1	rs2787477	G	C	0.95	0.85	0.90	0.74						ERalpha-a			16 hits	STXBP4	intronic
17	55012326	0.84	1	rs2541242	T	C	0.95	0.85	0.90	0.74						6 altered motifs			15 hits	STXBP4	intronic
17	55013820	0.83	0.99	rs2787494	G	C	0.94	0.85	0.90	0.74						CTCF,ZEB1			16 hits	STXBP4	intronic
17	55013905	1	1	rs2541243	A	G	0.92	0.83	0.89	0.71									14 hits	STXBP4	intronic
17	55016604	0.82	0.92	rs2787501	G	C	0.62	0.79	0.74	0.72				ESDR		7 altered motifs		2 hits	18 hits	STXBP4	intronic
17	55023839	0.85	1	rs2541245	G	A	0.95	0.85	0.90	0.74				4 tissues		4 altered motifs			18 hits	STXBP4	intronic
17	55030071	0.85	1	rs2787505	T	C	0.95	0.85	0.90	0.74				BRN,BRN					18 hits	STXBP4	intronic
17	55033760	0.8	0.91	rs12449538	T	C	0.61	0.79	0.74	0.72						MIF-1			19 hits	STXBP4	intronic
17	55034022	0.83	0.99	rs1484776	A	C	0.75	0.82	0.90	0.74						4 altered motifs			24 hits	STXBP4	intronic
17	55038058	0.83	0.99	rs2628309	G	A	0.94	0.84	0.90	0.74			ESDR	9 tissues	NFKB,PBX3				17 hits	STXBP4	intronic
17	55046212	0.83	0.99	rs2628308	C	G	0.71	0.82	0.90	0.74						GR,Ik-1,STAT			23 hits	STXBP4	intronic
17	55050563	0.84	0.99	rs2787507	C	T	0.95	0.85	0.90	0.74						5 altered motifs			17 hits	STXBP4	intronic
17	55055050	0.84	0.99	rs9889687	G	A	0.95	0.85	0.90	0.74			BLD			4 altered motifs			17 hits	STXBP4	intronic
17	55060145	0.84	0.99	rs17745534	A	G	0.94	0.85	0.90	0.74			BRST			4 altered motifs			17 hits	STXBP4	intronic
17	55060613	0.84	0.99	rs9303363	A	G	0.95	0.85	0.90	0.74			BLD	BLD		5 altered motifs		2 hits	20 hits	STXBP4	intronic
17	55063066	0.83	0.99	rs2628305	A	C	0.75	0.83	0.90	0.74			6 tissues	11 tissues		5 altered motifs		2 hits	22 hits	STXBP4	intronic
17	55079828	0.83	0.99	rs2628304	G	A	0.71	0.82	0.90	0.74						4 altered motifs			23 hits	STXBP4	intronic
17	55081912	0.82	0.99	rs2628301	T	C	0.95	0.85	0.91	0.75						5 altered motifs			16 hits	STXBP4	intronic
17	55082471	0.81	0.99	rs2541236	T	G	0.71	0.83	0.91	0.74						8 altered motifs			21 hits	STXBP4	intronic
17	55086676	0.82	0.97	rs2541237	C	G	0.93	0.85	0.90	0.74		BLD	10 tissues	13 tissues	9 bound proteins	AIRE,Roaz			18 hits	STXBP4	intronic
17	55089134	0.83	0.99	rs2628296	T	A	0.69	0.82	0.90	0.74			BLD	BLD		5 altered motifs			23 hits	STXBP4	intronic
17	55089290	0.84	0.99	rs1484769	G	A	0.93	0.85	0.90	0.74			BLD					2 hits	19 hits	STXBP4	intronic
17	55092445	0.8	0.91	rs2787500	G	T	0.63	0.79	0.73	0.72			BLD			Pdx1			17 hits	STXBP4	intronic

Query SNP: rs2974935 and variants with r^2 >= 0.8

chr	pos (hg38)	LD (r^2)	LD (D')	variant	Ref	Alt	AFR freq	AMR freq	ASN freq	EUR freq	SiPhy cons	Promoter histone marks	Enhancer histone marks	DNAse	Proteins bound	Motifs changed	NHGRI/EBI GWAS hits	GRASP QTL hits	Selected eQTL hits	GENCODE genes	dbSNP func annot
1	155192276	0.85	0.99	rs4072037	C	T	0.66	0.66	0.84	0.59		6 tissues	7 tissues	GI,PANC,CRVX	POL2	DMRT5,Myb,TAL1	2 hits	5 hits	75 hits	MUC1	synonymous
1	155194689	0.91	-0.99	rs12411216	C	A	0.38	0.37	0.18	0.43		23 tissues	PLCNT, GI, SPLN	49 tissues	20 bound proteins	8 altered motifs			69 hits	487bp 5' of MIR92B	
1	155199058	0.91	0.99	rs2974937	C	T	0.62	0.64	0.82	0.57			ESDR			4 altered motifs			70 hits	THBS3	intronic
1	155202588	0.92	0.99	rs2066981	G	A	0.62	0.64	0.83	0.57			IPSC	LNG		CTCF,RXRA,YY1		2 hits	75 hits	THBS3	intronic
1	155205599	0.92	0.99	rs370545	A	G	0.62	0.64	0.83	0.57			7 tissues			5 altered motifs			68 hits	THBS3	intronic
1	155206101	0.92	0.98	rs914615	A	G	0.62	0.64	0.83	0.56			7 tissues	IPSC,BLD,MUS	ZNF263	Rad21		2 hits	73 hits	THBS3	intronic
1	155212052	1	1	rs2974935	G	T	0.62	0.62	0.82	0.55		BLD	STRM, BLD			Glis2,Smad3			65 hits	MTX1	intronic
1	155212373	1	1	rs2075570	C	T	0.62	0.61	0.82	0.55		BLD	STRM, GI, BLD			Egr-1,GLI,STAT			64 hits	MTX1	intronic
1	155216938	0.99	1	rs28445596	T	C	0.62	0.61	0.82	0.55			ESDR, BLD, PLCNT	PLCNT		6 altered motifs		1 hit	64 hits	3.1kb 3' of MTX1	
1	155220463	0.99	1	rs2990220	T	A	0.61	0.62	0.82	0.55			4 tissues	4 tissues					64 hits	6.6kb 3' of MTX1	intronic
1	155223741	0.99	1	rs497829	C	G	0.63	0.62	0.81	0.55		CRVX	6 tissues						64 hits	9.9kb 3' of MTX1	intronic
1	155225189	0.99	1	rs2049805	T	C	0.62	0.62	0.81	0.55		6 tissues	18 tissues	21 tissues		HDAC2,Hoxa5	1 hit	11 hits	69 hits	9.3kb 3' of GBA	intronic
1	155225424	0.97	1	rs2974931	C	G	0.62	0.61	0.81	0.54		SKIN, BLD	19 tissues			Irf,SP1			63 hits	9kb 3' of GBA	intronic
1	155226926	0.99	1	rs2974930	A	G	0.63	0.62	0.81	0.55		22 tissues	SPLN	34 tissues	11 bound proteins	Nr2f2			61 hits	7.5kb 3' of GBA	intronic
1	155227477	0.98	1	rs2974929	T	C	0.62	0.60	0.84	0.55		23 tissues	SPLN	53 tissues	33 bound proteins	4 altered motifs			66 hits	7kb 3' of GBA	
1	155227671	0.97	1	rs2990245	C	T	0.62	0.61	0.82	0.54		23 tissues	SPLN	53 tissues	21 bound proteins	GR		6 hits	66 hits	6.8kb 3' of GBA	

Query SNP: rs2981428 and variants with r^2 >= 0.8

chr	pos (hg38)	LD (r^2)	LD (D')	variant	Ref	Alt	AFR freq	AMR freq	ASN freq	EUR freq	SiPhy cons	Promoter histone marks	Enhancer histone marks	DNAse	Proteins bound	Motifs changed	NHGRI/EBI GWAS hits	GRASP QTL hits	Selected eQTL hits	GENCODE genes	dbSNP func annot
10	121569915	1	1	rs2981428	T	G	0.04	0.40	0.56	0.54			5 tissues			Myc			1 hit	FGFR2	intronic

Query SNP: rs3104793 and variants with r^2 >= 0.8

chr	pos (hg38)	LD (r^2)	LD (D')	variant	Ref	Alt	AFR freq	AMR freq	ASN freq	EUR freq	SiPhy cons	Promoter histone marks	Enhancer histone marks	DNAse	Proteins bound	Motifs changed	NHGRI/EBI GWAS hits	GRASP QTL hits	Selected eQTL hits	GENCODE genes	dbSNP func annot
16	52590623	0.97	0.98	rs3104766	C	A	0.36	0.54	0.81	0.43						10 altered motifs			1 hit	RP11-297L17.1	intronic
16	52590826	0.99	1	rs3104767	G	T	0.67	0.56	0.81	0.43			GI			Foxj2,Pou5f1,Sox	1 hit			RP11-297L17.1	intronic
16	52591288	0.98	1	rs3112626	A	G	0.47	0.53	0.81	0.43			GI, PANC						1 hit	RP11-297L17.1	intronic
16	52591640	1	1	rs3112625	A	G	0.60	0.56	0.81	0.43			4 tissues	4 tissues		HNF6,Nobox			1 hit	RP11-297L17.1	intronic
16	52591701	0.94	1	rs12920540	C	A	0.20	0.46	0.44	0.42			SKIN, GI, PANC	5 tissues						RP11-297L17.1	intronic
16	52592484	0.96	0.98	rs3104768	G	A	0.47	0.54	0.81	0.43			4 tissues	4 tissues		CIZ			1 hit	RP11-297L17.1	intronic
16	52592639	0.98	1	rs3112624	A	G	0.47	0.54	0.81	0.43			5 tissues	SKIN,LNG,MUS					1 hit	RP11-297L17.1	intronic
16	52593319	0.97	1	rs3104769	C	T	0.47	0.54	0.81	0.43						Foxj2,HMG-IY			1 hit	RP11-297L17.1	intronic
16	52593614	0.98	1	rs3112623	A	G	0.47	0.54	0.81	0.43						DMRT7,Evi-1,Mef2			1 hit	RP11-297L17.1	intronic
16	52594506	0.98	-1	rs11860998	C	G	0.52	0.46	0.19	0.57						AhR::Arnt,Arnt,Pitx2			1 hit	RP11-297L17.1	intronic
16	52594581	0.94	1	rs7192828	C	G	0.20	0.46	0.44	0.42										RP11-297L17.1	intronic
16	52594625	0.98	-1	rs11860769	G	A	0.52	0.46	0.19	0.57						Zfx			1 hit	RP11-297L17.1	intronic
16	52594652	0.98	-1	rs11861036	C	T	0.52	0.46	0.19	0.57						E2A,TBX5,ZEB1			1 hit	RP11-297L17.1	intronic
16	52595414	0.98	1	rs3104772	G	A	0.48	0.54	0.81	0.43						Cdx2,HMG-IY,THAP1			1 hit	RP11-297L17.1	intronic
16	52595524	0.98	1	rs3104773	G	A	0.47	0.54	0.81	0.43						Rad21,Smad			1 hit	RP11-297L17.1	intronic
16	52595591	0.98	1	rs3112622	T	C	0.48	0.54	0.81	0.43									1 hit	RP11-297L17.1	intronic
16	52595790	0.98	1	rs3104774	G	A	0.48	0.54	0.81	0.43									1 hit	RP11-297L17.1	intronic
16	52595915	0.98	1	rs3104775	G	A	0.48	0.54	0.81	0.43						Ets			1 hit	RP11-297L17.1	intronic
16	52596233	0.98	1	rs7203671	G	C	0.48	0.54	0.81	0.43						5 altered motifs			1 hit	RP11-297L17.1	intronic
16	52596437	0.99	1	rs3112621	A	G	0.60	0.56	0.81	0.44						Hic1,SEF-1			1 hit	RP11-297L17.1	intronic
16	52596984	0.98	0.99	rs3112620	A	T	0.33	0.53	0.81	0.43			GI			7 altered motifs			1 hit	RP11-297L17.1	intronic
16	52597274	0.98	0.99	rs3112618	C	G	0.48	0.54	0.81	0.43			GI, LIV	4 tissues		Bcl6b			1 hit	RP11-297L17.1	intronic
16	52597550	0.98	0.99	rs3104776	A	G	0.48	0.54	0.81	0.43			GI, LIV	LIV		13 altered motifs			1 hit	RP11-297L17.1	intronic
16	52597805	0.98	0.99	rs3112617	T	G	0.48	0.54	0.81	0.43			ESDR, LIV, GI	16 tissues	8 bound proteins	6 altered motifs			1 hit	RP11-297L17.1	intronic
16	52597835	0.98	0.99	rs3112616	T	G	0.48	0.54	0.81	0.43			ESDR, LIV, GI	16 tissues	9 bound proteins	CDP			1 hit	RP11-297L17.1	intronic
16	52598052	0.98	0.99	rs11075551	A	G	0.48	0.54	0.81	0.43		GI, LIV	6 tissues	5 tissues	HDAC2,TCF4,GATA3				1 hit	RP11-297L17.1	intronic
16	52598438	0.98	0.99	rs3112615	A	C	0.48	0.54	0.81	0.43			4 tissues	SKIN	FOXA1	7 altered motifs			1 hit	RP11-297L17.1	intronic
16	52598719	0.98	1	rs3104777	C	T	0.43	0.53	0.81	0.43			SKIN, LIV	ADRL		Egr-1,VDR			1 hit	RP11-297L17.1	intronic
16	52598818	1	-1	rs45544231	G	C	0.09	0.41	0.19	0.57			SKIN, LIV							RP11-297L17.1	intronic
16	52598985	0.98	-1	rs45500599	A	G	0.57	0.47	0.19	0.57			SKIN, LIV						1 hit	RP11-297L17.1	intronic
16	52599032	0.94	1	rs12929797	C	T	0.20	0.46	0.45	0.42			SKIN, LIV			Sox,THAP1				RP11-297L17.1	intronic
16	52599740	0.98	1	rs3104778	A	G	0.48	0.54	0.81	0.43			LIV	BRN		4 altered motifs			1 hit	RP11-297L17.1	intronic
16	52599745	0.98	1	rs3104779	A	G	0.48	0.54	0.81	0.43			LIV	BRN		DMRT2,E2A,RP58			1 hit	RP11-297L17.1	intronic
		0.97	0.99	rs79346458	C	T	0.43	0.53	0.81	0.43			GI, LIV			Gcm1				RP11-297L17.1	intronic
16	52600088	0.99	1	rs3104781	C	T	0.55	0.55	0.81	0.43			GI, LIV							RP11-297L17.1	intronic
16	52600251	1	1	rs3112613	G	A	0.60	0.56	0.81	0.43			GI, LIV			Irf,STAT			1 hit	RP11-297L17.1	intronic
16	52601252	0.98	1	rs3112612	G	A	0.47	0.54	0.81	0.43				SKIN		Foxl1	1 hit		1 hit	RP11-297L17.1	intronic
16	52602265	0.99	1	rs3104782	A	G	0.60	0.56	0.81	0.44			LIV			PU.1,Pax-5			1 hit	RP11-297L17.1	intronic
16	52602330	0.99	1	rs3104783	C	A	0.60	0.56	0.81	0.44			GI, LIV			Hoxa9,Pou1f1			1 hit	RP11-297L17.1	intronic
16	52602453	0.99	1	rs3104784	A	C	0.67	0.56	0.81	0.44			GI, LIV			FAC1,Foxp1,NF-I			1 hit	RP11-297L17.1	intronic
16	52603451	0.98	0.99	rs3104786	A	G	0.47	0.54	0.81	0.43			GI, LIV			Zbtb3			1 hit	RP11-297L17.1	intronic
16	52603523	0.94	0.99	rs4578643	G	T	0.20	0.46	0.44	0.42			GI, LIV			Dlx3				RP11-297L17.1	intronic
16	52603631	0.98	0.99	rs3104787	T	C	0.47	0.54	0.81	0.43			GI, LIV			6 altered motifs			1 hit	RP11-297L17.1	intronic
16	52603868	0.94	0.99	rs12597685	T	C	0.20	0.46	0.44	0.42			GI, LIV			Zfp105				RP11-297L17.1	intronic
16	52604591	0.98	0.99	rs3104788	T	C	0.53	0.54	0.81	0.43			LIV	LIV		GR,MIF-1,RFX5		1 hit	1 hit	RP11-297L17.1	intronic
16	52604636	0.97	0.98	rs3104789	T	C	0.47	0.54	0.81	0.43			LIV	LIV		4 altered motifs			1 hit	RP11-297L17.1	intronic
16	52604751	0.97	0.99	rs3104790	C	T	0.43	0.53	0.81	0.43			LIV	LIV		Pou1f1			1 hit	RP11-297L17.1	intronic
16	52604847	0.94	1	rs4290467	A	G	0.20	0.46	0.44	0.42			LIV			TAL1				RP11-297L17.1	intronic
16	52605050	0.98	1	rs3104791	G	A	0.42	0.53	0.81	0.43									1 hit	RP11-297L17.1	intronic
16	52605322	0.94	1	rs4238749	C	A	0.20	0.46	0.45	0.42						Gcm1,Rad21				RP11-297L17.1	intronic
16	52605324	0.94	1	rs4238750	C	T	0.34	0.47	0.45	0.42						4 altered motifs				RP11-297L17.1	intronic
16	52605843	0.94	1	rs1112135	C	T	0.34	0.47	0.45	0.42										RP11-297L17.1	intronic
16	52607274	1	1	rs3104793	T	C	0.67	0.56	0.81	0.43						CEBPB,Rad21			1 hit	RP11-297L17.1	
16	52608465	0.99	1	rs3104796	A	G	0.66	0.56	0.81	0.43			GI			ATF3,Klf4			1 hit	RP11-297L17.1	
16	52610012	0.94	1	rs8046994	C	T	0.36	0.47	0.44	0.42			GI							RP11-297L17.1	
16	52610538	0.99	1	rs3104797	A	C	0.67	0.56	0.81	0.44			GI, LIV			Foxp1			1 hit	RP11-297L17.1	
16	52610983	0.94	0.99	rs3104798	C	G	0.66	0.56	0.82	0.42			GI, LIV			11 altered motifs				RP11-297L17.1	
16	52612464	0.81	0.98	rs3104800	G	A	0.22	0.47	0.58	0.39		5 tissues	ESDR, GI, PANC	6 tissues		GATA,Gfi1				RP11-297L17.1	
16	52613258	0.81	0.98	rs3112611	G	T	0.22	0.47	0.58	0.39			GI, LIV			DMRT5				RP11-297L17.1	
16	52614642	0.81	0.98	rs3112610	G	A	0.22	0.47	0.59	0.39						Arid5a,Pou1f1				RP11-297L17.1	
16	52614819	0.81	0.98	rs3112609	C	G	0.22	0.47	0.58	0.39										RP11-297L17.1	

Query SNP: rs4973758 and variants with r^2 >= 0.8

chr	pos (hg38)	LD (r^2)	LD (D')	variant	Ref	Alt	AFR freq	AMR freq	ASN freq	EUR freq	SiPhy cons	Promoter histone marks	Enhancer histone marks	DNAse	Proteins bound	Motifs changed	NHGRI/EBI GWAS hits	GRASP QTL hits	Selected eQTL hits	GENCODE genes	dbSNP func annot
3	27183958	0.86	0.94	rs62255215	C	T	0.11	0.32	0.09	0.27						TEF-1,Zbtb12			1 hit	NEK10	
3	27185883	0.86	0.94	rs62255216	T	C	0.11	0.32	0.09	0.27			FAT, BRN	BRN	CTCF	DMRT1,Pou3f2,p300			1 hit	NEK10	
3	27186065	0.86	0.94	rs11717372	G	A	0.11	0.32	0.09	0.27			FAT, BRN	BRN		5 altered motifs			2 hits	NEK10	
3	27186088	0.86	0.94	rs11717314	C	T	0.11	0.32	0.09	0.27			FAT, BRN	BRN	CEBPB				2 hits	NEK10	
3	27186519	0.86	0.94	rs10510596	G	A	0.11	0.32	0.09	0.27									1 hit	NEK10	
3	27186863	0.86	0.94	rs62255218	T	C	0.11	0.32	0.09	0.27						RREB-1			1 hit	NEK10	
3	27187399	0.86	0.94	rs62255219	G	T	0.11	0.32	0.09	0.27									1 hit	NEK10	
3	27187460	0.86	0.94	rs62255220	T	G	0.11	0.32	0.09	0.27						Pou2f2,RORalpha1,RXRA			1 hit	NEK10	
3	27190228	0.88	0.95	rs11928525	T	C	0.11	0.32	0.09	0.27			SKIN			Hsf			2 hits	NEK10	
3	27190562	0.88	0.95	rs79520262	G	A	0.11	0.32	0.09	0.27						4 altered motifs			1 hit	NEK10	
3	27190736	0.88	0.95	rs17679850	T	C	0.11	0.32	0.09	0.27						Mef2			1 hit	NEK10	
3	27191246	0.88	0.95	rs62255252	C	G	0.11	0.32	0.09	0.27			GI		RAD21				1 hit	NEK10	
3	27191459	0.88	0.95	rs11929690	T	G	0.11	0.32	0.09	0.27			GI		RAD21	E2A,RP58,ZEB1			1 hit	NEK10	
3	27191885	0.88	0.95	rs11926580	G	T	0.11	0.32	0.09	0.27						4 altered motifs			2 hits	NEK10	
3	27192007	0.88	0.95	rs11926556	C	A	0.12	0.33	0.09	0.27						GR,Pou2f2			2 hits	NEK10	
3	27193477	0.88	0.95	rs62255253	T	A	0.11	0.32	0.09	0.27			4 tissues			Ets,NF-I			1 hit	NEK10	
3	27194154	0.86	0.93	rs11928814	C	G	0.11	0.32	0.09	0.27			6 tissues	CRVX,BRN,LNG	STAT1				1 hit	NEK10	
3	27194325	0.88	0.95	rs11928858	C	T	0.11	0.32	0.09	0.27			6 tissues	CRVX,MUS,LNG	JUND,STAT1	NF-kappaB,Spz1			1 hit	NEK10	
3	27194449	0.88	0.95	rs11928916	C	A	0.11	0.32	0.09	0.27			6 tissues	5 tissues	JUND	Gfi1			2 hits	NEK10	
3	27195243	0.85	0.95	rs12485426	G	T	0.11	0.32	0.09	0.28			8 tissues	11 tissues	CJUN,JUND,P300	NF-I			1 hit	NEK10	
3	27195589	0.88	0.95	rs17628832	G	T	0.11	0.32	0.09	0.27			7 tissues			Evi-1			1 hit	NEK10	
3	27195899	0.88	0.95	rs62255256	A	T	0.11	0.32	0.09	0.27			6 tissues			5 altered motifs			1 hit	NEK10	
3	27195944	0.88	0.95	rs62255257	C	A	0.11	0.32	0.09	0.27			KID, CRVX, BONE			GATA,RXRA			1 hit	NEK10	
3	27196079	0.85	0.93	rs79729806	T	C	0.11	0.32	0.09	0.27			KID, CRVX, BONE			CTCF			1 hit	NEK10	
3	27196164	0.88	0.95	rs62255258	C	T	0.11	0.32	0.09	0.27			ESDR			CEBPB,Foxp3,Smad3			1 hit	NEK10	
3	27197630	0.82	0.94	rs13076466	C	T	0.11	0.33	0.09	0.28						BATF,Irf,ZBTB33			1 hit	NEK10	
3	27198480	0.82	0.94	rs13078105	C	T	0.11	0.33	0.09	0.28						Mtf1			1 hit	NEK10	
3	27198844	0.82	0.94	rs12487557	T	C	0.11	0.33	0.09	0.28						Hoxb3,NRSF,Zfx			1 hit	NEK10	
3	27200445	0.82	0.94	rs724243	C	A	0.11	0.33	0.09	0.28			GI			5 altered motifs			1 hit	NEK10	
3	27200662	0.81	0.94	rs724244	C	T	0.11	0.34	0.09	0.29			GI			10 altered motifs			1 hit	NEK10	
3	27200749	0.81	0.94	rs724245	G	T	0.11	0.34	0.09	0.29			GI, PANC			VDR			1 hit	NEK10	
3	27201554	0.83	0.95	rs17680166	G	C	0.11	0.33	0.09	0.29			GI, LNG	4 tissues		STAT			1 hit	NEK10	
3	27201628	0.83	0.95	rs17680194	G	A	0.12	0.33	0.09	0.29			GI, LNG	4 tissues		Pou2f2			1 hit	NEK10	
3	27201920	0.8	0.94	rs11923962	A	G	0.11	0.34	0.09	0.29			ESDR			4 altered motifs			1 hit	NEK10	
3	27202075	0.81	0.94	rs11916263	C	T	0.11	0.33	0.09	0.28			ESDR			6 altered motifs			1 hit	NEK10	
3	27202380	0.83	0.95	rs34940413	T	C	0.11	0.33	0.09	0.29			GI			4 altered motifs			1 hit	NEK10	
3	27202951	0.83	0.95	rs34446123	C	T	0.11	0.33	0.09	0.29			GI, PANC			Irf			1 hit	NEK10	
3	27203074	0.83	0.95	rs11712907	T	C	0.11	0.33	0.09	0.29			GI, PANC	ESDR		CTCF,Rad21,SMC3			1 hit	NEK10	
3	27203082	0.83	0.95	rs11708854	G	A	0.11	0.33	0.09	0.29			GI, PANC			PU.1,SPIB,TEF-1			1 hit	NEK10	
3	27203218	0.83	0.95	rs11708844	C	T	0.11	0.33	0.09	0.29			PANC	ESDR,LNG		Gfi1			1 hit	NEK10	
3	27203734	0.83	0.95	rs6792693	A	G	0.11	0.33	0.09	0.29						4 altered motifs		2 hits	1 hit	NEK10	
3	27203757	0.83	0.95	rs35729955	TA	T	0.11	0.33	0.09	0.29						6 altered motifs				NEK10	
3	27203902	0.82	0.94	rs11129268	G	A	0.10	0.34	0.09	0.28						6 altered motifs			1 hit	NEK10	
3	27203927	0.83	0.95	rs11709858	C	T	0.10	0.33	0.09	0.29						6 altered motifs			1 hit	NEK10	
3	27203995	0.83	0.95	rs36037509	T	G	0.11	0.33	0.10	0.29						Mef2,ZEB1			1 hit	NEK10	
3	27204109	0.83	0.95	rs36123482	C	T	0.11	0.33	0.09	0.29						Foxp3,Irf,Sox			1 hit	NEK10	
3	27206310	0.83	0.95	rs71323271	T	C	0.11	0.33	0.09	0.29									1 hit	NEK10	
3	27206531	0.83	0.95	rs34028315	T	C	0.11	0.33	0.09	0.29			IPSC			ELF1,Ets,ZNF263			1 hit	NEK10	
3	27206672	0.8	0.95	rs4973755	G	A	0.11	0.33	0.09	0.29			IPSC			CTCF,DEC,Gm397			1 hit	NEK10	
3	27206858	0.83	0.95	rs4973757	G	A	0.11	0.33	0.09	0.29			IPSC			RP58			1 hit	NEK10	
3	27207621	0.83	0.95	rs6793827	C	T	0.11	0.33	0.09	0.29						8 altered motifs			1 hit	NEK10	
3	27207793	0.83	0.95	rs6806213	A	G	0.11	0.33	0.09	0.29			BRN			4 altered motifs			1 hit	NEK10	
3	27207864	0.83	0.95	rs6806325	A	G	0.12	0.33	0.09	0.29			BRN			Hoxb3,RXRA			1 hit	NEK10	
3	27208377	0.83	0.95	rs13086192	T	A	0.11	0.33	0.09	0.29			BRN			7 altered motifs			1 hit	NEK10	
3	27208512	0.83	0.95	rs13063897	C	G	0.11	0.33	0.09	0.29			BRN, BLD			AP-1,TATA			1 hit	NEK10	
3	27208719	0.83	0.95	rs17629230	T	C	0.11	0.33	0.09	0.29			BRN, GI, BLD			STAT,p300			1 hit	NEK10	
3	27208774	0.83	0.95	rs35798440	G	T	0.11	0.33	0.09	0.29			BRN, GI, BLD	BRN		NF-kappaB			1 hit	NEK10	
3	27208792	0.83	0.95	rs17629266	T	C	0.11	0.33	0.09	0.29			BRN, GI, BLD	BRN,BRN					1 hit	NEK10	
3	27208881	0.81	0.94	rs35585874	C	T	0.11	0.33	0.09	0.28			BRN, GI, BLD	BRN		Nrf1				NEK10	
3	27208901	0.83	0.95	rs13079813	G	A	0.11	0.33	0.09	0.29			BRN, GI, BLD	BRN,BRN		HNF1			1 hit	NEK10	
3	27209111	0.82	0.95	rs35388578	A	AT	0.11	0.34	0.09	0.29			BRN, GI, BLD	BRN,BRN		5 altered motifs				NEK10	
3	27209367	0.83	0.95	rs11713802	T	C	0.11	0.33	0.09	0.29			4 tissues	BRN,BRN,LNG		Ets,Mef2		1 hit	1 hit	NEK10	
3	27209400	0.83	0.95	rs4973860	A	G	0.11	0.33	0.09	0.29			4 tissues	BRN,BRN,LNG		10 altered motifs		2 hits	1 hit	NEK10	
3	27209559	0.83	0.95	rs4973861	G	T	0.11	0.33	0.09	0.29			5 tissues	BRN,BRN,LNG		5 altered motifs			1 hit	NEK10	
3	27210035	0.83	0.95	rs35293137	TA	T	0.10	0.33	0.09	0.29			4 tissues			6 altered motifs				NEK10	
3	27210238	0.83	0.95	rs7613352	C	T	0.11	0.34	0.09	0.29			4 tissues			NRSF,Rad21			1 hit	NEK10	
3	27210527	0.82	0.95	rs2120893	G	A	0.11	0.33	0.09	0.29			BRN, BLD					1 hit	1 hit	NEK10	
3	27210699	0.83	0.95	rs7629551	T	C	0.12	0.33	0.09	0.29			BRN, BLD			AFP1,DMRT4,Nkx6-2			1 hit	NEK10	
3	27211212	0.83	0.95	rs11920408	C	T	0.11	0.33	0.09	0.29			BRN			TEF-1			1 hit	NEK10	
3	27211278	0.83	0.95	rs11928065	A	G	0.11	0.33	0.09	0.29			BRN			5 altered motifs			1 hit	NEK10	
3	27211773	0.83	0.95	rs35031465	A	G	0.12	0.33	0.09	0.29						STAT			1 hit	NEK10	
3	27212223	0.83	0.95	rs11129269	A	G	0.12	0.33	0.09	0.29						Pou2f2			1 hit	NEK10	
3	27212869	0.83	0.95	rs13061056	C	T	0.11	0.33	0.09	0.29			ESDR, ESC, IPSC	ESC		SP1,TATA			1 hit	NEK10	
3	27213548	0.83	0.95	rs62256304	C	T	0.11	0.33	0.09	0.29		IPSC	ESC, ESDR, IPSC	ESC,ESC,IPSC	BCL11A,NANOG,SP1	Nr2f2			1 hit	NEK10	
3	27213834	0.82	0.94	rs35692972	AT	A	0.11	0.33	0.09	0.28		IPSC	ESC, IPSC	ESC		Mef2,PLZF,TATA				NEK10	
3	27214301	0.83	0.95	rs11715168	G	T	0.11	0.33	0.09	0.29			ESC, IPSC						1 hit	NEK10	
3	27214373	0.83	0.95	rs11715136	C	T	0.11	0.33	0.09	0.29			ESC, IPSC			7 altered motifs			1 hit	NEK10	
3	27214613	0.83	0.95	rs35840526	A	G	0.11	0.33	0.10	0.29			ESC			BCL,HDAC2			1 hit	NEK10	
3	27215052	0.82	0.95	rs7642367	T	C	0.11	0.33	0.09	0.29			ESC			5 altered motifs			1 hit	NEK10	
3	27215511	0.83	0.95	rs35896441	A	G	0.11	0.33	0.09	0.29						GATA,PLZF			1 hit	NEK10	
3	27217242	0.83	0.95	rs13085421	A	G	0.11	0.33	0.09	0.29			11 tissues	8 tissues	4 bound proteins	6 altered motifs			1 hit	NEK10	intronic
3	27219848	0.83	0.96	rs1596701	T	C	0.12	0.33	0.09	0.29			5 tissues		CFOS	5 altered motifs			1 hit	NEK10	intronic
3	27222039	0.83	0.96	rs13096403	A	G	0.11	0.34	0.09	0.29						KAP1,PU.1,ZNF263		1 hit	1 hit	NEK10	intronic
3	27224060	0.83	0.95	rs6796032	C	A	0.12	0.34	0.09	0.29			6 tissues	BRST,SKIN					1 hit	NEK10	intronic
3	27226533	0.92	0.98	rs62256309	G	A	0.11	0.32	0.10	0.28						AP-2,EBF			2 hits	NEK10	intronic
3	27229431	0.93	0.99	rs2120891	A	G	0.11	0.36	0.11	0.28						TATA			2 hits	NEK10	intronic
3	27230516	0.93	0.99	rs62256312	T	C	0.12	0.36	0.11	0.28						SEF-1,TBX5			2 hits	NEK10	intronic
3	27230587	0.93	0.99	rs62256313	G	C	0.20	0.37	0.11	0.28						DMRT3,Foxo,RREB-1			2 hits	NEK10	intronic
3	27232073	0.93	0.99	rs2218472	T	C	0.22	0.37	0.11	0.28						NF-AT1		1 hit	1 hit	NEK10	intronic
3	27233966	0.93	0.99	rs150637381	T	A	0.22	0.37	0.11	0.28						TATA				NEK10	intronic
3	27235552	0.92	0.99	rs2197435	C	G	0.20	0.37	0.11	0.28						10 altered motifs			2 hits	NEK10	intronic
3	27235593	0.92	0.99	rs2197434	C	T	0.20	0.37	0.11	0.28						Pou1f1			2 hits	NEK10	intronic
3	27236723	0.9	0.99	rs2887898	G	T	0.08	0.35	0.07	0.28						6 altered motifs			1 hit	NEK10	intronic
3	27237932	0.91	0.99	rs4586769	G	A	0.15	0.36	0.11	0.28						CACD,Klf4,Pax-4			2 hits	NEK10	intronic
3	27238119	0.92	0.99	rs35993550	T	C	0.03	0.34	0.06	0.28						6 altered motifs				NEK10	intronic
3	27240198	0.93	0.99	rs7626869	T	A	0.20	0.37	0.11	0.28				BRN		HNF1,NF-AT1			2 hits	NEK10	intronic
3	27240278	0.93	0.99	rs1158719	C	A	0.12	0.36	0.11	0.28				BRN,BRN		Myb,Pbx-1,TCF4			3 hits	NEK10	intronic
3	27241626	0.93	0.99	rs11129271	A	T	0.20	0.37	0.11	0.28						HDAC2,Maf,STAT			2 hits	NEK10	intronic
3	27242093	0.92	0.99	rs62258161	A	G	0.12	0.36	0.11	0.28						Pou3f3,Sox			2 hits	NEK10	intronic
3	27242592	0.93	0.99	rs1373771	A	G	0.20	0.37	0.11	0.28						4 altered motifs			2 hits	NEK10	intronic
3	27243056	0.91	0.97	rs1373770	T	A	0.20	0.37	0.11	0.28						Arid3a,HMG-IY,Mef2			2 hits	NEK10	intronic
3	27243741	0.92	0.99	rs11925748	A	T	0.11	0.36	0.11	0.28						EWSR1-FLI1,STAT			2 hits	NEK10	intronic
3	27244175	0.92	0.99	rs1373769	C	T	0.20	0.37	0.11	0.28						Maf			2 hits	NEK10	intronic
3	27244237	0.92	0.99	rs1373768	G	A	0.20	0.37	0.11	0.28									2 hits	NEK10	intronic
3	27244747	0.92	0.99	rs77443176	G	T	0.11	0.36	0.11	0.28					CTCF	5 altered motifs			2 hits	NEK10	intronic
3	27246389	0.91	0.97	rs7643409	A	T	0.20	0.37	0.11	0.28						6 altered motifs			2 hits	NEK10	intronic
3	27247296	0.93	0.99	rs78366953	C	T	0.11	0.36	0.11	0.28						4 altered motifs			2 hits	NEK10	intronic
3	27249049	0.94	0.99	rs12494684	A	T	0.19	0.37	0.11	0.28						Myf,VDR			1 hit	NEK10	intronic
3	27249080	0.94	0.99	rs12488241	C	T	0.11	0.36	0.11	0.28						4 altered motifs			1 hit	NEK10	intronic
3	27249245	0.88	0.99	rs9826475	C	T	0.23	0.39	0.13	0.29									1 hit	NEK10	intronic
3	27249652	0.94	0.99	rs10865813	C	T	0.19	0.37	0.11	0.28						GR			1 hit	NEK10	intronic
3	27250312	0.86	0.94	rs11129273	A	G	0.18	0.37	0.11	0.26						BDP1,LUN-1			1 hit	NEK10	intronic
3	27250590	0.94	0.99	rs11129274	T	C	0.19	0.37	0.11	0.28						T3R,TATA			1 hit	NEK10	intronic
3	27250796	0.86	0.96	rs57030353	G	C	0.17	0.33	0.11	0.25									1 hit	NEK10	intronic
3	27250797	0.86	0.96	rs62258163	T	A	0.09	0.32	0.11	0.25						CHOP::CEBPalpha				NEK10	intronic
3	27253016	0.92	0.97	rs2166214	A	T	0.19	0.35	0.11	0.27						7 altered motifs			1 hit	NEK10	intronic
3	27253342	0.94	0.99	rs2120887	A	G	0.19	0.37	0.11	0.28						Ik-1,STAT			1 hit	NEK10	intronic
3	27255305	0.94	0.99	rs1550769	C	G	0.19	0.37	0.10	0.28									1 hit	NEK10	intronic
3	27255331	0.94	0.99	rs1550768	A	T	0.19	0.37	0.10	0.28						STAT		2 hits	1 hit	NEK10	intronic

3	27256003	0.94	0.99	rs12488113	A	C	0.19	0.37	0.10	0.28						6 altered motifs		1 hit	NEK10	intronic
3	27256128	0.94	0.99	rs12488147	A	T	0.19	0.37	0.10	0.28						7 altered motifs		1 hit	NEK10	intronic
3	27256193	0.94	0.99	rs12494667	C	A	0.19	0.37	0.10	0.28						5 altered motifs	1 hit	1 hit	NEK10	intronic
3	27256677	0.94	0.99	rs17681084	A	G	0.11	0.36	0.10	0.28						ERalpha-a,RORalpha1		1 hit	NEK10	intronic
3	27257193	0.94	0.99	rs11718155	C	T	0.19	0.37	0.10	0.28						E2A,TBX5,ZEB1		1 hit	NEK10	intronic
3	27257933	0.88	0.95	rs11129275	A	C	0.18	0.35	0.09	0.26						5 altered motifs		1 hit	NEK10	intronic
3	27258123	0.93	0.99	rs11129276	A	G	0.19	0.37	0.10	0.28						7 altered motifs		1 hit	NEK10	intronic
3	27258152	0.93	0.99	rs11129277	A	G	0.19	0.37	0.10	0.28						4 altered motifs		1 hit	NEK10	intronic
3	27258271	0.95	0.99	rs11719299	C	A	0.11	0.36	0.10	0.27						Foxp3		1 hit	NEK10	intronic
3	27258467	0.88	0.96	rs76985623	G	A	0.18	0.34	0.10	0.26						4 altered motifs			NEK10	intronic
3	27259443	0.89	0.98	rs149465972	C	T	0.21	0.35	0.09	0.25						AP-1,Mef2		1 hit	NEK10	intronic
3	27259572	0.95	0.99	rs140750999	G	T	0.12	0.36	0.10	0.27						5 altered motifs		1 hit	NEK10	intronic
3	27259832	0.96	0.99	rs151072467	T	A	0.11	0.35	0.10	0.27						Arid5a,Foxj2,HNF6		1 hit	NEK10	intronic
3	27260095	0.89	0.97	rs147864955	T	C	0.17	0.33	0.09	0.26						MIF-1,NF-Y			NEK10	intronic
3	27261905	0.95	1	rs6804742	C	T	0.18	0.37	0.10	0.28								1 hit	NEK10	intronic
3	27262218	1	1	rs4973758	T	C	0.17	0.35	0.10	0.27						CEBPB,NF-kappaB,STAT		1 hit	NEK10	intronic
3	27262463	0.93	0.98	rs6771664	A	G	0.17	0.34	0.09	0.26								1 hit	NEK10	intronic
3	27262565	0.93	0.99	rs4973864	G	A	0.10	0.33	0.09	0.26						6 altered motifs		1 hit	NEK10	intronic
3	27262704	0.95	1	rs4973759	T	C	0.18	0.36	0.10	0.28						CDP		1 hit	NEK10	intronic
3	27262833	0.83	0.99	rs138587830	G	A	0.17	0.35	0.09	0.24						TCF4		1 hit	NEK10	intronic
3	27262891	0.93	0.98	rs147444841	G	T	0.18	0.36	0.09	0.27						SP1,p300		1 hit	NEK10	intronic
3	27263060	0.94	0.99	rs150704795	G	A	0.11	0.35	0.10	0.27									NEK10	intronic
3	27263599	0.93	0.97	rs7624007	T	C	0.17	0.36	0.10	0.27								1 hit	NEK10	intronic
3	27264123	0.95	1	rs11129278	G	C	0.19	0.37	0.10	0.28							2 hits	1 hit	NEK10	intronic
3	27264736	0.95	1	rs6790036	G	A	0.19	0.37	0.10	0.28						6 altered motifs		1 hit	NEK10	intronic
3	27265043	0.95	1	rs35525805	A	G	0.11	0.36	0.10	0.28								1 hit	NEK10	intronic
3	27265505	0.95	1	rs6793238	G	C	0.19	0.37	0.10	0.28						Nanog,Pou2f2,TATA		1 hit	NEK10	intronic
3	27265737	0.91	0.96	rs141466233	GTTAT	G	0.11	0.35	0.10	0.27						11 altered motifs		1 hit	NEK10	intronic
3	27265951	0.92	0.98	rs6551190	T	C	0.19	0.37	0.10	0.28						E2A,Myf,ZEB1		1 hit	NEK10	intronic
3	27266159	0.95	1	rs7617029	C	T	0.20	0.37	0.10	0.28						Foxj2,Mef2,Nkx6-1		1 hit	NEK10	intronic
3	27266426	0.95	1	rs62255582	T	C	0.19	0.37	0.10	0.28						CTCF,Nkx1-1,STAT		1 hit	NEK10	intronic
3	27270067	0.94	0.99	rs12497765	G	A	0.11	0.36	0.10	0.28			GI					1 hit	NEK10	intronic
3	27271025	0.95	1	rs73161105	T	C	0.26	0.38	0.10	0.28								1 hit	NEK10	intronic
3	27271631	0.95	1	rs12486588	G	T	0.26	0.38	0.10	0.28						Foxp1		1 hit	NEK10	intronic
3	27271714	0.95	1	rs12492966	A	G	0.27	0.37	0.10	0.28						DMRT1,Spz1		1 hit	NEK10	intronic
3	27271778	0.95	1	rs12492994	A	G	0.26	0.38	0.10	0.28						5 altered motifs		1 hit	NEK10	intronic
3	27272384	0.95	1	rs11920191	T	G	0.26	0.38	0.10	0.28								1 hit	NEK10	intronic
3	27272995	0.94	0.99	rs7635995	T	G	0.11	0.36	0.10	0.28						5 altered motifs		1 hit	NEK10	intronic
3	27275010	0.94	0.99	rs4973866	G	T	0.11	0.36	0.10	0.28			GI			5 altered motifs		1 hit	NEK10	intronic
3	27275079	0.94	0.99	rs4973867	C	A	0.19	0.37	0.10	0.28			GI			AP-3,CEBPB,RXRA		1 hit	NEK10	intronic
3	27275428	0.94	0.99	rs4973868	C	A	0.26	0.38	0.10	0.28			GI, LIV	BRN,GI		Hoxa7,Maf	1 hit	1 hit	NEK10	intronic
3	27275760	0.94	0.99	rs62255585	A	G	0.26	0.38	0.10	0.28			GI, LIV	GI				1 hit	NEK10	intronic
3	27276167	0.94	0.99	rs11711845	G	A	0.26	0.38	0.10	0.28			FAT, GI, LIV			Pou3f1,Sox		1 hit	NEK10	intronic
3	27276858	0.94	0.99	rs62255586	G	T	0.11	0.36	0.10	0.28			FAT, GI					1 hit	NEK10	intronic
3	27276945	0.94	0.99	rs62255587	C	A	0.19	0.37	0.10	0.28			GI			ERalpha-a		1 hit	NEK10	intronic
3	27277245	0.94	0.99	rs140759829	A	11-mer	0.27	0.38	0.10	0.28			GI			Roaz,SREBP,VDR			NEK10	intronic
3	27278524	0.85	0.99	rs73161139	T	G	0.36	0.41	0.15	0.30			6 tissues	5 tissues	USF1	4 altered motifs		1 hit	NEK10	intronic
3	27278864	0.94	0.99	rs62255589	A	G	0.26	0.38	0.10	0.28		LIV	6 tissues			8 altered motifs		1 hit	NEK10	intronic
3	27280125	0.94	0.99	rs62255591	G	A	0.12	0.36	0.10	0.28			ESDR			5 altered motifs		1 hit	NEK10	intronic
3	27280311	0.94	0.99	rs5847455	TA	T	0.26	0.38	0.10	0.28			ESDR, BRN			8 altered motifs		1 hit	NEK10	intronic
3	27280574	0.94	0.99	rs1542152	A	T	0.19	0.37	0.10	0.28			LIV			6 altered motifs		1 hit	NEK10	intronic
3	27280716	0.94	0.99	rs11919927	G	A	0.26	0.38	0.10	0.28						GR,HDAC2,Pax-5		1 hit	NEK10	intronic
3	27280852	0.94	0.99	rs11129279	A	C	0.26	0.38	0.10	0.28						DMRT5,Foxq1,Osr		1 hit	NEK10	intronic
3	27281891	0.94	0.99	rs6788621	C	T	0.26	0.38	0.10	0.28		LIV	GI	GI,LIV	FOXA1,TCF4	Cdx		1 hit	NEK10	intronic
3	27284113	0.82	0.93	rs6762125	T	C	0.19	0.37	0.10	0.28						6 altered motifs		1 hit	NEK10	intronic
3	27284606	0.82	0.93	rs11129280	G	T	0.13	0.36	0.10	0.28						4 altered motifs		1 hit	NEK10	synonymous
3	27284960	0.82	0.94	rs10510594	A	G	0.11	0.37	0.10	0.28						Foxj2,TATA		1 hit	NEK10	synonymous
3	27285062	0.82	0.93	rs34894168	AC	A	0.11	0.36	0.12	0.28						Ik-2,Znf143		1 hit	NEK10	intronic
3	27285904	0.81	0.93	rs79842554	T	C	0.13	0.37	0.10	0.28					CEBPB	7 altered motifs		1 hit	NEK10	intronic
3	27286660	0.82	0.93	rs112557637	G	C	0.11	0.36	0.10	0.28						COMP1		1 hit	NEK10	intronic
3	27287926	0.82	0.93	rs10510593	A	C	0.11	0.36	0.10	0.28								1 hit	NEK10	intronic
3	27291329	0.82	0.93	rs10510592	A	G	0.13	0.36	0.10	0.28						TCF4		1 hit	NEK10	missense
3	27291963	0.82	0.93	rs4504137	A	C	0.11	0.36	0.10	0.28						NF-E2		1 hit	NEK10	intronic
3	27296438	0.82	0.93	rs4973869	G	A	0.13	0.36	0.10	0.28						GR	1 hit	1 hit	NEK10	intronic
3	27296933	0.82	0.93	rs4973870	C	A	0.08	0.35	0.10	0.28						Foxp1	2 hits	1 hit	NEK10	intronic
3	27297646	0.82	0.93	rs62255638	A	G	0.11	0.36	0.10	0.28						Cdx2,Hltf		1 hit	NEK10	intronic
3	27298092	0.82	0.93	rs1844984	C	T	0.09	0.36	0.10	0.28						TBX5			NEK10	intronic
3	27298632	0.82	0.93	rs4634071	C	A	0.08	0.35	0.10	0.28						5 altered motifs		1 hit	NEK10	intronic
3	27309795	0.8	0.91	rs1445112	A	G	0.19	0.37	0.09	0.27						6 altered motifs		1 hit	NEK10	intronic
3	27314050	0.8	0.91	rs1472145	T	C	0.11	0.36	0.09	0.27						8 altered motifs		1 hit	NEK10	intronic
3	27316230	0.8	0.91	rs17239342	G	C	0.11	0.36	0.09	0.27			GI			RXRA		1 hit	NEK10	intronic
3	27316521	0.8	0.91	rs12152280	A	T	0.11	0.36	0.09	0.27			GI, LIV			Pou6f1		1 hit	NEK10	intronic

Query SNP: rs6465348 and variants with r^2 >= 0.8

chr	pos (hg38)	LD (r^2)	LD (D')	variant	Ref	Alt	AFR freq	AMR freq	ASN freq	EUR freq	SiPhy cons	Promoter histone marks	Enhancer histone marks	DNAse	Proteins bound	Motifs changed	NHGRI/EBI GWAS hits	GRASP QTL hits	Selected eQTL hits	GENCODE genes	dbSNP func annot
7	91917013	0.8	-0.91	rs405	G	C	0.73	0.63	0.83	0.60						TCF12			6 hits	24kb 5' of AKAP9	
7	91917212	0.8	-0.91	rs406	C	A	0.67	0.62	0.83	0.60									7 hits	24kb 5' of AKAP9	
7	91918055	0.8	-0.91	rs2023785	C	A	0.67	0.62	0.83	0.60						Mef2			7 hits	23kb 5' of AKAP9	
7	91920343	0.8	-0.92	rs2013334	G	A	0.61	0.62	0.83	0.60						HNF1			7 hits	21kb 5' of AKAP9	
7	91920623	0.8	-0.92	rs11769294	A	G	0.60	0.62	0.83	0.60						Sox			9 hits	20kb 5' of AKAP9	
7	91922474	0.8	-0.92	rs13237856	G	A	0.58	0.62	0.83	0.60						7 altered motifs			7 hits	18kb 5' of AKAP9	
7	91928111	0.85	-0.92	rs410	C	G	0.55	0.62	0.83	0.61			LIV		CTCF,RAD21				9 hits	13kb 5' of AKAP9	
7	91928444	0.85	-0.93	rs411	C	G	0.55	0.62	0.83	0.61			LIV			6 altered motifs			10 hits	12kb 5' of AKAP9	
7	91929012	0.84	-0.92	rs11772102	A	T	0.51	0.62	0.83	0.61						Msx2			10 hits	12kb 5' of AKAP9	
7	91929209	0.84	-0.92	rs10246036	G	A	0.55	0.62	0.83	0.61						Hoxa9			9 hits	12kb 5' of AKAP9	
7	91930167	0.85	-0.93	rs12671991	T	C	0.55	0.62	0.83	0.61									10 hits	11kb 5' of AKAP9	
7	91932278	0.85	-0.93	rs4729015	T	G	0.56	0.62	0.83	0.61						13 altered motifs		5 hits	10 hits	8.6kb 5' of AKAP9	
7	91938178	0.83	-0.92	rs9969198	G	T	0.51	0.62	0.83	0.61						4 altered motifs			10 hits	2.7kb 5' of AKAP9	
7	91938958	0.83	-0.92	rs1859037	A	G	0.56	0.62	0.83	0.61								5 hits	9 hits	1.9kb 5' of AKAP9	
7	91940815	0.83	-0.92	rs4727266	C	T	0.48	0.62	0.83	0.61		24 tissues		53 tissues	28 bound proteins	4 altered motifs			10 hits	51bp 5' of AKAP9	
7	91940976	0.83	-0.92	rs4727267	G	C	0.49	0.62	0.83	0.61		24 tissues		53 tissues	19 bound proteins	11 altered motifs			10 hits	AKAP9	5'-UTR
7	91941301	0.82	-0.91	rs7797369	C	A	0.49	0.62	0.83	0.61		24 tissues		33 tissues	AP2ALPHA,ZBTB7A	8 altered motifs			10 hits	AKAP9	intronic
7	91941476	0.83	-0.92	rs6465339	T	A	0.32	0.61	0.81	0.61		24 tissues		34 tissues	POL2	4 altered motifs			11 hits	AKAP9	intronic
7	91943994	0.83	-0.92	rs2961020	G	A	0.71	0.63	0.82	0.61		BLD	BLD			4 altered motifs			8 hits	AKAP9	intronic
7	91944796	0.83	-0.92	rs12666179	T	C	0.32	0.61	0.81	0.61			BLD			Hmbox1			11 hits	AKAP9	intronic
7	91947510	0.81	-0.91	rs10259526	G	T	0.82	0.64	0.81	0.60						4 altered motifs			5 hits	AKAP9	intronic
7	91947644	0.84	-0.92	rs10953063	A	G	0.52	0.62	0.83	0.61						4 altered motifs			11 hits	AKAP9	intronic
7	91947931	0.84	-0.92	rs28412174	T	G	0.49	0.62	0.83	0.61				BLD		Mef2,NRSF			10 hits	AKAP9	intronic
7	91949133	0.84	-0.92	rs34043009	T	C	0.49	0.62	0.83	0.61			BLD, GI			20 altered motifs			10 hits	AKAP9	intronic
7	91949325	0.84	-0.92	rs10264666	G	A	0.72	0.63	0.83	0.61				8 tissues	4 bound proteins	Pbx3,TCF4			8 hits	AKAP9	intronic
7	91950402	0.83	-0.91	rs4236524	C	T	0.53	0.62	0.83	0.61			7 tissues			Mef2,Pax-4,SIX5			9 hits	AKAP9	intronic
7	91950973	0.84	-0.92	rs2282968	A	C	0.71	0.63	0.83	0.61				OVRY		9 altered motifs			8 hits	AKAP9	intronic
7	91951176	0.84	-0.92	rs415	A	G	0.53	0.62	0.82	0.61						6 altered motifs		1 hit	10 hits	AKAP9	intronic
7	91951257	0.81	-0.91	rs416	A	G	0.48	0.62	0.83	0.61						HNF4,Irf			10 hits	AKAP9	intronic
7	91951807	0.83	-0.91	rs417	C	A	0.32	0.60	0.80	0.61			BLD						11 hits	AKAP9	intronic
7	91952252	0.84	-0.92	rs17164263	T	A	0.52	0.62	0.83	0.61			BLD, SKIN, PANC	BLD		Rad21			11 hits	AKAP9	intronic
7	91952869	0.86	-0.93	rs10280620	C	G	0.52	0.62	0.82	0.61						4 altered motifs			11 hits	AKAP9	intronic
7	91952891	0.84	-0.92	rs10280927	G	A	0.52	0.62	0.83	0.61						HNF4,RXR::LXR			11 hits	AKAP9	intronic
7	91953818	0.82	-0.91	rs56898654	CA	C	0.52	0.62	0.82	0.61				6 tissues	CTCF,RAD21	22 altered motifs			8 hits	AKAP9	intronic
7	91954458	0.84	-0.92	rs2282969	T	C	0.51	0.63	0.83	0.61			BLD			TATA,Zfp105			10 hits	AKAP9	intronic
7	91954967	0.85	0.93	rs2282970	A	G	0.68	0.39	0.19	0.39				BLD		4 altered motifs			10 hits	AKAP9	intronic
7	91955042	0.85	0.93	rs2282971	A	G	0.65	0.39	0.19	0.39				BLD		p300			10 hits	AKAP9	intronic
7	91955944	0.85	0.93	rs58965837	T	A	0.52	0.38	0.17	0.39			BLD, PANC			4 altered motifs			10 hits	AKAP9	intronic
7	91957075	0.85	0.93	rs756647	A	G	0.51	0.38	0.17	0.39						Pou2f2,Pou5f1		1 hit	11 hits	AKAP9	intronic
7	91957736	0.85	0.93	rs10235941	T	G	0.52	0.38	0.17	0.39					KAP1	CEBPB,Pou2f2,RXRA			10 hits	AKAP9	intronic
7	91958163	0.85	0.93	rs10279943	G	C	0.48	0.38	0.17	0.39				ADRL		Pou5f1			9 hits	AKAP9	intronic
7	91958722	0.85	0.93	rs12530646	G	A	0.47	0.38	0.17	0.39			ADRL			8 altered motifs		1 hit	10 hits	AKAP9	intronic
7	91958880	0.8	0.92	rs7781597	T	C	0.48	0.37	0.20	0.37			ADRL			9 altered motifs			6 hits	AKAP9	intronic
7	91959046	0.85	0.93	rs7798973	G	A	0.49	0.38	0.17	0.39			5 tissues			6 altered motifs			10 hits	AKAP9	intronic
7	91959350	0.84	0.92	rs6465340	G	A	0.51	0.38	0.17	0.39			4 tissues		CEBPB	4 altered motifs			10 hits	AKAP9	intronic
7	91961094	0.85	0.93	rs17164275	A	G	0.48	0.37	0.17	0.39						4 altered motifs			11 hits	AKAP9	intronic
7	91961332	0.83	0.92	rs58413301	G	C	0.51	0.38	0.17	0.39						CACD,Klf4,LBP-1			10 hits	AKAP9	intronic
7	91962836	0.85	0.93	rs68124929	CTG	C	0.48	0.38	0.17	0.39			VAS			11 altered motifs			10 hits	AKAP9	intronic
7	91963441	0.85	0.93	rs28735847	T	C	0.49	0.38	0.17	0.39						5 altered motifs			10 hits	AKAP9	intronic
7	91964914	0.84	0.93	rs6980247	T	A	0.68	0.39	0.19	0.39						19 altered motifs			11 hits	AKAP9	intronic
7	91965196	0.85	0.93	rs6942649	T	C	0.52	0.38	0.17	0.39						EWSR1-FLI1,Pax-4			12 hits	AKAP9	intronic
7	91965803	0.85	0.93	rs2188153	G	T	0.52	0.38	0.17	0.39				BLD		4 altered motifs			10 hits	AKAP9	intronic
7	91965824	0.86	0.93	rs2188154	T	A	0.49	0.38	0.17	0.39				BLD		Nkx3			10 hits	AKAP9	intronic
7	91966807	0.85	0.93	rs6465341	G	C	0.47	0.38	0.17	0.39			BLD			NR4A,ZEB1			9 hits	AKAP9	intronic
7	91968620	0.87	0.94	rs6952671	C	A	0.49	0.38	0.17	0.39			HRT			18 altered motifs			10 hits	AKAP9	intronic
7	91969129	0.87	0.94	rs10429103	T	A	0.52	0.38	0.18	0.39						6 altered motifs			10 hits	AKAP9	intronic
7	91970511	0.87	0.94	rs2188155	A	C,G	0.52	0.38	0.17	0.39			7 tissues	ESC,IPSC					12 hits	AKAP9	intronic
7	91971334	0.88	0.94	rs28615927	C	A	0.51	0.37	0.17	0.39						4 altered motifs			11 hits	AKAP9	intronic
7	91971964	0.87	0.93	rs35071949	CTT	C	0.48	0.38	0.17	0.39						6 altered motifs			3 hits	AKAP9	intronic
7	91971967	0.83	0.94	rs200209451	TTA	T	0.46	0.36	0.17	0.38						10 altered motifs			8 hits	AKAP9	intronic
7	91972151	0.88	0.94	rs13224513	G	A	0.49	0.38	0.17	0.39			7 tissues	ESC		HES1			10 hits	AKAP9	intronic
7	91972616	0.87	0.94	rs7806157	A	G	0.36	0.37	0.17	0.39			STRM, GI			Hoxd8,Pou6f1			10 hits	AKAP9	intronic
7	91972664	0.88	0.94	rs7806184	A	G	0.49	0.38	0.17	0.39			STRM, GI			Maf,RFX5			10 hits	AKAP9	intronic
7	91974913	0.88	0.94	rs10226078	T	C	0.52	0.38	0.17	0.39						GR,HNF4,Zbtb3			10 hits	AKAP9	intronic
7	91975911	0.81	0.94	rs201015377	TTG	T	0.62	0.38	0.19	0.37						18 altered motifs			8 hits	AKAP9	intronic
7	91978223	0.83	0.94	rs10605460	CAG	C	0.43	0.36	0.17	0.37						DMRT5,Zfp105			2 hits	AKAP9	intronic
7	91980797	0.87	0.94	rs10228800	T	G	0.51	0.38	0.17	0.39						Nkx3			12 hits	AKAP9	intronic
7	91981648	0.84	0.94	rs6976122	T	C	0.50	0.37	0.17	0.38						Smad			10 hits	AKAP9	intronic
7	91981840	0.87	0.94	rs6956309	A	G	0.67	0.39	0.20	0.39						5 altered motifs			10 hits	AKAP9	intronic
7	91983765	0.87	0.94	rs10225701	C	G	0.67	0.38	0.19	0.39				BLD					9 hits	AKAP9	intronic
7	91984139	0.87	0.94	rs74770876	C	T	0.48	0.37	0.17	0.39						16 altered motifs			10 hits	AKAP9	intronic
7	91984678	0.85	0.94	rs75497198	A	G	0.50	0.36	0.17	0.38						Egr-1,Hbp1,Nkx6-1			9 hits	AKAP9	intronic
7	91985514	0.87	0.94	rs34603510	G	A	0.48	0.38	0.17	0.39						Foxa,HNF1			10 hits	AKAP9	intronic
7	91986113	0.87	0.94	rs13230588	C	A	0.51	0.38	0.17	0.39			HRT						10 hits	AKAP9	intronic
7	91987577	0.87	0.94	rs10279210	G	A	0.68	0.39	0.19	0.39						Foxa,Foxd1,HDAC2			10 hits	AKAP9	intronic
7	91988293	0.81	0.94	rs1122308	C	A	0.49	0.35	0.17	0.37			LIV			RREB-1			13 hits	AKAP9	intronic
7	91988894	0.87	0.94	rs6465343	T	C	0.68	0.39	0.20	0.39			LIV			Pbx-1,THAP1,YY1			10 hits	AKAP9	intronic
7	91989139	0.83	0.95	rs200128597	TTAA	T	0.45	0.37	0.18	0.37						Hlx1,Sox			10 hits	AKAP9	intronic
7	91989140	0.87	0.94	rs146017465	TAATA	T	0.48	0.37	0.18	0.39						4 altered motifs			3 hits	AKAP9	intronic
7	91991557	0.87	0.94	rs7777123	C	T	0.48	0.38	0.17	0.39						6 altered motifs			9 hits	AKAP9	intronic
7	91993362	0.87	0.94	rs2237568	G	A	0.49	0.38	0.17	0.39			BLD			Mef2,TATA			10 hits	AKAP9	intronic
7	91997385	0.88	0.94	rs2299232	T	G	0.29	0.37	0.17	0.39						CTCF,Irf		2 hits	10 hits	AKAP9	intronic
7	91998186	0.88	0.94	rs2299235	C	T	0.49	0.38	0.18	0.39			ESDR, HRT						10 hits	AKAP9	intronic
7	91999279	0.88	0.94	rs2018628	G	T	0.49	0.38	0.17	0.39						AhR,RXRA,TCF4			10 hits	AKAP9	intronic
7	91999837	0.88	0.94	rs3753107	A	G	0.68	0.39	0.19	0.39			HRT			PTF1-beta,STAT,p300			12 hits	AKAP9	intronic
7	92000670	0.88	0.94	rs3753108	T	G	0.68	0.39	0.19	0.39			HRT						10 hits	AKAP9	intronic
7	92000687	0.88	0.94	rs3753109	G	C	0.52	0.38	0.17	0.39						6 altered motifs			10 hits	AKAP9	intronic
7	92001306	0.88	0.94	rs6964587	G	T	0.52	0.38	0.17	0.39						15 altered motifs		1 hit	12 hits	AKAP9	missense
7	92003899	0.88	0.94	rs12540565	T	G	0.50	0.38	0.17	0.39						Pax-1			13 hits	AKAP9	intronic
7	92005649	0.88	0.94	rs12539231	G	A	0.48	0.38	0.17	0.39						5 altered motifs			12 hits	AKAP9	intronic
7	92007696	0.88	0.94	rs35317449	G	A	0.53	0.37	0.17	0.39						FXR,LXR,Pou3f1			10 hits	AKAP9	intronic
7	92008862	0.88	0.94	rs28766948	T	A	0.49	0.37	0.17	0.39						E2A,Lmo2-complex,ZEB1			11 hits	AKAP9	intronic
7	92009137	0.88	0.94	rs28399886	A	G	0.48	0.38	0.17	0.39						TLX1::NFIC,YY1			10 hits	AKAP9	intronic
7	92009999	0.88	0.94	rs7455444	C	T	0.49	0.38	0.17	0.39						Ik-1			11 hits	AKAP9	intronic
7	92010959	0.88	0.94	rs6465344	G	A	0.68	0.39	0.19	0.39						9 altered motifs			12 hits	AKAP9	intronic
7	92011459	0.88	0.94	rs7785095	A	T	0.53	0.38	0.17	0.39						Gfi1,Gsc,Otx2			12 hits	AKAP9	intronic
7	92012614	0.88	0.94	rs13245393	A	G	0.53	0.38	0.17	0.39			SKIN					1 hit	12 hits	AKAP9	synonymous
7	92013400	0.88	0.94	rs6944591	T	C	0.52	0.38	0.17	0.39			SKIN			LBP-1,Spz1			10 hits	AKAP9	intronic
7	92013889	0.88	0.94	rs202142712	AC	A	0.50	0.38	0.18	0.39			SKIN	SKIN		14 altered motifs			9 hits	AKAP9	intronic
7	92013905	0.88	0.94	rs6967256	G	A	0.69	0.39	0.19	0.39			SKIN	SKIN		5 altered motifs			10 hits	AKAP9	intronic
7	92014756	0.88	0.94	rs28594877	C	T	0.69	0.39	0.19	0.39						HDAC2			10 hits	AKAP9	intronic
7	92015027	0.88	0.94	rs34863529	C	G	0.25	0.36	0.17	0.39						Eomes,Mef2,Pdx1			9 hits	AKAP9	intronic
7	92015239	0.88	0.94	rs7805077	A	G	0.52	0.38	0.17	0.39						4 altered motifs		1 hit	12 hits	AKAP9	intronic
7	92015838	0.88	0.94	rs10234071	G	C	0.49	0.38	0.17	0.39						PPAR,PU.1			13 hits	AKAP9	intronic
7	92015951	0.88	0.94	rs10263309	A	G	0.49	0.38	0.17	0.39						Pax-8			11 hits	AKAP9	intronic
7	92016884	0.88	0.94	rs7788092	C	T	0.68	0.39	0.20	0.39						8 altered motifs			10 hits	AKAP9	intronic
7	92018076	0.88	0.94	rs28410528	A	G	0.49	0.38	0.17	0.39			STRM, SKIN, OVRY			Irf,Sin3Ak-20			11 hits	AKAP9	intronic
7	92019027	0.88	0.94	rs2888851	G	A	0.49	0.38	0.17	0.39			SKIN	SKIN		4 altered motifs			11 hits	AKAP9	intronic
7	92019430	0.88	0.94	rs13221998	A	G	0.53	0.38	0.17	0.39						AP-1,CACD,RREB-1			10 hits	AKAP9	intronic
7	92019625	0.88	0.94	rs7802668	A	G	0.49	0.38	0.17	0.39						6 altered motifs			11 hits	AKAP9	intronic
7	92019687	0.86	0.94	rs7786031	T	G	0.52	0.38	0.19	0.38						8 altered motifs			11 hits	AKAP9	intronic
7	92024537	0.88	0.94	rs13231238	T	G	0.49	0.38	0.17	0.39			STRM, SKIN						10 hits	AKAP9	intronic
7	92025203	0.88	0.94	rs2299238	G	A	0.49	0.37	0.17	0.39		LNG, BRN	8 tissues	SKIN		Bbx,Maf		1 hit	12 hits	AKAP9	intronic
7	92025303	0.8	0.94	rs2299239	C	T	0.47	0.35	0.17	0.37		STRM, BRN	8 tissues	9 tissues					11 hits	AKAP9	intronic
7	92025304	0.8	0.94	rs2299240	A	G	0.66	0.37	0.19	0.37		STRM, BRN	8 tissues	9 tissues					10 hits	AKAP9	intronic
7	92027269	0.85	0.95	rs149208852	T	C	0.53	0.37	0.18	0.38						4 altered motifs			9 hits	AKAP9	intronic
7	92027802	0.88	0.94	rs147131837	A	G	0.53	0.38	0.17	0.39						BCL,NF-Y,NRSF			3 hits	AKAP9	intronic
7	92028680	0.88	0.94	rs6952389	A	G	0.54	0.38	0.17	0.39				MUS		PLZF,Sox			12 hits	AKAP9	intronic
7	92029306	0.88	0.94	rs6953515	C	T	0.29	0.37	0.17	0.39						Pax-4			10 hits	AKAP9	intronic
7	92029836	0.88	0.94	rs17164315	C	G	0.53	0.38	0.17	0.39						PLZF,Pou2f2			10 hits	AKAP9	intronic
7	92030739	0.88	0.94	rs10281556	A	G	0.49	0.38	0.17	0.39						4 altered motifs			13 hits	AKAP9	intronic
7	92030911	0.88	0.94	rs10488510	G	T	0.68	0.39	0.19	0.39						5 altered motifs			12 hits	AKAP9	intronic
7	92031797	0.85	0.94	rs6968870	G	C	0.53	0.37	0.17	0.38						Sox,TEF-1			10 hits	AKAP9	intronic
7	92033952	0.88	0.94	rs13231578	C	T	0.53	0.38	0.17	0.39			FAT	HRT		TEF-1			12 hits	AKAP9	intronic
7	92034050	0.88	0.94	rs7811564	A	G	0.49	0.38	0.17	0.39			FAT			Foxp3,Hoxb13,TATA			13 hits	AKAP9	intronic
7	92034215	0.88	0.94	rs13231820	A	G	0.25	0.36	0.17	0.39			FAT	OVRY		5 altered motifs			11 hits	AKAP9	intronic
7	92035331	0.88	0.95	rs7781221	A	G	0.49	0.38	0.17	0.39			ESC, IPSC, LIV			4 altered motifs			13 hits	AKAP9	intronic
7	92036313	0.85	0.94	rs57969083	A	T	0.67	0.38	0.19	0.38			ESDR			19 altered motifs			8 hits	AKAP9	intronic

7	92037055	0.89	0.95	rs2282972	C	T	0.49	0.38	0.17	0.39		LIV			Foxa,HNF1,Pou2f2		13 hits	AKAP9	intronic
7	92038012	0.89	0.94	rs12333563	G	A	0.52	0.38	0.17	0.39					Mrg1::Hoxa9,Myc,TATA		10 hits	AKAP9	intronic
7	92038378	0.89	0.95	rs2285333	T	G	0.49	0.38	0.17	0.39					6 altered motifs		13 hits	AKAP9	intronic
7	92039051	0.89	0.95	rs34548928	G	A	0.50	0.38	0.18	0.39					5 altered motifs		11 hits	AKAP9	intronic
7	92039540	0.89	0.95	rs7789492	C	T	0.68	0.39	0.19	0.39							10 hits	AKAP9	intronic
7	92040494	0.89	0.95	rs6961928	G	A	0.69	0.39	0.19	0.39			OVRY		11 altered motifs		10 hits	AKAP9	intronic
7	92041684	0.89	0.95	rs55745934	T	C	0.29	0.37	0.17	0.39		5 tissues					8 hits	AKAP9	intronic
7	92041693	0.89	0.96	rs10225885	A	G	0.48	0.37	0.17	0.39					Pax-6,Pou3f2	1 hit	12 hits	AKAP9	intronic
7	92041706	0.89	0.96	rs10225892	A	G	0.69	0.39	0.19	0.39					8 altered motifs		10 hits	AKAP9	intronic
7	92042539	0.89	0.95	rs926192	C	A	0.69	0.39	0.19	0.39	4 tissues	HRT, MUS, LIV	5 tissues		5 altered motifs	1 hit	12 hits	AKAP9	intronic
7	92043089	0.89	0.94	rs9784993	G	A	0.53	0.38	0.17	0.39	LIV, GI	PANC, MUS, GI			5 altered motifs		10 hits	AKAP9	intronic
7	92043994	0.89	0.94	rs10234434	A	T	0.49	0.38	0.17	0.39			6 tissues		5 altered motifs		12 hits	AKAP9	intronic
7	92044309	0.89	0.94	rs6979085	C	T	0.53	0.38	0.18	0.39	LIV, GI	5 tissues	8 tissues		Maf,NF-E2		10 hits	AKAP9	intronic
7	92044988	0.89	0.94	rs9785013	G	A	0.70	0.39	0.19	0.39	LIV	HRT, GI, MUS	GI,BLD		4 altered motifs		10 hits	AKAP9	intronic
7	92045493	0.88	0.94	rs11971885	T	C	0.50	0.37	0.17	0.39		LIV, HRT, GI	GI,OVRY		7 altered motifs		13 hits	AKAP9	intronic
7	92049984	0.89	0.94	rs36120033	G	A	0.53	0.38	0.17	0.39							10 hits	AKAP9	intronic
7	92050223	0.89	0.94	rs113152942	G	C	0.68	0.40	0.19	0.39					BCL,NRSF,Sin3Ak-20		10 hits	AKAP9	intronic
7	92050573	0.89	0.94	rs1859113	T	C	0.53	0.38	0.17	0.39					Zfp105		12 hits	AKAP9	intronic
7	92051327	0.89	0.94	rs6950470	G	A	0.69	0.39	0.19	0.39		HRT, GI			4 altered motifs		10 hits	AKAP9	intronic
7	92051341	0.88	0.94	rs6970277	T	C	0.25	0.36	0.17	0.39		HRT, GI			Esr2		9 hits	AKAP9	intronic
7	92052283	0.87	0.94	rs11365234	CA	C	0.69	0.39	0.20	0.39		4 tissues			E2F,Nanog		11 hits	AKAP9	intronic
7	92052285	0.83	0.95	rs199808757	AT	A	0.65	0.38	0.18	0.37		4 tissues			Sox		8 hits	AKAP9	intronic
7	92053534	0.9	0.96	rs1859114	T	C	0.50	0.38	0.17	0.39		4 tissues	GI		8 altered motifs	1 hit	13 hits	AKAP9	intronic
7	92054535	0.9	0.96	rs12531366	G	C	0.53	0.38	0.17	0.39		ESDR, GI					12 hits	AKAP9	intronic
7	92054772	0.9	0.96	rs12536529	A	G	0.50	0.38	0.17	0.39		ESDR, GI					13 hits	AKAP9	intronic
7	92056071	0.89	0.95	rs60455472	8-mer	A	0.53	0.38	0.17	0.39		LIV, GI	GI		16 altered motifs		10 hits	AKAP9	intronic
7	92057475	0.9	0.96	rs7791138	A	G	0.69	0.39	0.19	0.39		13 tissues	16 tissues	5 bound proteins	AIRE,Pou2f2		12 hits	AKAP9	intronic
7	92058037	0.9	0.96	rs1544377	A	G	0.50	0.38	0.17	0.39	GI, MUS	12 tissues	GI		10 altered motifs		11 hits	AKAP9	intronic
7	92059713	0.89	0.95	rs3041246	C	CAT	0.53	0.38	0.18	0.39	GI, MUS				5 altered motifs		9 hits	AKAP9	intronic
7	92060037	0.9	0.96	rs6969536	C	G	0.50	0.38	0.17	0.39	GI, MUS				4 altered motifs		13 hits	AKAP9	intronic
7	92060132	0.9	0.96	rs6951972	T	G	0.50	0.38	0.17	0.39	GI, MUS				Egr-1,Msx-1		11 hits	AKAP9	intronic
7	92060147	0.9	0.96	rs6969981	G	C	0.50	0.38	0.17	0.39	GI, MUS				4 altered motifs		11 hits	AKAP9	intronic
7	92061143	0.9	0.96	rs56072181	A	G	0.50	0.38	0.17	0.39	GI, MUS	7 tissues	SKIN,MUS		4 altered motifs		11 hits	AKAP9	intronic
7	92062287	0.9	0.96	rs10236397	C	T	0.49	0.38	0.17	0.39					6 altered motifs		11 hits	AKAP9	synonymous
7	92062632	0.9	0.96	rs7781066	G	T	0.53	0.38	0.17	0.39							10 hits	AKAP9	intronic
7	92063083	0.85	0.95	rs200486833	C	CT	0.51	0.36	0.17	0.38					9 altered motifs		10 hits	AKAP9	intronic
7	92063348	0.89	0.95	rs11981461	A	G	0.50	0.37	0.17	0.39		LIV			Myf		11 hits	AKAP9	intronic
7	92064523	0.9	0.96	rs6465347	A	G	0.49	0.38	0.17	0.39		LIV, GI, LNG	GI,GI		6 altered motifs	6 hits	13 hits	AKAP9	intronic
7	92067457	0.86	0.94	rs71107850	C	CC,CCT	0.52	0.37	0.16	0.38		GI					7 hits	AKAP9	intronic
7	92070465	0.9	0.96	rs10248102	G	A	0.49	0.38	0.17	0.39					DMRT2,Irf,PRDM1		11 hits	AKAP9	intronic
7	92070593	0.9	0.96	rs10280310	A	C	0.68	0.39	0.19	0.39					6 altered motifs		10 hits	AKAP9	intronic
7	92070666	0.9	0.96	rs34341778	C	T	0.49	0.38	0.17	0.39		GI, ADRL			ATF3,SREBP		11 hits	AKAP9	intronic
7	92071513	0.9	0.96	rs10270598	T	C	0.49	0.37	0.17	0.39					13 altered motifs		11 hits	AKAP9	intronic
7	92072032	0.9	0.96	rs7800582	G	A	0.49	0.37	0.17	0.39		GI			Foxa,Foxd3	1 hit	13 hits	AKAP9	intronic
7	92073426	0.9	0.96	rs11972410	A	C	0.50	0.38	0.17	0.39					BCL,Mtf1,RXRA		11 hits	AKAP9	intronic
7	92074258	0.89	0.94	rs11980125	C	T	0.53	0.38	0.17	0.39					IRC900814		9 hits	AKAP9	intronic
7	92074396	0.88	0.95	rs11973395	A	G	0.50	0.35	0.17	0.38							7 hits	AKAP9	intronic
7	92075960	0.91	0.96	rs11982047	G	A	0.25	0.36	0.17	0.39		BRST, SKIN, BLD	7 tissues	BATF	Mef2,RFX5,SREBP		9 hits	AKAP9	intronic
7	92076382	0.91	0.96	rs10243241	A	G	0.50	0.37	0.17	0.39			SKIN,PLCNT,GI		4 altered motifs		11 hits	AKAP9	intronic
7	92077067	0.8	0.96	rs200199046	T	TTTA	0.47	0.34	0.16	0.36	SKIN	VAS			7 altered motifs		8 hits	AKAP9	intronic
7	92077883	0.92	0.97	rs733957	C	T	0.50	0.38	0.17	0.39					5 altered motifs	6 hits	13 hits	AKAP9	intronic
7	92078306	0.9	0.97	rs112573247	T	TTTTAA	0.51	0.36	0.18	0.38					21 altered motifs		3 hits	AKAP9	intronic
7	92078466	0.92	0.97	rs12531561	A	G	0.49	0.38	0.17	0.39					6 altered motifs		10 hits	AKAP9	intronic
7	92078607	0.92	0.97	rs12540055	T	C	0.53	0.38	0.17	0.39					6 altered motifs		12 hits	AKAP9	intronic
7	92078905	0.88	0.97	rs41371748	T	C	0.05	0.28	0.08	0.37					HNF4,Pbx-1		5 hits	AKAP9	intronic
7	92081343	0.9	0.97	rs10271681	G	A	0.47	0.38	0.18	0.38					ERalpha-a,TCF11::MafG,ZID		11 hits	AKAP9	intronic
7	92081522	0.83	0.97	rs12539523	G	A	0.25	0.35	0.16	0.36					Ets,ZBTB33		8 hits	AKAP9	intronic
7	92081531	0.84	0.97	rs10245095	A	G	0.67	0.37	0.19	0.36					Sin3Ak-20,YY1		7 hits	AKAP9	intronic
7	92082152	0.93	0.97	rs2097739	C	T	0.49	0.38	0.17	0.39					p300		11 hits	AKAP9	intronic
7	92082477	0.93	0.97	rs2079082	A	G	0.49	0.38	0.17	0.39					5 altered motifs	6 hits	10 hits	AKAP9	intronic
7	92083384	0.93	0.97	rs6960867	A	G	0.25	0.36	0.17	0.39			ESDR		AP-1,HNF4		11 hits	AKAP9	missense
7	92084658	0.93	0.97	rs10228334	C	T	0.53	0.38	0.17	0.39					GR,Maf	1 hit	12 hits	AKAP9	synonymous
7	92086348	0.93	0.97	rs28927678	C	T	0.24	0.36	0.17	0.39					Mrg,NRSF,Tgif1		9 hits	AKAP9	synonymous
7	92086790	0.93	0.97	rs13239875	G	A	0.53	0.38	0.17	0.39					Maf,Myc,Pbx-1	6 hits	12 hits	AKAP9	intronic
7	92087637	0.93	0.97	rs10234290	T	C	0.50	0.38	0.17	0.39			HRT				12 hits	AKAP9	intronic
7	92088176	0.93	0.97	rs36021614	C	G	0.36	0.37	0.17	0.39							8 hits	AKAP9	intronic
7	92089032	0.93	0.97	rs12535601	A	G	0.50	0.38	0.17	0.39					4 altered motifs		11 hits	AKAP9	intronic
7	92089854	0.93	0.97	rs28584017	G	A	0.50	0.38	0.18	0.39					7 altered motifs		11 hits	AKAP9	intronic
7	92090294	0.93	0.97	rs4265	C	T	0.50	0.38	0.17	0.39					HMG-IY,Pou3f2	6 hits	13 hits	AKAP9	intronic
7	92090439	0.93	0.97	rs28708464	A	G	0.50	0.38	0.17	0.39					GR,LBP-1,YY1		11 hits	AKAP9	intronic
7	92091617	0.92	0.98	rs7811328	A	T	0.51	0.38	0.17	0.38					7 altered motifs		10 hits	AKAP9	intronic
7	92091888	0.94	0.98	rs138869164	TTA	T	0.29	0.37	0.17	0.39					ATF3,Mef2,PLZF		3 hits	AKAP9	intronic
7	92092267	0.95	0.98	rs12537846	A	G	0.50	0.38	0.17	0.39					HP1-site-factor,Hbp1,Pou1f1		13 hits	AKAP9	intronic
7	92093652	0.95	0.98	rs11982213	A	G	0.49	0.38	0.17	0.39					4 altered motifs		11 hits	AKAP9	intronic
7	92094377	0.93	0.98	rs12704634	C	T	0.49	0.38	0.18	0.39					4 altered motifs		9 hits	AKAP9	intronic
7	92097613	0.95	0.98	rs1063243	A	C	0.54	0.38	0.17	0.39					Evi-1,Hmbox1	6 hits	12 hits	AKAP9	synonymous
7	92098071	0.95	0.98	rs6946356	G	A	0.50	0.38	0.17	0.39					8 altered motifs		13 hits	AKAP9	intronic
7	92101733	0.95	0.98	rs9691325	G	A	0.53	0.38	0.17	0.39			SKIN		AP-1		10 hits	AKAP9	intronic
7	92102088	0.92	0.98	rs9691343	G	A	0.30	0.37	0.17	0.38					ERalpha-a,TFII-I,ZBRK1		8 hits	AKAP9	intronic
7	92102160	0.92	0.98	rs60768469	ATT	7-mer	0.53	0.37	0.17	0.38							7 hits	AKAP9	intronic
7	92102204	0.96	0.98	rs56075286	G	A	0.69	0.39	0.19	0.39					18 altered motifs		10 hits	AKAP9	intronic
7	92104351	0.98	0.99	rs4464887	C	T	0.25	0.37	0.18	0.39					CACD,TCF12,ZEB1		6 hits	AKAP9	intronic
7	92104515	0.94	1	rs4512319	G	A	0.19	0.30	0.08	0.38					4 altered motifs		7 hits	AKAP9	intronic
7	92105238	0.99	0.99	rs2075881	T	C	0.69	0.40	0.19	0.39			ADRL				12 hits	AKAP9	intronic
7	92105985	0.99	0.99	rs9690778	A	G	0.50	0.38	0.17	0.39					Lmo2-complex,RP58,SIX5		10 hits	AKAP9	intronic
7	92106299	0.99	0.99	rs9690208	T	A	0.53	0.38	0.17	0.39					7 altered motifs		10 hits	AKAP9	intronic
7	92106371	0.99	0.99	rs34987141	GA	G	0.48	0.38	0.17	0.39					NF-AT1,Pou5f1		3 hits	AKAP9	intronic
7	92106418	0.98	0.99	rs141268708	11-mer	A	0.50	0.38	0.17	0.39					4 altered motifs		3 hits	AKAP9	intronic
7	92106764	0.98	0.99	rs57816416	A	C	0.50	0.38	0.17	0.39					6 altered motifs		11 hits	AKAP9	intronic
7	92106784	0.99	0.99	rs60147309	C	G	0.53	0.38	0.17	0.39					MZF1::1-4,STAT		10 hits	AKAP9	intronic
7	92107480	0.99	0.99	rs7785971	T	A	0.69	0.40	0.19	0.39						1 hit	11 hits	AKAP9	intronic
7	92107560	0.99	0.99	rs7802788	A	G	0.69	0.40	0.19	0.39					4 altered motifs		9 hits	AKAP9	intronic
7	92107607	0.99	0.99	rs10257832	C	T	0.51	0.38	0.17	0.39					SETDB1,ZBRK1		10 hits	AKAP9	intronic
7	92107807	0.97	0.99	rs10273849	T	C	0.53	0.38	0.17	0.39					4 altered motifs		9 hits	AKAP9	intronic
7	92108010	0.99	0.99	rs12704635	T	A,G	0.49	0.38	0.17	0.39							11 hits	AKAP9	intronic
7	92111482	0.99	1	rs10274756	G	A	0.53	0.38	0.17	0.39					9 altered motifs		11 hits	668bp 3' of CYP51A1	
7	92111818	0.99	1	rs10231496	T	C	0.69	0.40	0.19	0.39			BLD		27 altered motifs		11 hits	332bp 3' of CYP51A1	
7	92112657	1	1	rs1135217	C	T	0.50	0.38	0.17	0.39					NF-kappaB		11 hits	CYP51A1	3'-UTR
7	92112873	1	1	rs11459	A	G	0.49	0.38	0.17	0.39					4 altered motifs		11 hits	CYP51A1	3'-UTR
7	92113034	1	1	rs8039	C	A	0.53	0.38	0.17	0.39					6 altered motifs		3 hits	CYP51A1	3'-UTR
7	92113288	1	1	rs6465348	A	G	0.48	0.38	0.17	0.39					TATA		12 hits	CYP51A1	3'-UTR
7	92113414	0.99	1	rs7793861	C	G	0.50	0.38	0.17	0.39			BLD		4 altered motifs		11 hits	CYP51A1	3'-UTR
7	92113836	1	1	rs7797834	A	G	0.49	0.38	0.17	0.39					4 altered motifs	1 hit	13 hits	CYP51A1	synonymous
7	92114997	0.98	1	rs6970743	C	G	0.49	0.38	0.18	0.40			BLD,BLD		GATA,Mef2		11 hits	CYP51A1	intronic
7	92116127	1	1	rs35653487	C	G	0.49	0.38	0.17	0.39					4 altered motifs		11 hits	CYP51A1	intronic
7	92117413	1	1	rs2301559	T	C	0.49	0.38	0.17	0.39					TATA	6 hits	13 hits	CYP51A1	intronic
7	92117806	0.99	1	rs10278080	G	A	0.49	0.38	0.17	0.39					Foxp1,TAL1		11 hits	CYP51A1	intronic
7	92120484	0.99	1	rs6973792	C	A	0.37	0.37	0.17	0.39		BRN, SKIN, LIV	IPSC,BLD		Irf		8 hits	CYP51A1	intronic
7	92120702	0.99	1	rs6956431	T	C	0.53	0.38	0.17	0.39		SKIN, LIV			22 altered motifs		10 hits	CYP51A1	intronic
7	92120793	0.99	1	rs6956596	T	G	0.25	0.36	0.17	0.39		SKIN, LIV	ADRL		Pou1f1		10 hits	CYP51A1	intronic
7	92120802	0.99	1	rs6974607	G	A	0.25	0.36	0.17	0.39		SKIN, LIV	ADRL		Pdx1		10 hits	CYP51A1	intronic
7	92121060	0.98	1	rs2106170	C	T	0.29	0.38	0.17	0.39		SKIN, BRN, LIV			Zfx		8 hits	CYP51A1	intronic
7	92121136	1	1	rs2106171	A	G	0.49	0.38	0.18	0.39		SKIN, LIV			6 altered motifs		11 hits	CYP51A1	intronic
7	92121264	1	1	rs2157747	A	G	0.53	0.37	0.17	0.39		SKIN, LIV			SREBP		12 hits	CYP51A1	intronic
7	92121806	1	1	rs7811873	G	A	0.49	0.38	0.17	0.39		SKIN	BLD,SKIN		9 altered motifs		11 hits	CYP51A1	intronic
7	92122348	0.99	1	rs61244255	C	T	0.36	0.37	0.17	0.39		SKIN			7 altered motifs		8 hits	CYP51A1	intronic
7	92123376	1	1	rs2269727	C	A	0.49	0.38	0.17	0.39					Arid3a,Hlx1,Maf	6 hits	13 hits	CYP51A1	intronic
7	92124265	1	1	rs10488512	C	T	0.53	0.38	0.17	0.39					Hand1		12 hits	CYP51A1	intronic
7	92128079	1	1	rs12533199	C	T	0.49	0.38	0.17	0.39			SKIN		BHLHE40		11 hits	CYP51A1	intronic
7	92128165	0.99	1	rs12538325	A	G	0.25	0.36	0.17	0.39		SKIN	BLD,SKIN,SKIN		STAT		7 hits	CYP51A1	intronic
7	92128797	0.99	1	rs7784248	A	G	0.54	0.38	0.17	0.39			SKIN	CTCF	AP-3		10 hits	CYP51A1	intronic
7	92130283	1	1	rs4644173	A	G	0.69	0.40	0.19	0.39		BRST, ADRL, LIV	SKIN		TATA,ZEB1	5 hits	12 hits	CYP51A1	intronic
7	92132303	0.99	1	rs2282974	G	A	0.50	0.38	0.17	0.39	17 tissues	9 tissues			NF-kappaB,TFIIA,THAP1		11 hits	CYP51A1	intronic
7	92132324	0.99	0.99	rs2282975	A	G	0.53	0.38	0.17	0.39	17 tissues	9 tissues			Foxi1,YY1		10 hits	CYP51A1	intronic
7	92132831	0.94	1	rs35968894	A	G	0.05	0.28	0.08	0.38	21 tissues	7 tissues	4 tissues		CTCF,Mef2		7 hits	CYP51A1	intronic
7	92133315	1	1	rs55801687	C	A	0.49	0.38	0.17	0.39	22 tissues	4 tissues	8 tissues	CEBPB	CEBPA,CEBPB,CEBPD		11 hits	CYP51A1	intronic
7	92134814	0.93	0.98	rs4279	C	A	0.51	0.38	0.18	0.40	24 tissues		53 tissues	44 bound proteins	Hdx,NRSF,Sin3Ak-20		12 hits	CTB-161K23.1	
7	92136325	0.96	0.98	rs6971318	T	C	0.65	0.39	0.20	0.39					Mef2		9 hits	CTB-161K23.1	intronic
7	92136526	0.87	0.96	rs12531966	A	G	0.27	0.36	0.19	0.38					7 altered motifs		6 hits	CTB-161K23.1	intronic
7	92137008	0.97	0.99	rs9656003	A	C	0.70	0.40	0.20	0.39					4 altered motifs		10 hits	CTB-161K23.1	intronic
7	92137639	0.96	0.98	rs11984136	T	C	0.68	0.40	0.20	0.39		ESDR, BLD, GI	ESDR,KID	GATA2			10 hits	CTB-161K23.1	intronic

7	92139301	0.96	0.98	rs6465350	G	A	0.49	0.38	0.18	0.39						4 altered motifs			10 hits	CTB-161K23.1	intronic
7	92139904	0.87	0.96	rs6953816	A	C	0.65	0.37	0.20	0.38						4 altered motifs			6 hits	CTB-161K23.1	intronic
7	92140263	0.95	0.97	rs7787995	G	A	0.65	0.39	0.20	0.39						CDP			9 hits	CTB-161K23.1	intronic
7	92141177	0.87	0.97	rs36032528	G	A	0.16	0.29	0.08	0.37						4 altered motifs			7 hits	CTB-161K23.1	intronic
7	92141391	0.96	0.98	rs28671255	A	G	0.69	0.40	0.20	0.39				BLD		Eomes,Sox,TBX5			10 hits	CTB-161K23.1	intronic
7	92142842	0.93	0.97	rs6951987	T	C	0.49	0.38	0.18	0.39			ESDR		POL2	AP-1,FAC1,Pax-6			8 hits	CTB-161K23.1	intronic
7	92142867	0.96	0.98	rs11971798	G	A	0.49	0.38	0.18	0.39			ESDR		POL2	Maf,VDR			10 hits	CTB-161K23.1	intronic
7	92143416	0.96	0.98	rs35417517	CG	C	0.65	0.40	0.20	0.39						7 altered motifs			3 hits	CTB-161K23.1	intronic
7	92143631	0.93	0.98	rs6974827	C	T	0.64	0.40	0.20	0.40						6 altered motifs			10 hits	CTB-161K23.1	intronic
7	92143674	0.88	0.95	rs6975248	G	A	0.44	0.38	0.18	0.39						Hic1,Mxi1,Myc			10 hits	CTB-161K23.1	intronic
7	92145803	0.96	0.98	rs6465352	A	G	0.65	0.40	0.20	0.39						4 altered motifs			11 hits	CTB-161K23.1	intronic
7	92147273	0.96	0.98	rs12540868	G	A	0.49	0.38	0.18	0.39						AP-1			10 hits	CTB-161K23.1	intronic
7	92148994	0.96	0.98	rs6973672	A	G	0.68	0.40	0.21	0.39						Zfx			10 hits	CTB-161K23.1	intronic
7	92149151	0.95	0.98	rs6973896	A	G	0.65	0.40	0.20	0.39						4 altered motifs			9 hits	CTB-161K23.1	intronic
7	92149703	0.95	0.98	rs13230837	A	G	0.36	0.37	0.18	0.39						26 altered motifs			9 hits	CTB-161K23.1	intronic
7	92149928	0.96	0.98	rs1978061	G	A	0.65	0.40	0.20	0.39				BLD	CEBPB	Smad			9 hits	CTB-161K23.1	intronic
7	92150657	0.96	0.98	rs6465353	T	G	0.70	0.40	0.20	0.39						CIZ,Foxa			11 hits	CTB-161K23.1	missense
7	92152066	0.94	0.97	rs6972027	T	C	0.64	0.38	0.19	0.39						9 altered motifs			9 hits	CTB-161K23.1	intronic
7	92152960	0.95	0.98	rs11976412	G	A	0.24	0.36	0.18	0.39			SKIN			6 altered motifs			8 hits	CTB-161K23.1	intronic
7	92153848	0.96	0.98	rs9770444	T	G	0.64	0.40	0.20	0.39						7 altered motifs			9 hits	CTB-161K23.1	intronic
7	92154898	0.96	0.98	rs7783674	T	A	0.65	0.40	0.20	0.39						GR,Gfi1b			9 hits	CTB-161K23.1	intronic
7	92155067	0.96	0.98	rs7801058	G	A	0.48	0.38	0.18	0.39									10 hits	CTB-161K23.1	intronic
7	92156643	0.96	0.98	rs7783414	C	T	0.69	0.40	0.20	0.39			4 tissues			GATA,YY1			12 hits	CTB-161K23.1	intronic
7	92156656	0.96	0.98	rs10271174	A	G	0.65	0.40	0.20	0.39						CEBPB,HNF4		3 hits	11 hits	CTB-161K23.1	intronic
7	92167164	0.93	0.97	rs7791282	T	C	0.27	0.37	0.18	0.39						ERalpha-a,STAT,TLX1::NFIC			8 hits	CTB-161K23.1	intronic
7	92169602	0.94	0.97	rs34040612	G	T	0.36	0.37	0.18	0.39						CEBPB			9 hits	CTB-161K23.1	intronic
7	92177839	0.91	0.96	rs4626516	G	C	0.24	0.36	0.18	0.39			7 tissues	ADRL		4 altered motifs			10 hits	CTB-161K23.1	intronic
7	92179185	0.91	0.96	rs10231350	G	T	0.65	0.39	0.20	0.39				48 tissues	9 bound proteins	12 altered motifs			9 hits	CTB-161K23.1	
7	92179601	0.92	0.96	rs10953065	G	A	0.27	0.37	0.18	0.39				20 tissues		PRDM1			11 hits	CTB-161K23.1	
7	92180343	0.92	0.97	rs2040499	C	G	0.66	0.40	0.20	0.39		BRN, MUS	5 tissues	BLD,MUS,VAS		Rad21		1 hit	12 hits	CTB-161K23.1	
7	92183721	0.92	0.96	rs13233496	T	C	0.35	0.37	0.19	0.39						10 altered motifs			9 hits	3kb 3' of CTB-161K23.1	
7	92194573	0.92	0.96	rs58673519	T	G	0.27	0.37	0.18	0.39						7 altered motifs			8 hits	4.4kb 3' of KRIT1	
7	92206122	0.92	0.96	rs34344935	G	A	0.24	0.36	0.18	0.39			ESDR		CTCF,RAD21				8 hits	KRIT1	intronic
7	92216130	0.91	0.97	rs28728062	G	C	0.50	0.38	0.19	0.40						4 altered motifs			7 hits	KRIT1	intronic
7	92218236	0.92	0.96	rs34574955	A	C	0.28	0.37	0.18	0.39						Foxp1,Nanog,Sox			7 hits	KRIT1	intronic
7	92221264	0.86	0.95	rs62467794	C	T	0.05	0.27	0.08	0.38						NF-kappaB,SP1,ZBTB33			7 hits	KRIT1	intronic
7	92245372	0.8	0.91	rs12704637	C	T	0.05	0.28	0.08	0.38		24 tissues		44 tissues	CTCF,RPC155,POL2				8 hits	KRIT1	intronic

Query SNP: rs9610915 and variants with r^2 >= 0.8

chr	pos (hg38)	LD (r^2)	LD (D')	variant	Ref	Alt	AFR freq	AMR freq	ASN freq	EUR freq	SiPhy cons	Promoter histone marks	Enhancer histone marks	DNAse	Proteins bound	Motifs changed	NHGRI/EBI GWAS hits	GRASP QTL hits	Selected eQTL hits	GENCODE genes	dbSNP func annot
22	38176433	0.8	-0.95	rs4384	G	C	0.62	0.51	0.37	0.43		SKIN, MUS, BLD	20 tissues	40 tissues	19 bound proteins	8 altered motifs			13 hits	PLA2G6	intronic
22	38204588	0.84	0.98	rs2267374	A	T	0.19	0.49	0.52	0.57		10 tissues	17 tissues	7 tissues		9 altered motifs			12 hits	MAFF	intronic
22	38206133	0.82	0.98	rs11914181	T	C	0.57	0.51	0.66	0.58		FAT	11 tissues	4 tissues		SP2,ZID			14 hits	MAFF	intronic
22	38209126	0.87	0.97	rs9607518	C	T	0.64	0.50	0.53	0.56			BLD, PLCNT, LNG			Pax-5,RFX5,Zbtb3			15 hits	MAFF	intronic
22	38210982	0.88	0.98	rs8139952	C	A	0.16	0.47	0.52	0.56			9 tissues	5 tissues		4 altered motifs			13 hits	MAFF	intronic
22	38211387	0.87	0.97	rs13055114	C	T	0.63	0.51	0.53	0.56			6 tissues			CACD,NRSF			16 hits	MAFF	intronic
22	38212220	0.84	0.97	rs2064103	T	C	0.67	0.50	0.66	0.57			4 tissues			BDP1,GCNF,Nr2f2			12 hits	MAFF	intronic
22	38213943	0.88	0.98	rs2235264	G	A	0.13	0.47	0.53	0.56		4 tissues	18 tissues	16 tissues		Pou2f2			12 hits	MAFF	intronic
22	38215073	1	1	rs9610915	C	G	0.18	0.46	0.53	0.54		5 tissues	18 tissues	23 tissues	POL24H8,TAF1	4 altered motifs			12 hits	MAFF	3'-UTR
22	38216597	0.89	0.98	rs34066050	G	A	0.16	0.47	0.53	0.56			BRST, BLD, PLCNT	7 tissues	POL24H8	4 altered motifs			12 hits	85bp 3' of MAFF	
22	38217761	0.88	0.99	rs9622757	G	C	0.16	0.47	0.53	0.57			4 tissues	16 tissues		Roaz,ZBRK1			12 hits	1.2kb 3' of MAFF	
22	38218122	0.82	0.98	rs4821767	C	A	0.16	0.48	0.53	0.58		GI, MUS	20 tissues	14 tissues		Hsf			16 hits	1.2kb 3' of TMEM184B	
